# A hybrid Prairie INFO fission naked algorithm with stagnation mechanism for the parametric estimation of solar photovoltaic systems

**DOI:** 10.1038/s41598-024-61434-3

**Published:** 2025-02-01

**Authors:** Pankaj Sharma, Rohit Salgotra, Saravanakumar Raju, Mohamed Abouhawwash, S. S. Askar

**Affiliations:** 1 School of Electrical Engineering, Vellore Institute of Technology, Vellore, India; 2 Faculty of Physics and Applied Computer Science, AGH University of Kraków, Kraków, Poland; 3 Data Science Institute, University of Technology Syndey, 15 Broadway, Ultimo, 2007 Australia; 4 Department of Animal Science, Michigan State University, East Lansing, 48824 MI USA; 5 Department of Mathematics, Faculty of Science, Mansoura University, Mansoura, 35516 Egypt; 6 Department of Statistics and Operations Research, College of Science, King Saud University, P.O. Box 2455, 11451 Riyadh, Saudi Arabia

**Keywords:** Energy science and technology, Mathematics and computing

## Abstract

This paper presents a study to enhance the performance of a recently introduced naked mole-rat algorithm (NMRA), by local optima avoidance, and better exploration as well as exploitation properties. A new set of algorithms, namely Prairie dog optimization algorithm, INFO, and Fission fusion optimization algorithm (FuFiO) are included in the fundamental framework of NMRA to enhance the exploration operation. The proposed algorithm is a hybrid algorithm based on four algorithms: Prairie Dog, INFO, Fission Fusion and Naked mole-rat (PIFN) algorithm. Five new mutation operators/inertia weights are exploited to make the algorithm self-adaptive in nature. Apart from that, a new stagnation phase is added for local optima avoidance. The proposed algorithm is tested for variable population, dimension size, and efficient set of parameters is analysed to make the algorithm self-adaptive in nature. Friedman as well as Wilcoxon rank-sum tests are performed to determine the effectiveness of the PIFN algorithm. On the basis of a comparison of outcomes, the PIFN algorithm is more effective and robust than the other optimization techniques evaluated by prior researchers to address standard benchmark functions (classical benchmarks, CEC 2017, and CEC-2019) and complex engineering design challenges. Furthermore, the effectiveness as well as reliability of the PIFN algorithm is demonstrated by testing using various PV modules, namely the RTC France Solar Cell (SDM, and DDM), Photowatt-PWP201, STM6- 40/36, and STP6-120/36 module. The results obtained from the PIFN algorithm are compared with various MH algorithms reported in the existing literature. The PIFN algorithm achieved the lowest root-mean-square error value, for RTC France Solar Cell (SDM) is 7.72E−04, RTC France Solar Cell (DDM) is 7.59E−04, STP6-120/36 module is 1.44E−02, STM6-40/36 module is 1.723E−03, and Photowatt-PWP201 module is 2.06E−03, respectively. In order to enhance the accuracy of the obtained results of parameter estimation of solar photovoltaic systems, we integrated the Newton-Raphson approach with the PIFN algorithm. Experimental and statistical results further prove the significance of the PIFN algorithm with respect to other algorithms.

## Introduction

The process of selecting the best solution (optimal or close to optimal) from a wide collection of feasible solutions for a specific problem is known as optimization. Mostly, real-world problems are used as a problem for optimization. However, real-world optimization challenges occur in almost all scientific areas, and engineering applications can be readily transformed into optimization problems. These challenges provide a number of issues and difficulties, including multi-objectivity, high dimensionality, non-linearity, uncertainty, discontinuity, and non-convex. Meta-heuristics (MH), as well as mathematical programming, are two subcategories of optimization approaches. The mathematical approach is an old approach and is unsuccessful in solving such challenges due to its complexity. The other category is the MH approach, which is inspired by natural phenomena or behaviour. MH can identify optimal or nearly optimal solutions due to its benefits, including its simplicity in usage, adaptability, ability to avoid traps in local optima, and black box functionality. As a result, the optimization techniques proposed in the last few years have increased exponentially^1^. There are several MH categories in the literature. For instance, there are four different types of MH algorithms (Swarm, Human, Physics, and Evolutionary-based)^2–5^.

The term “evolutionary-based algorithm” (EA) refers to stochastic population-based algorithms that take essential inspirations from nature and use genetics concepts, including selection, crossover, mutation, and elimination. The various example of EA is genetic algorithm (GA)^6^, memetic algorithm (MA)^7^, scatter search (SS)^8^, stochastic fractal search (SFS)^9^, cooperative memetic algorithm with feedback (CMAF)^3^, and bull optimization algorithm (BOA)^10^.

The physics-based algorithm is inspired by the physics laws that exist in the universe. This type includes several algorithms, like intelligent water drops (IWD)^11^, black hole (BH)^12^, optics-inspirited optimization (OIO)^13^, light spectrum optimizer (LSO)^14^, electromagnetic field optimization (EFO)^15^, atom search optimization (ASO)^16^, archimedes optimization algorithm (AOA)^17^, homonuclear molecules optimization (HMO)^18^, lichtenberg algorithm (LA)^19^, spring search algorithm (SSA)^20^, momentum search algorithm (MSA)^21^, material generation algorithm (MGA)^22^, special relativity search (SRS)^23^, cooperative water wave optimization (CWWO)^24^ etc.

The intelligent, coordinated, as well as social behaviour of swarms or communities, including flocks of birds, insect colonies like herds of animals, and many more flocks of various species, is imitated by optimization algorithms in this type. This type includes several algorithms, like particle swarm optimization (PSO)^25^, symbiotic organisms search (SOS)^26^, elephant search algorithm (ESA)^27^, grasshopper optimization algorithm (GOA)^28^, sandpiper optimization algorithm (SOA)^29^, red fox optimization algorithm (RFO)^30^, golden eagle optimizer (GEO)^31^, clouded leopard optimization (CLO)^32^, mud ring algorithm (MRA)^33^, hermit crab shell exchange (HCSE)^34^, sea horse optimizer (SHO)^35^, escaping bird search (EBS)^36^, honey badger algorithm (HBA)^37^, artificial lizard search optimization (ALSO)^38^, grey wolf optimization (GWO)^39^, crow search algorithm (CSA)^40^, pareto-based discrete Jaya algorithm (PDJaya)^4^ and many other MH algorithms^41^.

The last type of MH algorithm is the human based algorithm, which is inspired by the behaviour of the human in nature, such as human behaviour-based optimization (HBBO)^42^, search and rescue optimization algorithm (SAR)^43^, learner performance based behavior (LPB)^44^, human felicity algorithm (HFA)^45^, city councils evolution (CCE)^46^, election-based optimization algorithm (EBOA)^47^, ali baba and the forty thieves (AFT)^48^, student psychology based optimization algorithm (SPBO)^49^, knowledge-based two-population optimization (KTPO)^50^ and queuing search (QS)^51^.

Naked mole-rat algorithm (NMRA) is a recently presented algorithm that has shown its utility for a variety of domain research challenges^52^. This algorithm is inspired by the mating patterns of NMRA and uses a worker-breeder relationship to formulate the NMRA. It has two phases, a worker phase to control the exploration operation as well as the breeder stage for exploitation. The algorithm uses two random solutions in the worker phase for searching for new solutions and one random solution working in the proximity of the best-guided solution for the breeder phase. A random probability parameter is also included to control the extent of exploration as well as exploitation operation. Overall, the algorithm is efficient and has a simple structure. But when using it for problems of higher dimensions, it becomes very difficult to optimize, and we need to make modifications. Apart from this, local optima stagnation is also a major problem in most of the algorithms and NMRA also suffers from the same^53^. Also, in existing literature, the balanced interplay between two critical aspects, namely exploration and exploitation, holds significant importance. This equilibrium mandates that both exploration and exploitation are pursued simultaneously, aiming to prevent an algorithm from becoming overly focused on either extensive exploration or excessive exploitation of the search space. An adept algorithm adheres to a strategic sequence: commencing with thorough exploration in its initial phase, transitioning towards concentrated exploitation during the middle phase, promoting a blend of exploration and focused exploitation as it progresses, and finally with a phase of thorough exploitation and intensive exploration in the later stages. Despite these guiding principles, there remains a prevailing concern; as of now, no algorithm has emerged that adeptly executes these operations with remarkable efficiency^53^. The integration of multiple algorithms offers a range of advantages, such as favourable convergence attributes and improved global search capabilities that facilitate the discovery of multiple outcomes for intricate challenges.

So, in the present work, a new hybrid variant of NMRA is proposed. This variant uses a combination of three newly proposed algorithms namely Prairie dog optimization algorithm (PDOA)^54^, fission-fusion optimization algorithm (FuFiO)^55^, INFO^56^ for enhancing the worker phase of the algorithm. All of these added algorithms use some specifically designed set of equations for each of the formulated phases. The primary motivation for selecting the Flower pollination algorithm (FPA) as the foundational algorithm stems from its straightforward implementation as well as clear structure. This algorithm also encompasses a well-defined balance between exploration as well as exploitation operations, regulated by a switch probability. Incorporating new algorithmic equations into NMRA yields an enhanced performance. PDO, INFO, and FuFiO algorithm demonstrates notable efficiency in exploration due to their robustly defined exploration phase^54–56^. Lastly, mutation operators are added which contributes in balancing properties. By leveraging all these algorithms, a novel approach is created that optimally combines their equations to formulate an algorithm with comprehensive benefits.

In the present work, we use only those equations which fit the best for our model, and all other equations from the basic algorithms are discarded. To address the challenges associated with local optimal stagnation, a new stagnation phase is designed. This kind of modification is new to single-objective optimization algorithms, and very limited work is done in this domain. Apart from these enhancements, we added inertia weights/mutation operators to make all the parameters of the proposed algorithm self-adaptive in nature. The performance analysis of the PIFN algorithm is conducted using the classical benchmarks^57^, CEC 2017^58^, and CEC 2019 benchmarks^59,60^. A comparison is carried out between the well-known MH optimization and recently developed MH optimization algorithms such as multi-hybrid algorithm (MHA)^61^, adaptive differential evolution with optional external archive (JADE)^62^, self-adaptive DE algorithm (jDE100)^63^ (winner algorithm of CEC 2019), Young’s double-slit experiment optimizer (YDSE)^64^, success-history-based adaptive DE (SHADE)^65^, Evolution strategy with covariance adaptation (CMA-ES)^66^, fractional-order calculus-based flower pollination algorithm (FA-FPO)^67^, Extended GWO (GWO- E)^68^, Sine Cosine CSA (SCCSA)^69^, equilibrium optimizer (EO)^70^, and SHADE with linear population size reduction hybrid with semi-parameter adaptation of CMA-ES (LSHADE-SPACMA)^70^ . The statistical significance of the proposed algorithm has been evaluated using two commonly employed tests, namely the Wilcoxon rank-sum test and the Friedman test. In addition to evaluating the algorithm’s performance through benchmark results, the algorithm is subjected to the real world application parameter estimation of solar PV.

The integration of solar photovoltaic systems into the modern power grid offers a variety of new obstacles. The precise modeling of PV systems is essential to enhance the system’s characteristics in simulation environments. The precise estimation of the parameters of the PV cell and panel provides significant importance in accurately predicting their energy generation, quickly determining the maximum power point, and serves the needs of both manufacturers for quality control during production and researchers to improve quality, efficiency, and study the degradation of PV cells^71,72^. However, a significant challenge faced by researchers throughout the modeling process is the accurate estimation of the solar photovoltaic parameters. This is because the solar PV operating conditions and the model parameters are closely related. The modeling of solar PV systems has been recognized as a multivariable nature, a highly challenging task due to their nonlinear behavior, intrinsic complexity, as well as strong coupling characteristics^73^. Using traditional methods to simulate solar photovoltaic energy produces significant challenges. Due to their inherent flexibility and resilience, MH optimization algorithms have become the preferred method for modeling solar PV^74,75^. Several optimization strategies have been presented in the literature to precisely estimate the parameter of the solar PV model, including the improved carnivorous plant algorithm (I-CPA)^76^, tree seed algorithm (TSA)^77^, improved whale optimization algorithm^78^, DE^71^, Teaching-learning-based artificial bee colony (TLABC)^71,79^, flexible improved particle swarm optimization and sequential quadratic programming (FIPSO-SQP)^80^, AFT^81^, Q-learning JAYA algorithm (QLJAYA)^82^, hybrid flower grey differential (HFGD)^83^, bald eagle search (BES)^84^, gaining-sharing knowledge (GSK)^71^, honey badger algorithms (HBA), HBA-opposition based learning (HBA-OBL)^85^, hybrid flower grey differential (HFGD)^83^, mantis search algorithm (MSA)^86^, modified dwarf mongoose optimizer (MDMO)^87^, chimp optimization algorithm (ChOA)^88^, growth optimizer (GO)^89^, heap-based optimizer (HBO)^90^, bonobo optimizer (BO)^91^, genetic algorithm based on non-uniform mutation (GAMNU)^92^, enhanced social network search algorithm (ESNSA)^93^, supply demand-based optimization (SDO)^94^, and artificial hummingbird technique (AHT)^95^.

The main contribution of the paper is as follows:

- In this article, a multi-hybrid algorithm-based Prairie Dog, INFO, and Fission fusion optimization algorithm in order to overcome the problem of poor exploration of NMRA known prairie Dog, INFO, Fission Fusion, and Naked mole-rat (PIFN) algorithm.
- A new stagnation phase is added to overcome the local optima stagnation problem.
- The proposed algorithm is subjected to parametric adaptations to make it self-adaptive in nature.
- The algorithm has been tested on Classical, CEC 2017, and CEC 2019 benchmark problems.
- The algorithm has been applied to highly complex real-world engineering design parametric estimations of PV systems (RTC France Solar Cell (SDM, and DDM), Photowatt-PWP201, STM6- 40/36, and STP6-120/36 module). Additionally, the results obtained from the PIFN algorithm are compared with various MH algorithms reported in the existing literature.
- Statistical tests (Friedman’s as well as Wilcoxon’s signed-rank test), are utilized in order to assess the importance of statistical significance between PFIN and other MH optimization techniques.

**Figure 1:**
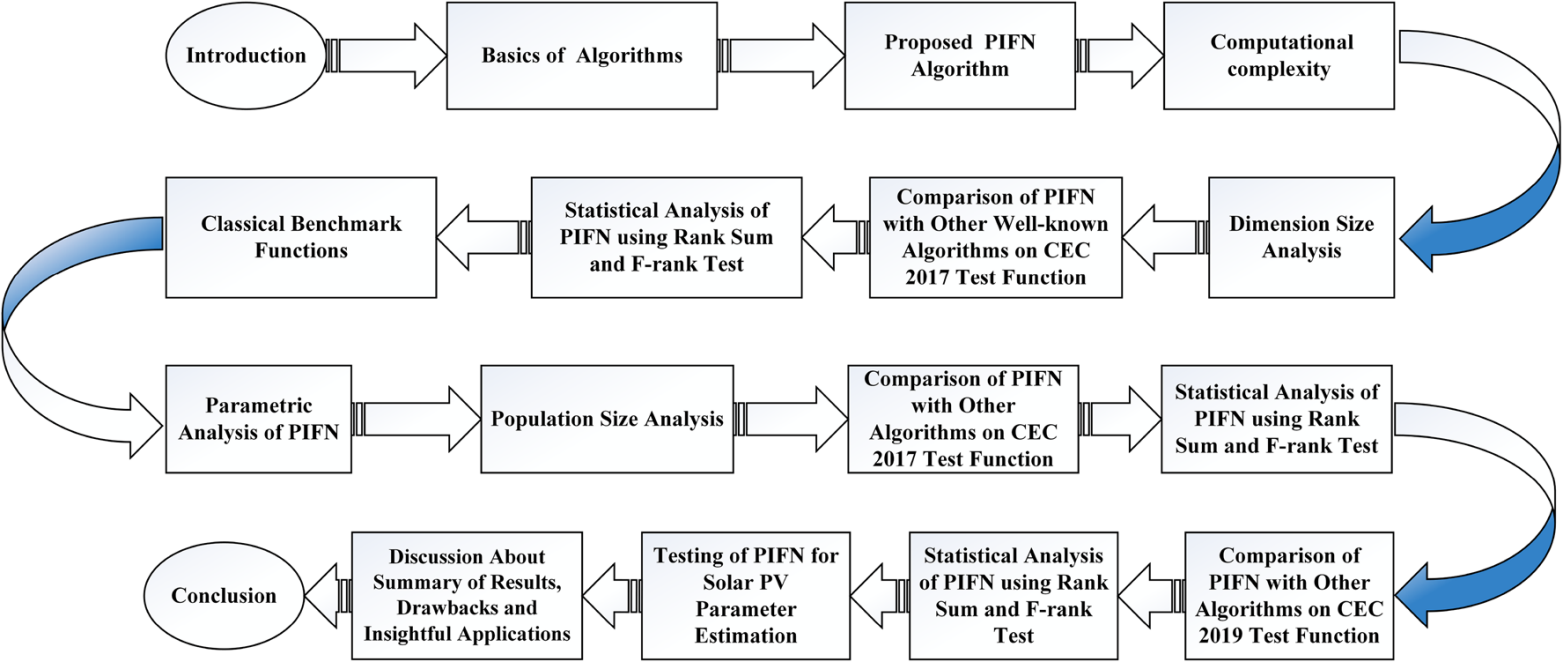
Outline of the article.

The rest of the paper is divided into 7 more sections. Here “section Basics of nature inspired algorithms” details about the basic algorithms used in designing the new algorithm, and “section The proposed: approach” formulates a new algorithm based on the concepts of multi-algorithm hybridization, self-adaptation, and local optima avoidance. In “section Computational complexity”, the computational complexity of the proposed algorithm is presented. In “section Result on benchmark problems”, experimental results with respect to other algorithms are presented. We have used two benchmark sets, namely classical benchmarks^57^, CEC 2017^58^, and CEC 2019 benchmarks^59,60^. All of these benchmarks consist of challenging problems and are unimodal, multimodal, hybrid, and composite in nature. Apart from that, the algorithms used for comparison are some of the most recently introduced algorithms and include adaptive differential evolution with optional external archive (JADE)^62^, success-history-based adaptive DE (SHADE)^65^, and SHADE with linear population size reduction hybrid with semi-parameter adaptation of CMA-ES (LSHADE-SPACMA)^70^ among others. In “section Parametric estimation of PV systems”, real-world optimization for the parameter estimation of the solar PV system is done, and it includes RTC France Solar Cell (SDM, and DDM), Photowatt-PWP201, STM6- 40/36, and STP6-120/36 module. The accurate modelling of the solar PV system has the greatest impact in the process of planning, optimizing, and operating the system. The main objective is to calculate the SDM of a PV cell’s five unknown variables are photo-voltaic current ($I_{Ph}$), diode saturation current ($I_{sc}$), shunt resistance ($R_{shunt}$), series resistance ($R_{s}$), and diode ideality factor (*n*). An additional diode has been included into the SDM, resulting in the formation of the DDM. The presence of an extra diode introduces space charge recombination current as well as introduces two new parameters, denoted as $I_{sc2}$ and $n_{2}$, for the second diode. The objective of parameter estimation is to reduce the error between the observed and simulated data. “Section Discussion of results”, details about the discussion, summary of results, drawbacks, as well as insightful implications. In the final section, the conclusion and future directions are outlined. Figure 1 contains an overview of the paper.

## Basics of nature inspired algorithms

In this section, we describe the fundamentals of the algorithms used for the proposal of our new hybrid algorithm. The new algorithms that are utilized include NMRA, PDOA, FuFiO, INFO and are explained as

### Naked mole-rat algorithm

This NMRA imitates the natural mating behaviours of NMRs. The NMRA is divided into three stages. The first stage is known as the initialization stage, the second stage is the worker, and the final stage is the breeder^52^.

#### Initialization

This stage is most commonly present in nature-inspired algorithms. Mostly, this is the first stage of the nature-inspired algorithm. In NMRA, the initial population range is $[1,2,3,4,5,6,\ldots ,n]$, and dimensional vector (D). The mathematical model is given as in the Eq. (1).

1 $$\begin{aligned} K_{i,j}=K_{min,j}+R(0,1)*(K_{min,j}-K_{max,j}) \end{aligned}$$

where, the variables $K_{min,j}$ and $K_{max,j}$ represent the lower as well as upper bounds, respectively, of a given function. The variable *i* is defined within the range from 1 to *n*, whereas the variable j is defined within the range from 1 to *D*. Following the initialization step, the fitness of the goal function is evaluated, and breeders (*B*) and workers (*W*) are determined.

#### Worker

Following the completion of the initialization stage, a determination of the fitness of the objective function. This evaluation serves as the basis for determining the breeders (*B*) and workers (*W*). In this stage, the workers enhance their fitness. So that their chances of becoming breeders and finally mating with the queen. Thus, the worker NMR generates a new solution based on experience. If the new solution is the best, the new best solution is replaced by the old one. Otherwise, use the old best solution. Finally, the mathematical model for the new solution is presented in the Eq. (2).

2 $$\begin{aligned} Z_{i}^{t+1}=Z_{i}^{t}+\lambda (Z_{j}^{t}-Z_{k}^{t}) \end{aligned}$$

where, new solution $(Z_{i}^{t+1})$, previous solution $(Z_{i}^{t})$, mating factor $(\lambda = [0,1])$, and randomly chosen solution $(Z_{j}^{t}-Z_{k}^{t})$ from the worker pool.

#### Breeder

In addition to being selected for mating as well as to remain a breeder, the NMR breeder must also keep himself updated. However, some of the breeders are not able to improve their fitness, so they may be pushed back to the worker pool. Therefore, the Eq. (3) gives the mathematical model for new breeder solutions.

3 $$\begin{aligned} P_{i}^{t+1}=(1-\lambda )P_{i}^{t}+\lambda (d-P_{i}^{t}) \end{aligned}$$

where, the parameter $\lambda$ (where $\lambda =[0,1]$) controls the frequency at which mating occurs between the breeder and the queen. The new solution at time $t+1$ is denoted as $P_{i}^{t+1}$, the overall best solution is represented by *d*, and the breeder solution at time *t* is denoted as $P_{i}^{t}$..

### Prairie Dog Optimization Algorithm

Prairie Dog Optimization Algorithm (PDOA) is a recently proposed algorithm based on the prairie dog’s behaviour^54^. The two typical optimization stages (Exploration and Exploitation) are achieved by the PDOA utilizing four prairie dog activities. Below is the mathematical model for implementing this behaviour for using the PDOA algorithm to perform optimization:

#### Exploration

The PDOA can navigate between exploration as well as exploitation depending on four factors. The maximum number of iterations is split into four parts: the exploration takes up the first two parts, as well as the exploitation takes up the remaining two parts. The two parts used for exploration are conditioned in the Eq. (4):

4 $$\begin{aligned} Iteration< \frac{maximum \;Iteration}{4} \; and \; \frac{maximum \;Iteration}{4}\leqslant Iteration< \frac{maximum \;Iteration}{2} \end{aligned}$$

The two parts used for exploitation are conditioned in the Eq. (5):

5 $$\begin{aligned} \frac{maximum\; Iteration}{2}\le iteration< 3*\left( \frac{maximum Iteration}{4} \right) \\ and \; 3 * \left( \frac{maximum \;Iteration}{4} \right) \le iteration \leqslant maximum \;Iteration \end{aligned}$$

In the exploration phase, the coterie’s first approach is to make members explore the ward for fresh meal sources. The Levy flying motion best captures the movement of the prairie dogs as they search for meals. When they have discovered the meal sources, they produce unique sounds to notify other members of the group. Then, in accordance with the quality of the meal supply, new burrows are created, and the best are selected for foraging. The location updating for foraging in the exploration part of the PDOA is presented in the Eq. (6).

6 $$\begin{aligned} PD_{i+1,j+1}=GBest_{i,j}-eCBest_{i,j}*\rho -CPD_{i,j}\;*\;Levy(n) \; \forall \;\;\; Iteration< \frac{Maximum\;Iteration}{4} \end{aligned}$$

The next approach assesses the strength of the digging and the quality of the meal sources so far. In order to build new burrows, the number of iterations is increased while the digging strength is intended to decrease. The position updating information for the burrow building is presented in the Eq. (7).

7 $$\begin{aligned} PD_{i+1,j+1}=GBest_{i,j}\;*r\;PD\;*\;DS\;*\;Levy(n) \; \forall \;\; Iteration< \frac{Maximum\;Iteration}{4}\le Iteration< \frac{Maximum\;Iteration}{2} \end{aligned}$$

where, global best $(GBest_{i,j})$ obtained solution, current obtained $(eCBest_{i,j})$ best solution, quality meal source $(\rho =0.1kHz)$ alarm, position (*rPD*) of a random solution, randomized cumulative $(CPD_{i,j})$ effect of all prairie dogs, coterie’s digging (*DS*), stochastic property $(r=[-1,1])$ and Levy distribution strength (*Levy*(*n*)).

#### Exploitation

In this second stage, the exploitative behaviour of PDOA is presented. In this PDOA, exploitation is carried out by utilizing the prairie dogs’ distinct responses to two different communication or alarm sounds. These two distinct behaviours lead to the prairie dogs converging on a particular location or a suitable location in the case of PDO implementation, where further search (exploitation) is performed to identify superior or nearly optimal solutions. The primary purpose of the exploitation techniques utilized in PDOA is to search the suitable locations discovered during the exploration stage extensively. Equations (9) and  (10) represent the two strategies employed in this stage.

8 $$\begin{aligned} PD_{i+1,j+1}=GBest_{i,j}-eCBest_{i,j}\;* \; \varepsilon -CPD_{i,j}\; * \; rand \; \end{aligned}$$

9 $$\begin{aligned} \forall \;\; \frac{Maxmimum \; Iteration}{2} \; \le Iteration\;< 3 \;*\;\left( \frac{Maximum \; Iteration}{4} \right) \end{aligned}$$

10 $$\begin{aligned} PD_{i+1,j+1}=GBest_{i,j}\;*PE\;*\;rand \;\forall \; 3\;*\; \left( \frac{Maxmimum\; Iteration}{4} \right) \le Iteration< Maximum\;Iteration \end{aligned}$$

where small number $(\varepsilon )$ for meal source quality, predator effect (*PE*), and random number $(rand=[0,1])$.

### Fission fusion algorithm

Fusion-fission optimization algorithm (FuFiOA) is a new MH algorithm which is based on the principle of nuclear reaction^55^. This algorithm distinguishes two categories of nuclei (stable and unstable). Each nucleus can use three different nuclear processes to interact with other nuclei (fusion, fission, and beta-decay). The first phase of population-based MH algorithms is initialization. The following is the mathematical representation of each reaction in each group:

#### Stable nucleus

One of the subsequent three reactions is randomly selected if the i-th nucleus is stable $(T_{i}^{stable})$.

Reaction 1: The i-th nucleus collides with a different stable nucleus in this process. The following Eq. (11) is used to determine the new position $(T_{i}^{new})$:

11 $$\begin{aligned} T_{i}^{new}=aT_{i}^{stable}+(1-a)T_{j}^{stable} \end{aligned}$$

where, random vector $(a=[0,1])$.

Reaction 2: This collision generates a new solution, when i-th nucleus collides with a different unstable $(T_{j}^{unstable})$ nuclei is presented in the given Eq. (12).

12 $$\begin{aligned} T_{i}^{new}=T_{i}^{stable}+a(T_{i}^{stable}-T_{j}^{unstable}) \end{aligned}$$

Reaction 3: The new solution will be generated If the i-th nucleus decays.

13 $$\begin{aligned} T_{i}^{new}=\left\{ \begin{matrix} T_{i}^{k} &{} k \not \in p\\ R^{k} &{}k\in p \end{matrix}\right. ,p \subseteq d\\ R=lb+a(ub-lb) \end{aligned}$$

where, *lb* and *ub* are the lower as well as upper limits, a subset of problem (*p*), counter (*k*), and set of all variable (*d*), and random nucleus (*R*).

#### Unstable nucleus

One of the subsequent three reactions is randomly selected if the i-th nucleus is stable $(T_{i}^{unstable})$.

Reaction 1: The i-th nucleus collides with a different unstable nucleus in this process. The following Eq. (14) is used to determine the new position $(T_{i}^{new})$:

14 $$\begin{aligned} T_{i}^{new}=T_{i}^{unstable}+a(T_{i}^{unstable}-T_{j}^{unstable}) \end{aligned}$$

Reaction 2: This collision generates a new solution, when i-th nucleus collides with a different stable $(T_{j}^{unstable})$ nuclei is presented in the given Eq. (15).

15 $$\begin{aligned} T_{i}^{new}=T_{i}^{unstable}+a(T_{j}^{unstable}-T_{i}^{stable}) \end{aligned}$$

Reaction 3:The new solution will be generated If the i-th unstable nucleus decays.

16 $$\begin{aligned} T_{i}^{new}=\left\{ \begin{matrix} T_{i}^{k} &{} k \not \in p\\ T_{j}^{k} &{}k\in p \end{matrix}\right. \end{aligned}$$

In both stable and unstable groups, the third reaction are the $(\beta \pm )$decays. An important point is that the $(\beta \pm )$decays are considered as mutation operators to avoid local optima.

### INFO Algorithm

This algorithm is a population-based MH optimization technique known as weIghted meaN oF vectOrs (INFO). The population consists of a collection of vectors that provide possible answers in this approach. The positions of the vectors are updated by the three operators for every generation, and their mathematical presentation of these operators are given below^56^:

#### Updating rule

Instead of shifting the current vector along the trajectory towards an improved solution, the INFO algorithm first employs a collection of randomly chosen differential vectors to calculate the weighted average of these vectors. Increasing population diversity is considered the mean rule depending on the best, better, and worst outcomes. The mathematical representations of Mean-Rule are presented in the Eq. (17).

17 $$\begin{aligned} MeanRule=(WM1_{l}^{g}*a)+(WM2_{l}^{g}+(1-a)) \\ where\;\;\; l=1,2,3,4,5,6,7,8\ldots ,Np \end{aligned}$$

In this INFO algorithm, convergence acceleration (CA) is also added. The optimal solution is considered the nearest to the global optimum. In actuality, CA encourages better vector movement. The mathematical representation of CA is presented in the Eq. 18.

18 $$\begin{aligned} CA\;\;=\;\;random*\frac{(x_{bs}-x_{a1})}{(f(x_{bs})-f(x_{a1})+\varepsilon )} \end{aligned}$$

19 $$\begin{aligned} if\;\;\; random < 0.5\\ Z1_{l}^{g}\;\;=\;\;x_{l}^{g}+\sigma *MeanRule+random*\frac{(x_{bs}-x_{a1})}{(f(x_{bs})-f(x_{a1})+1 )}\\ Z2_{l}^{g}\;\;=\;\;x_{bs}+\sigma *MeanRule+random*\frac{(x_{a1}^{g}-x_{b}^{g})}{(f(x_{a1}^{g})-f(x_{a2}^{g})}\\ else\\ Z1_{l}^{g}\;\;=\;\;x_{a}^{g}+\sigma *MeanRule+random*\frac{(x_{a2}^{g}-x_{a3}^{g})}{(f(x_{a2}^{g})-f(x_{a3}^{g})+1 )}\\ Z2_{i}^{g}\;\;=\;\;x_{bt}+\sigma *MeanRule+random*\frac{(x_{a1}^{g}-x_{a2}^{g})}{(f(x_{a1}^{g})-f(x_{a2}^{g})+1 )}\\ end \end{aligned}$$

where random number $$(a=[0,0.5])$$, and $$(random=[0,1])$$, new vector $$(Z1_{l}^{g}$$ and $$Z2_{l}^{g})$$, as well as scaling rate $$(\sigma )$$.

#### Vector combining

The two vectors calculated in previous subsection $$(Z1_{l}^{g}$$ and $$Z2_{l}^{g})$$ are combined with $$x_{l}^{g}$$ when random is less than 0.5, then new vector $$u_{l}^{g}$$ generate, presented in Eq. (20), where $$u=0.05*random$$.

20 $$\begin{aligned} if \;\;random< 0.5\\ if \;\;random< 0.5\\ u_{l}^{g}=Z1_{l}^{g}+u.\left| Z1_{l}^{g}-Z2_{l}^{g} \right| \\ else\\ u_{l}^{g}=Z2_{l}^{g}+u.\left| Z1_{l}^{g}-Z2_{l}^{g} \right| \\ end\\ else\\ u_{l}^{g}=x_{l}^{g}\\ end \end{aligned}$$

#### Local search stage

Effective local search features can avoid INFO from suffering for deception and settle for locally optimum outcomes. Although, this operator indicates that a unique vector can be generated at $x_{best}^{g}$ if $random< 0.5$, where rand is a random number[0, 1].

21 $$\begin{aligned} if \;\;random< 0.5\\ if \;\;random< 0.5\\ u_{l}^{g}=x_{bs}+random*(MeanRule+random*(x_{bs}^{g}-x_{a1}^{g}))\\ else\\ u_{l}^{g}=x_{random}+random*(MeanRule+random*(v_{1}-x_{bs}-v_{1}-x_{random}))\\ end\\ end \end{aligned}$$

## The proposed: approach

This section details about the new and novel proposal of Prairie dog, INFO, Fission fusion and NMRA hybrid algorithm. The algorithm is named as PIFN algorithm and uses the added merits of all of these algorithms. The major enhancements of the proposed algorithm include

- The basic structure of NMRA is followed to propose the hybrid algorithm. It uses four new algorithms, that are added to the worker phase of NMRA. New algorithms that are added, use a certain set of iterations and their best search equation for hybridization.
- The worker phase uses basic NMRA for first one-fourth iterations, PDOA for second, FuFiO for third, and INFO for the final phase.
- A new stagnation phase is presented in the algorithm for local optima avoidance. This phase is formulated by using the self-adaptive cuckoo search (SACS) inspired equations.
- Parametric adaptations are followed by using five different mutation operators and modifications are added to make all the parameters of the proposed PIFN, self-adaptive in nature.

In the next subsections, further information about the enhanced improvements is presented.

### Requirement of the proposal

NMRA is a recent introduction in the field of swarm intelligent algorithms and has proven good capabilities for hybridization. This can be better understood from the fact that the algorithm has a well established exploration and exploitation operation, which makes it very easy to hybridize. The major motivation to add such modification is the need of new and prospective equations for performance analysis based on the algorithm under consideration. On the other hand, based on the No Free Lunch (NFL) theory, no MH algorithm can successfully complete all types of optimization challenges^96^. In other words, although the optimization technique may provide outstanding outcomes for a certain class of problems, it may also be ineffective for solving other classes of problems. Thus, it becomes significantly challenging to see how new modifications can be added and what can be done to make it more efficient. Apart from that, NMRA do suffer from the problems of local optima stagnation and has a poor exploration operation^53^. So in order to mitigate these problems, a new set of equation modifications and enhanced stagnation phase is added. More details on the modification are provided in the next subsection.

### The proposed algorithm

In the proposed algorithm, we add PDOA, FuFiO, INFO and SACS based modifications into the worker phase of the algorithm. Here, equation modifications are added based on a certain set of iterations and each of these equations are used in such a way that they follow a pattern of extensive exploration to intensive exploitation. The exploration operation as a whole is enhanced with added advantages of some exploitation properties. The parameters are also made self-adaptive so that we do not have to modify the parameters of the algorithms. Also, the original structure of NMRA is kept as such and more details are presented in the consecutive subsections.

#### Initialization

The first phase of the algorithm is initialization and in this phase, random solutions are generated using the boundary conditions of the algorithm. These equations are generated mathematically by

22 $$\begin{aligned} x_{i,j}=L_b^{j}+r\times (L_b^{j}-U_b^{j}) \end{aligned}$$

where, the variable *i* is defined within the range of 1 to *n*, while the variable *j* is defined within the range of 1 to *D*. The lower bound of the problem under consideration is denoted as $L_b^{j}$, as well as the upper bound is denoted as $U_b^{j}$.

#### Worker phase

*a) Phase I: For Iterations* $\le$ $t_{max}/4$: The first of the four phases is meant for immediate exploration operation and requires higher level of randomization for the generation and evaluation of new solutions. Here, we use two random solutions as well as an adaptive parameter $\lambda$ is employed to search for potential solutions. The phase is exactly the general worker phase of NMRA and mathematical equations for this case are given by

23 $$\begin{aligned} x_{i}^{t+1}=x_{i}^{t}+\lambda (x_p^t-x_q^t) \end{aligned}$$

where $x_i^t$ represents $i$th solution with respect to the $t$th iteration, $x_i^{t+1}$ is the new solution obtained by utilizing two random solutions $x_p^t$ as well as $x_q^t$.

*b) Phase II: For *$t_{max}/4$ > ***iterations*** $\le$ $t_{max}/2$: PDO^54^ is a newly presented algorithm and has a dedicated exploration phase, meant specifically for better global search operation. The goal of this operation of PDO is to search for certain proximal sections of the search space. The strategy is modelled using two equations and in the basic algorithm, it is used based on a certain set of generations. In the present case, we are using these equations based on certain probabilities and mathematical formulations are given by

24 $$\ \begin{aligned} if\;rand < 0.5 \\ & if\;abs(A) \ge 1 \\ & x_{i}^{{t + 1}} = x_{{best}} - n_{{best}} \times l - x_{i}^{t} \times rand() \\ & else \\ & x_{i}^{{t + 1}} = x_{b} est \times ge \times rand() \\ & else \\ & if\;rand \ge 0.5 \\ & de = abs(x_{{best}} - x_{i}^{t} )x_{i}^{{t + 1}} \\ & = de \times exp(l). \times cos(l.2\pi ) + x_{{best}} \\ & end \\ \end{aligned}$$

where $n_{best}$ is the local best solution, $x_i^t$ is a current solution, l is the parameter of PDO and is made self-adaptive in nature using different mutation operators (discussed in consecutive sections), and *A* is a random number.

*c) Phase III: For* $t_{max}/2$ > ***iterations*** $\le$ $t_{max} 3/4$: This phase is meant for slow drift of solutions from exploration towards exploitation phase, and better exploration operation along with intensified exploitation is also required during this phase. So, vector combining phase of INFO algorithm^56^ is used for enhanced performance. The vector combining phase of INFO uses a combination of equations which are better suited for an enhanced exploration and parameters for a smooth drift to exploitation operation. The generalized equation is given by

25 $$\begin{aligned} if \; rand< 0.5 \nonumber \\ if \; rand < 0.5 \nonumber \\ x_{i}^{t+1}=x_{i}^{t}+\mu _1(L.x_p^t-L.x_q^t) \nonumber \\ else \nonumber \\ x_{i}^{t+1}=x_{i}^{t}+\mu _2(L.x_p^t-L.x_q^t) \nonumber \\ end\nonumber \\ else \nonumber \\ x_{i}^{t+1}=x_{i}^{t}\nonumber \\ end \end{aligned}$$

where $\mu _1 = P \times R$ uses $P = (p_{max} - p_{min}).\times exp(1/(1+(2*t)/t_{max}))$ is exponentially decreasing as well as $R \in [0, 1]$, $\mu _2 = R \times CF$ utilize the same *P* as in $\mu _1$ whereas $CF = (1-t/t_{max})^(2 \times t/t_{max})$ is adaptive step size control parameter. The parameter *L* is taken from Lévy distribution and is given by $Levy\sim \mu =t^{-\beta },(1<\beta \le 3)$ and $\beta$ is the expected occurrence of events.

*d) Phase IV: iterations *> $t_{max} 3/4$: This is the last part of the worker phase. In this phase, we are concentrating on solutions that are better for both exploration as well as exploitation operations. More specifically, intensive exploration as well as extensive exploitation must be followed in this phase. We have used FuFiO for this specific part, this is because the algorithm has an intensive mechanism that follows both of these phenomena efficiently. The general equations of the FuFiO are adapted to make them a better fit for the current version of the algorithm. We have used equations of both stable and unstable nucleus phases of FuFiO and are formulated as

26 $$\begin{aligned} if abs(A) \ge 1 \nonumber \\ if p_a = = 1 \nonumber \\ x_i^{t+1} = x_i^t + g. (x_p^t - x_q^t) \nonumber \\ else \nonumber \\ x_i^{t+1} = x_i^t + (1-g). (x_p^t - x_q^t) \nonumber \\ end \nonumber \\ else \nonumber \\ if p_a = = 2\nonumber \\ x_i^{t+1} = x_i^t + g. (x_r^t) - x_s^t \nonumber \\ else \nonumber \\ x_i^{t+1} = x_i^t + (1-g). (x_r^t - x_s^t) \nonumber \\ end \nonumber \\ end \end{aligned}$$

where $p_a$ helps in the random selection either of the equations and is a random number either 1 or 2, $x_p, x_q, x_r, x_s$ are four random solutions that are independent of each other. The major reason for utilizing these equations is because of the intensive exploration due to randomization and extensive exploitation due to the more intensive Lévy flights mechanism.

#### Breeder phase

The subsequent stage of the proposed PIFN algorithm closely resembles that of the fundamental NMRA approach, with the inclusion of certain modifications. The only modification is added in the parametric settings of the algorithm, where $\lambda$ the parameter is enhanced by using certain parametric adaptations. More details about the adaptation on parameters are given in consecutive subsections. The main reason for keeping this phase as such is due to the added advantage of using the best and one random solution in the search space. This helps to search for potential solutions within the close vicinity of the current best solution and hence corresponds to better exploitation operations.

#### Selection operation

The selection operation is required to select new solutions from the pool of solutions. Every algorithm consists of a dedicated set of equations for this phase and is mainly meant to see which solution is better. Here, we compare the fitness of the previous best solution with respect to the current best. The best-fit solutions are kept, and the other is discarded. This process of selection is called as greedy selection mechanism and is presented by

27 $$\begin{aligned} x_i^{t+1}= {\left\{ \begin{array}{ll} x_i^t \hspace{20pt}if f(x_i^{t+1})<f(x_i^t) \\ x_i^{t+1} \hspace{35pt} otherwise \end{array}\right. } \end{aligned}$$

The next phase is the stagnation operation and is meant for local optima avoidance and is presented in the next subsection.

#### Stagnation phase

A common problem with most of the algorithms is the presence of local optima stagnation, and it becomes very difficult for the algorithm to avoid this problem. Here we add a new phase named as stagnation phase to overcome these problems. We introduce this phase by taking inspiration from SACS algorithm, and it uses a set of well-defined equations. Each of these formulated equations are given by

28 $$\begin{aligned} x_1=x_i-A_1(C_1.x_{new}-x_i^t);\hspace{5pt} x_2=x_i-A_2(C_2.x_{new}-x_i^t);\hspace{5pt} x_3=x_i-A_3(C_3.x_{new}-x_i^t) \end{aligned}$$

29 $$\begin{aligned} x_{i}^{t+1}=\frac{x_1+x_2+x_3}{3} \end{aligned}$$

where $A_1, A_2, A_3 \in A$ and $C_1,C_2,C_3 \in C$ are given by $A=2l.r_1-l;\hspace{5pt} C=2.r_2$. The equation (29) is adapted using Cauchy based randomization for better solution quality and the new equation is

30 $$\begin{aligned} x_i^{t+1}=x_i^t+\alpha \otimes {Cauchy(\delta )}(n_{best}-x_i^t) \end{aligned}$$

where $Cauchy(\delta )$ is obtained from Cauchy distribution and equations are given by

31 $$\begin{aligned} f_{Cauchy(0,G)}(\delta )=\frac{1}{\pi } \frac{G}{({G^2}+{\delta ^2})} \end{aligned}$$

The Cauchy distribution function is

32 $$\begin{aligned} y=\frac{1}{2}+\frac{1}{\pi }arctan(\frac{\delta }{G}) \end{aligned}$$

And $\delta$ is given by

33 $$\begin{aligned} \delta =tan(\pi (y-\frac{1}{2})) \end{aligned}$$

Above equations corresponds to the exploratory enhancements and this is due to the presence of fatty tailed Cauchy distribution. This further helps the algorithm come out of the local optima and hence prevents stagnation. Apart from that, all other notations are same as used in the algorithm and there is no modification in the basic notations of the algorithms.

### Parametric adaptations

The effect of the parameters of an optimization algorithm on its performance is widely recognized. Many parameters for the proposed algorithm must be optimized for improved performance. The exploration, as well as exploitation stages, are vital to every optimization technique. Therefore, a good balance between these two stages is necessary for the algorithm to perform effectively. The adaptive mutation operators are essential to achieving a balance between the exploration and exploitation stages^97^. The 5 distinct mutation operators (Simulated Annealing Inertia Weight (SAIW), Exponential Decreasing Inertia Weight (EDIW), Linear Decreasing Inertia Weight (LDIW), Oscillating Inertia Weight (OIW), and Chaotic Inertia Weight (CIW))^97^ employed for various parameters of the proposed algorithm are explained as follows:

#### Simulated annealing inertia weight

The SAIW’s mathematical equation is presented in Eq. (34):

34 $$\begin{aligned} A_k= A_{min}+(A_{max}-A{min})\times p^{(k-1)} \end{aligned}$$

where, $A_{min}$, $A_{max}$ and *k* uniformly distributed random numbers lying between [0,1] and value of *p* is considered as 0.95.

#### Exponential decreasing inertia weight

This inertia weight enables the algorithms to converge during the early phases of the search as well as solves the majority of continuous optimization challenges. This inertia mass’s mathematical model is written in Eq. (35).

35 $$\begin{aligned} A(iter)=A_{min}+(A_{max}-A_{min})exp\left[ -\frac{iter}{(\frac{t_{max}}{10})}\right] \end{aligned}$$

where ($A_{min}$ and $A_{max}$) is the minimum as well as maximum random values lie among [0,1].

#### Oscillating inertia weight

This OIW technique has been determined to be very competitive and improve the algorithm’s convergence rate. This Eq. (36) expressed the temporal behaviour of this inertia mass:

36 $$\begin{aligned} A(iter)=\frac{A_{min}+A_{max}}{2}+\frac{A_{max}-A{min}}{2}cos(\frac{2\pi \hspace{1.5pt}iter}{T}) \end{aligned}$$

37 $$\begin{aligned} T=\frac{2 \times t_{max}}{3+2k} \end{aligned}$$

where, the value of $A_{max}$ and $A_{min}$ is considered as 0.9 as well as 0.3. The (*k*) random number lies between [0,1], current iteration (*iter*), and the ($t_{max}$) maximum number of iterations that permitted the inertia weight to oscillate.

#### Linear decreasing inertia weight

With the assistance of this inertia weight, the algorithm can converge to the optimum global solution, despite the fact it might get trapped in the optimal local solution for very complex functions. The mathematical expression for this inertia mass is given in the following Eq. (38):

38 $$\begin{aligned} A_k=A_{max}-\left( \frac{A_{max}-A_{min}}{L_{max}} \right) \times k \end{aligned}$$

where, random numbers ($A_{max}$, $A_{min}$ and *k*) lies in the range of [0,1] and maximum number of iterations ($L_{max}$).

#### Chaotic inertia weight

This inertia weight improves the algorithm’s convergence rate as well as a global search capability. As following is the technique for chaotic inertia weight:

39 $$\begin{aligned} H=4 \times k \times (1-k) \end{aligned}$$

40 $$\begin{aligned} A= (A_{max}-A_{min}) \times \frac{t_{max}-iter}{L_{max}} + A_{min} \times H \end{aligned}$$

where random number (*k*) lies between [0,1], value of $A_{max}$ is defined to 0.9, value of $A_{min}$ is defined to 0.5, total number of iterations ($L_{max}$) and current iteration (*iter*).

Here, the above-indicated adaptive mutation operators are applied to the algorithm parameters listed in Table 1. With these operators’ aid, the algorithm’s performance can be analyzed.

**Table 1:** PIFN parameters which include distinct adaptive mutation operators.

Adaptive mutation operators	WOA	NMRA	MFO	MPA		
	*l*	$\lambda$	*g*	*CF*	*P*	*R*
EDIW (exp)	$\checkmark$	$\checkmark$	$\checkmark$	$\checkmark$	$\checkmark$	$\checkmark$
OIW (oscillating)	$\checkmark$		$\checkmark$			
SAIW (sa)		$\checkmark$				
LDIW (linear)	$\checkmark$		$\checkmark$			
CIW (chaotic)		$\checkmark$		$\checkmark$	$\checkmark$	$\checkmark$

The pseudocode for the PIFN algorithm is presented in Algorithm 1.

**Algorithm 1:**
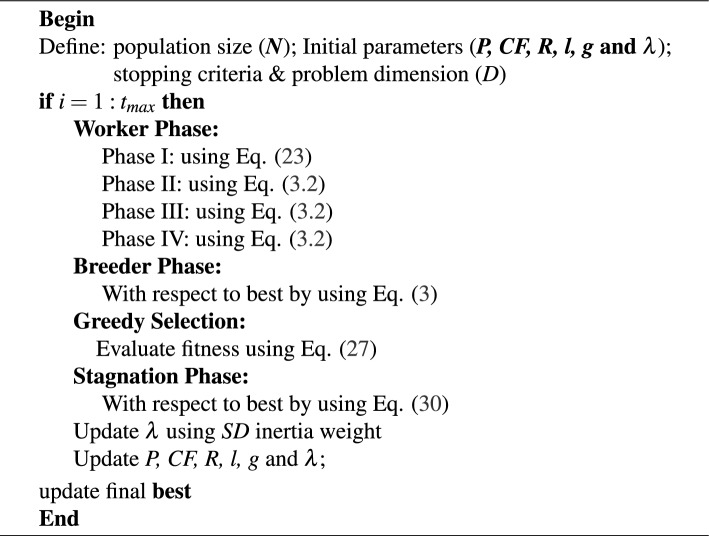
Pseudocode of the proposed PIFN algorithm

**Figure 2:**
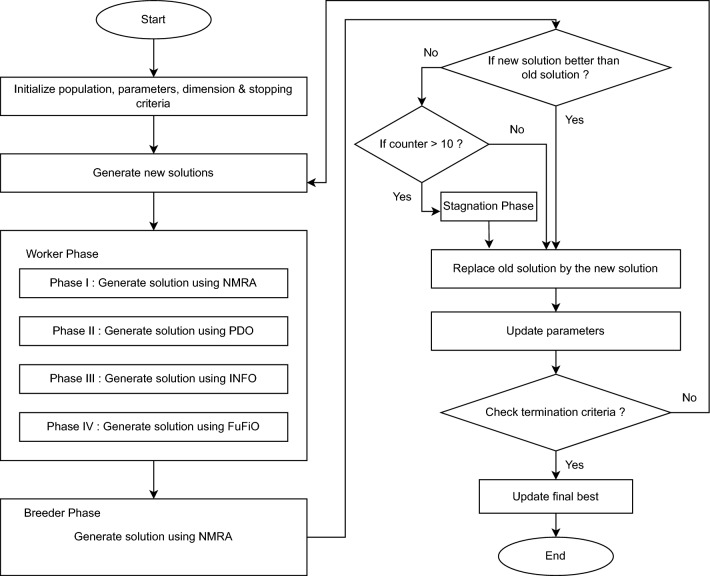
Flowchart of PIFN Algorithm.

The flowchart of the proposed algorithm is given in Fig. 2.

## Computational complexity

This section provides extensive details on the computational complexity of the proposed algorithm in terms of both runtime complexity and the space complexity (complexity represented as big-O notation).

### Runtime complexity

The experiments have been carried out utilizing Matlab 2022b on a 12th-generation Windows 11 operating system with a 64-bit architecture. The hardware specifications included an i5 processor, 16 GB of RAM, 18 MB of cache, 12 cores, 16 threads, and a system clock speed of 2.5 GHz. The given equation is utilized in order to calculate the run-time, denoted as $K1_0$^98^.

41 $$\begin{aligned} for \hspace{5pt} i = 1\,\,: \,\,nn_t; \\ Ka_{123} = 0.55;\,\, Ka_{123} = Ka_{123}+Ka_{123}; \,\, Ka_{123}= Ka_{123}/2; \,\, Ka_{123} = Ka_{123} \times Ka_{123};\,\, Ka_{123} = sqrt(Ka_{123}); \\ Ka_{123} = log(Ka_{123});\,\, Ka_{123} = exp(Ka_{123});\,\, Ka_{123} = Ka_{123}/(Ka_{123}+2);\\ end \end{aligned}$$

Where, the value of $nn_t$ is 1000000 for the classical benchmarks. The total number of iterations for the classical benchmark test suites is 2000. For a fair comparison, these values are the same for all the algorithms. The notations $Ko_1$ refer to the time taken for evaluating functions for one run. Also, $Ko_2$ refers to the times that took an average of five runs. The outcomes presented in Table 2 indicate that the PFIN algorithm exhibits a high level of competitiveness when compared to other algorithms. Furthermore, it demonstrates comparable complexities to the fundamental algorithms employed in the development of the PFIN algorithm.

**Table 2:** Analysis of time complexity.

	Function	Dim	Algorithm	$K1_0$	$K0_1$	$K0_2$	$(K0_2-K0_1)/K1_0$
Classical benchmark	1	10	PIFN	9.409E−03	1.023051E+00	9.36211E−01	9.2294611E+00
			PDOA	9.409E−03	1.93527E−01	8.5901E−02	1.143133298E+01
			INFO	9.409E−03	4.21659E−01	3.60093E−01	6.543309597E+00
			FuFiO	9.409E−03	8.17759E−01	7.61886E−01	5.938250611E+00
			NMRA	9.409E−03	1.91778E−01	1.43008E−01	5.18335E+00
			PIFN	9.409E−03	1.571058E+00	1.470129E+00	10.72685E+00
			PDOA	9.409E−03	4.78698E−01	419744E−01	6.265703E+00
			FuFiO	9.409E−03	5.11111E−01	4.74304E−01	3.911892869E+01
			INFO	9.409E−03	1.196532E+00	1.04342E+00	1.627293017E+01
			NMRA	9.409E−03	2.88721E−01	2.33143E−01	5.906897E+00

### Space complexity

The computational complexity of the fundamental NMRA is succinctly represented as O($n.d.t_{max}$). In this context, *n* symbolizes the size of the population, *d* denotes the dimensionality of the problem, as well as $t_{max}$ signifies the maximum iterations required to discover the global optimum. This complexity analysis aims to investigate how the algorithm behaves under the most challenging conditions and to estimate its runtime.

For each individual in the population, the complexity translates to O(*d*) due to the unchanging nature of the problem’s dimension. With the utilization of multiple search agents, the complexity expands to O(*n*.*d*) to encompass the population’s scale. Due to the algorithm’s stochastic nature, it’s necessary to evaluate it over $t_{max}$ iterations, resulting in an overall complexity of O($n.d.t_{max}$).

When comparing the original NMRA to the proposed PIFN algorithm, the exploration process is partitioned into multiple sets of iterations. However, it’s important to note that the total iteration count remains fixed at $t_{max}$, ensuring that this adaptation doesn’t alter the overall complexity. Concerning the newly introduced stagnation phase, the complexity aligns with O(*n*.*d*) and remains at 1. This is attributed to the stagnation phase being activated only when the algorithm remains unproductive for a specific number of iterations, leading to the generation of a new solution only once. Taking everything into account, the total complexity of the proposed PIFN algorithm remains equivalent to that of the basic NMRA.

## Result on benchmark problems

This section details about the results on the benchmark function used. Additionally, the section is split up into seven different subsections. The first subsection deals with the test suite used for comparison, second and third provides details on the basic parameters of the algorithms and an analysis of those parameters to see which set of parameters are the best. Fourth and fifth subsection details about the analysis of variable population and dimension sizes, whereas in the sixth seventh, and eighth subsection, a comparison with respect to classical benchmarks, CEC 2017, and CEC 2019 is performed. The results and analysis are performed on a MacBook PRO with an M2 processor using MATLAB R2022b. More details regarding the same is given in the following subsections.

### Test suite

The test suite’s details are presented in this subsection, in which the proposed algorithm PIFN’s performance is tested. The test functions are generally divided into three forms (unimodal (F1-F7), multimodal (F8-12), as well as fixed dimension (F13-F15)). The complete detail of the test functions is presented in Table  3.

**Table 3:** Classical Benchmark functions.

Function	$f_{min}$	Range	Dim	Shift position
*Unimodal*				
F1(k)= $\sum _{i=1}^{n} k_i^2$	0	$[-100,100]$	30	$[-30,-30,\ldots ,-30]$
F2(k)= $\sum _{i=1}^{n} |k_i|+\Pi _{i=1}^n |k_i|$	0	$[-10,10]$	30	$[-3,-3,\ldots ,-3]$
F3(k)= $\sum _{i=1}^{n}(\sum _{j-1}^i k_j)^2$	0	$[-100,100]$	30	$[-3,-3,\ldots ,-3]$
F4(k)= $max_i$ $\{$ $|k_i|, 1\le i \le n$ $\}$	0	$[-100,100]$	30	$[-3,-3,\ldots ,-3]$
F5(k)= $\sum _{i=1}^{n-1} 100(k_{i+1}-k_i^2)^2+(k_1-1)^2$	0	$[-30,30]$	30	$[-3,-3,\ldots ,-3]$
F6(k)= $\sum _{i=1}^{n}([k_i+0.5])^2$	0	$[-10,10]$	30	$[-3,-3,\ldots ,-3]$
F7(k)= $\sum _{i=1}^{n} ik_i^4 =random [0,1]$	0	$[-1.28,1.28]$	30	$[-3,-3,\ldots ,-3]$
*Multimodal*				
F8(k)= $\sum _{i=1}^{n} [k_i^2-10cos(2\pi k_i)+10]$	0	$[-5.12,5.12]$	30	$[-30,-30,\ldots ,-30]$
F9(k)= $-20exp(-0.2 \sqrt{\frac{1}{n}}\sum _{i=1}^n k_i^2)-exp(\frac{1}{n}\sum _{i=1}^n cos(2\pi k_i))+20+e$	0	$[-100,100]$	30	$[-30,-30,\ldots ,-30]$
F10(k)= $\frac{1}{4000}\sum _{i=1}^N k_i^2 - \Pi _{i=1}^N cos(\frac{k_i}{\sqrt{i}})+1$	0	$[-600,600]$	30	$[-30,-30,\ldots ,-30]$
F11(k)= $\frac{\pi }{n}10sin(\pi y_1)+\sum _{i=1}^n-1(y_i-1)^2[1+10sin^2(\pi y_{i+1})]$	0	$[-50,50]$	30	$[-30,-30,\ldots ,-30]$
$${(y_n-1)^2+\sum _{i=1}^u(k_i,10,100,4) y_i=1=\frac{k_i+1}{4}}$$				
F12(k)= $0.1 ( \sin ^2(3\pi k_1)+\sum _{i=1}^n(k_i-1)^2(1+\sin ^2(3\pi k_i+1)))$	0	$[-50,50]$	30	$[-30,-30,\ldots ,-30]$
$$+0.1((k_n-1)^2[1+\sin ^2(2\pi k_n])+\sum _{i=1}^n u(k_1, 5, 100, 4)$$				
*Fixed dimension*				
F13(k)= $[1+(k_1+k_2+1)^2(19-14k_1+3k_1^2-14k_2+6k_1k_2+3k_2^2)]*$	3	$[-2,2]$	2	
$$[30+(2k_1-3k_2)^2*(18-32k_1+12k_1^2+48k_2-36k_1k_2+27k_2^2)]$$				
F14(k)= $-\sum _{i=1}^4 c_i \exp (-\sum _{j=1}^3 a_{ij}(k_j-p_{ij})^2)$	$-3.86$	[1, 3]	3	
F15(k)= $-\sum _{i=1}^4 c_i \exp (-\sum _{j=1}^6 a_{ij}(k_j-p_{ij})^2)$	$-3.86$	[0, 1]	6	

### Parametric details

This section evaluates the effectiveness of the PIFN algorithm related to classical benchmarks, CEC 2017, as well as CEC 2019 benchmark challenges. First, for classical benchmark functions, the PIFN algorithm is tested as well as compared with multi-hybrid algorithm (MHA)^61^, SHADE^99^, SaDE^100^, Evolution strategy with covariance adaptation (CMA-ES)^66^, OEWOA^101^, LSHADE-SPACMA^65^, fractional-order calculus-based flower pollination algorithm (FA-FPO)^67^, Extended GWO (GWO- E)^68^, Sine Cosine CSA (SCCSA)^69^, equilibrium optimizer (EO)^70^, and JADE^62^. Second, for benchmark (CEC 2019) functions, the proposed PIFN algorithm is tested as well as compared with the SHADE^102^, SaDE^103^, JADE^103^, MVMO^103^, CV1.0^104^, $CV_{new}$^105^, and CS algorithms. Lastly, for benchmark (CEC 2019) functions, the proposed PIFN algorithm is tested as well as compared with the self-adaptive DE algorithm (jDE100)^63^ (winner algorithm of CEC 2019), DE^106^, Young’s double-slit experiment optimizer (YDSE)^64^, FPA^107^ and NMRA^52^. The parameter settings of every algorithm are obtained from their publications as well as tabulated in Table 4.

**Table 4:** Parametric specification of distinct algorithms.

Algorithm	Parameters
NMRA^52^	$\lambda =[0,1]$; $bp=0.5$;
JADE^62^	$p=[0.05,0.20]$; $CR = 0.9$; $F = 0.5$; $1/c =[5,20]$
OEWOA^101^	$b=1$; $\vec {\alpha }$ = Exponentially decreasing function;
HPFPPA-D^108^	$\sigma$=5; $N_{max}$=7
SaDE^62^	$F, CR=$ self adaptive
SHADE^70^	*ARC* *rate*= 2; $P_{best}=0.1$
LSHADE-SPACMA^70^	*c*=0.8, $P_{best}=0.11$, *ARC* *rate*= 1.4, *FCP*=0.5
EO^70^	$a_1=2$, $a_2=1$, $sign(r-0.5)$
GWO-E^109^	Linearly decreasing ($\vec {\alpha }$) from 2 to 0
GWO^110^	$\vec {\alpha }$ = Linearly decreasing [2,0]
SCCSA^111^	$r_1, r_2, r_3 = [0,1]$
FA-FPO^67^	$\alpha = [0.1,1]$, $S=adaptive$
MFO^112^	$t=[-1,1]$; $b=1$
YDSE^64^	rand=[0,1]; $\delta =0.38$
MHA^61^	$CF=adaptive$; $P=exp$; $\lambda$=*sa*; $bp=0.05$; Linearly decreasing (*l*) = [-1,-2]; $\vec{R}=[0,1]$; Linearly decreasing *t* = [2,0]
PPA^108^	$N_{max}$=7; $\sigma$=5
CMA-ES^66^	$c_c$ =1; $\alpha _{cov}$=1
PFA^108^	$\sigma$=5; $N_{max}$=7
WOA^113^	Linearly decreasing ($\vec {\alpha }$)=[2,0]; $b=1$;
PIFN	$\lambda$=*sa*; $bp=0.05$; $CF=exp$; $P=0.5$; Linearly decreasing *l* = [-1,-2]; $\vec{R}=[0,1]$; Linearly decreasing *t* = [2,0]

### Parameter analysis

The proposed PIFN algorithm is actually a hybridization of six different algorithms: PDOA, NMRA, FuFiOA, INFO, GWO, along with the CSA. Therefore, the essential parameters of these algorithms have been analyzed in this subsection. The first step is evaluating the parameter *CF* for three cases, including adaptive nature, chaotic inertia weight, and exp inertia weight. According to the outcomes in Table  5, the exp inertia weight of parameter CF can also provide improved outcomes for functions F4, F7 and F13. The parameter CF with chaotic inertia weight performs better than other cases for functions F11. The outcomes are only compared for mean values for the F12, where the parameter *CF* with the adaptive nature operator performs the best. The second step is evaluating the parameter P for three cases, including exp inertia weight, chaotic inertia weight and constant number. Table  5, the constant number of parameters *P* can also provide improved outcomes for functions F7, F12 and F13. The outcomes are only compared for mean values for the F4 and F11, where the parameter P with the chaotic inertia weight operator performs the best. The third step is evaluating the parameter R for three cases: random nature, exp inertia weight, and chaotic inertia weight. According to the outcomes in Table  5, the random nature of parameter *R* can also provide improved outcomes for functions F4, F7, F11 and F13. The parameter R with chaotic *iw* performs better than other cases for function F12. For the *l*, linear decreasing *iw* provides superior outcomes for the function, F7, F11, and F12. The parameter l with oscillating inertia weight performs better than other case for function F13. Also,for the parameters g with linear decreasing *iw* provides best outcomes for the function, F4, F7 and F13.

According to the results highlighted in Table  5, the simulated annealing *iw* of parameter $\lambda$ can also provide improved results for functions F4, F7,and F12. The parameter $\lambda$ with chaotic *iw* performs better than other cases for function F11.Thus, among all the other operators utilized for comparison, the overall parameters CF with exp inertia weight, P with the constant number, g with linear *iw*, l with linear *iw*, $\lambda$ with simulated annealing *iw* as well as the parameter R with random nature are found to be the superior. In order to validate the outcomes, an f-rank statistical test is carried out for each instance.

**Table 5:** Parameter analysis of PIFN.

Parameters	Variable		Functions					Statistical analysis		Best Strategy
			*F*4	*F*7	*F*11	*F*12	*F*13	Average f-rank value	Overall f-rank	
$\lambda$	$\lambda _{sa}$	mean	1.06E−180	9.24E−05	1.40E−02	1.68E−01	3.00E+00	1.6	1	$\lambda _{sa}$
		std	0.00E+00	9.17E−05	6.10E−03	7.14E−02	8.47E−08			
		f-rank	1	1	2	1	3			
	$\lambda _{chaotic}$	mean	1.68E−157	1.05E−04	1.30E−02	1.74E−01	3.00E+00	1.8	2	
		std	1.20E−156	1.04E−04	5.40E−03	6.74E−02	3.75E−12			
		f-rank	2	2	1	2	2			
	$\lambda _{exp}$	mean	2.23E−140	1.09E−04	1.50E−02	1.80E−01	3.00E+00	2.6	3	
		std	1.49E−139	9.74E−05	6.90E−03	8.98E−02	4.99E−13			
		f-rank	3	3	3	3	1			
*CF*	$CF_{adaptive}$	mean	5.02E−141	9.80E−05	1.770E−02	2.39E−01	3.00E+00	2.4	3	$CF_{exp}$
		std	1.76E−140	8.56E−05	9.90E−03	8.42E−02	2.14E−12			
		f-rank	3	2	2	1	3			
	$CF_{chaotic}$	mean	4.67E−141	1.19E−04	1.64E−02	2.56E−01	3.00E+00	2	2	
		std	3.03E−140	1.03E−04	7.30E−02	9.23E−02	1.85E−12			
		f-rank	2	3	1	2	2			
	$CF_{exp}$	mean	2.35E−141	6.96E−05	1.84E−02	2.84E−01	3.00E+00	1.8	1	
		std	1.08E−140	7.23E−05	9.10E−03	7.82E−02	4.25E−13			
		f-rank	1	1	3	3	1			
*P*	$P_{0.5}$	mean	2.35E−141	6.96E−05	1.64E−02	2.44E−01	3.00E+00	1.4	1	$P_{0.5}$
		std	1.08E−140	7.23E−05	7.30E−03	8.56E−02	4.25E−13			
		f-rank	2	1	2	1	1			
	$P_{chaotic}$	mean	7.92E−142	8.64E−05	1.56E−02	2.67E−01	3.00E+00	2	2	
		std	4.37E−141	6.96E−05	7.80E−03	6.53E−01	1.02E−11			
		f-rank	1	3	1	2	3			
	$P_{exp}$	mean	1.13E−140	8.75E−05	1.78E−02	2.75E−01	3.00E+00	2.6	3	
		std	1.9E−139	8.84E−05	8.10E−03	9.62E−02	9.91E−12			
		f-rank	3	2	3	3	2			
*R*	$R_{random}$	mean	1.54E−141	6.96E−05	1.56E−02	2.40E−01	3.00E+00	1.2	1	$R_{random}$
		std	3.92E−140	7.32E−05	7.80E−03	7.29E−02	8.15E−13			
		f-rank	1	1	1	2	1			
	$R_{chaotic}$	mean	2.79E−141	8.87E−05	1.58E−02	2.1700E−01	3.00E+00	1.8	2	
		std	1.272E−140	8.47E−05	6.80E−03	8.88E−02	3.96E−12			
		f-rank	2	2	2	1	2			
	$R_{exp}$	mean	1.94E−140	9.35E−05	1.63E−02	2.57E−01	3.00E+00	3	3	
		std	1.13e-139	8.56e-05	6.80E−03	8.77E−02	5.7986E−12			
		f-rank	3	3	3	3	3			
*l*	$l_{linear}$	mean	1.15E−141	6.96E−05	1.56E−02	1.96E−01	3.00E+00	1.4	1	$l_{linear}$
		std	6.12E−141	7.32E−05	7.80E−03	7.39E−02	1.18E−12			
		f-rank	2	1	1	1	2			
	$l_{oscillating}$	mean	3.84-141	9.19E−05	2.18E−02	2.40E−01	3.00E+00	2.2	2	
		std	1.95E−140	9.17E−05	1.03E−02	1.02E−01	1.16E−12			
		f-rank	3	2	3	2	1			
	$l_{exp}$	mean	2.73E−142	9.79E−05	1.89E−02	2.41E−01	3.00E+00	2.4	3	
		std	8.89E−142	9.44E−05	1.04E−02	7.80E−02	1.25E−12			
		f-rank	1	3	2	3	3			
*g*	$g_{linear}$	mean	2.73E−142	7.69E−05	1.56E−02	2.09E−01	3.00E+00	1.6	1	$g_{linear}$
		std	8.89E−142	7.42E−05	7.80E−03	7.63E−02	8.25E−13			
		f-rank	1	1	2	3	1			
	$g_{oscillating}$	mean	4.46E−141	1.63E−04	1.73E−02	1.82E−01	3.00E+00	2.4	3	
		std	2.30E−140	1.25E−04	8.2E−03	7.61E−02	7.63E−12			
		f-rank	2	3	3	1	3			
	$g_{exp}$	mean	1.89E−140	9.30E−05	1.49E−02	1.93E−01	3.00E+00	2	2	
		std	1.28E−139	7.62E−05	7.10E−03	7.48E−02	1.81E−12			
		f-rank	3	2	1	2	2			

### Population analysis

The impact of population size on the operation of WOA, MHA, NMRA, MFO, MPA, GWO, as well as the proposed PIFN, has been examined in this part. The 4 sets of population sizes of 25, 50, 75, as well as 100 are utilized to evaluate each of the algorithms under evaluation. The number of runs is 51 and the outcomes for the 500 iterations are shown as mean values along with standard deviation (Std) values in Table  6. In this case, twelve test functions from Table Y are utilized to examine the impact of population size on different methods under comparison. These functions include seven unimodal functions (F1-F7) but also five multimodal functions (F8-F12). The findings are discussed in depth under the following key subheadings.

#### Population size 25

In this context, for functions F1, F2, as well as F5, WOA offers results close to the global optimal solution, despite PIFN results being quite competitive. PIFN performs best for both mean and Std values for functions F3, F4, and F7. MPA is best for all other algorithms in the comparison for functions F6, F11, and F12. For functions F8, MPA, WOA, MHA, and PIFN may achieve a value of the global minimal solution. PIFN provides the optimum performance for function F9. MPA, NMRA, MHA, and PIFN are all capable of providing the best value for function F10. Hence, WOA performs best in four functions, NMRA in one function, MPA in five functions, MHA in two functions, and PIFN in six functions. Overall, PIFN is considered to be the most suitable choice for this population size.

#### Population size 50

MHA and PIFN are able to produce the optimum value for function F1. In this context, the outcomes of PIFN have been discovered to be superior to other methods for functions F2, F3, F4, F7, as well as F9. The PIFN performance is optimum for Std values for function F5. MPA performs optimally for both mean as well as Std. values for functions F6, F11, as well as F12. The NMRA, MPA, MHA, as well as the proposed PIFN all give the precise optimal value for functions F8 and F10. PIFN produces superior results for function F9 when compared to all other algorithms. It can be observed that PIFN performs superior for nine functions, NMRA for two functions, MPA for five functions, as well as MHA for three functions. As a result, this scenario demonstrates that PIFN is overall preferable for population sizes of 50.

#### Population size 75

PIFN produces the best results for functions F1, F3, F5, F8, F9, and F10 for this population size. WOA has the best outcome for function F2. However, PIFN is relatively close to it. When it comes to function F4, NMRA outperforms other methods. MPA produces the greatest results for functions F6, F11, and F12, while all other methods produce competitive outcomes. For function F8, the mean and Std values of MPA, MHA, and PIFN are all identically zero, making it impossible to choose the optimal one. PIFN may approach a global minimum solution for function F9. The NMRA, MPA, MHA, as well as PIFN all give the same as well as the best value for function F10. Thus, PIFN outperforms NMRA for two functions, MPA for five functions, WOA for one function, and MHA for three functions. Overall, PIFN is also determined to be the best in this scenario.

#### Population size 100

In this context, only MHA is capable of providing near-optimal results for functions F1, F3, F5, and F7 when compared to other methods. WOA has the most outstanding performance for function F2, whereas PIFN is relatively close to it. PIFN produces the best results for functions F4, F8, F9, and F10. PIFN and NMRA produce the best results for function F10. MPA is shown to be the best for functions F6, F11, and F12. Thus, PIFN is superior in four functions, WOA in one function, NMRA in one function, MPA in three functions, and MHA in four functions. As a result, PIFN is once again the most incredible option for this population size.

#### Interference from the population analysis

The outcomes show that too low population size values reduce the proposed algorithm’s performance, while too large population size values offer minimal importance while increasing the algorithm’s computational load. Here, an average population size of 50 promises to give incredibly reliable outcomes without compromising the algorithm’s computational complexity. Therefore, a population size of 50 is taken into account for the rest of the experimental analysis.

**Table 6:** Experimental outcomes for population size of 25, 50, 75, and 100.

Function	Algorithm	Popu Size 25		Popu Size 50		Popu Size 75		Popu Size 100	
		Mean	Std	Mean	Std	Mean	Std	Mean	Std
F1	MFO	1.80E+03	3.83E+03	1.17E+03	3.25E+03	5.89E+02	2.37E+03	9.81E+02	3.00E+03
	MHA	1.63E−59	1.16E−58	0.00E+00	0.00E+00	3.86E−109	2.53E−108	1.49E−108	1.00E−107
	WOA	1.62E−67	1.13E−66	1.11E−83	7.37E−83	6.41E−90	4.37E−89	1.19E−95	7.91E−95
	NMRA	1.12E−48	7.04E−48	1.12E−86	7.88E−86	5.80E−86	3.95E−85	6.03E−80	4.28E−79
	MPA	2.69E−23	3.48E−23	5.05E−23	4.93E−23	2.76E−23	3.37E−23	2.51E−23	2.55E−23
	GWO	1.38E−25	1.98E−25	3.14E−33	5.32E−33	8.63E−38	1.07E−37	8.77E−41	1.39E−40
	PIFN	1.49E−50	1.06E−49	0.00E+00	0.00E+00	2.47E−110	1.38E−109	8.74E−101	6.24E−100
F2	MFO	3.21E+01	1.87E+01	3.11E+01	2.00E+01	3.08E+01	2.20E+01	3.10E+01	2.26E+01
	MHA	2.87E−33	1.51E−32	1.63E−180	0.00E+00	8.78E−49	6.27E−48	1.29E−53	8.99E−53
	WOA	5.95E−49	3.58E−42	1.88E−54	5.69E−54	4.06E−56	1.48E−55	3.46E−57	1.21E−56
	NMRA	6.48E−25	3.78E−24	3.46E−45	1.52E−44	6.91E−44	2.24E−43	1.41E−42	9.11E−42
	MPA	2.24E−13	2.24E−13	3.08E−13	2.98E−13	2.89E−13	2.42E−13	2.26E−13	1.62E−13
	GWO	1.51E−15	1.22E−15	7.11E−20	6.53E−20	2.42E−22	2.01E−22	4.53E−24	4.02E−24
	PIFN	2.09E−34	1.49E−33	1.01E−183	0.00E+00	1.10E−45	7.04E−45	9.58E−55	6.76E−54
F3	MFO	2.65E+04	1.49E+04	1.89E+04	1.23E+04	1.51E+04	1.19E+04	1.35E+04	9.34E+03
	MHA	2.89E−87	2.06E−86	1.16E−318	0.00E+00	6.30E−141	4.50E−140	3.28E−114	2.34E−113
	WOA	5.10E+04	1.41E+04	2.97E+04	9.33E+03	1.98E+04	6.95E+03	1.62E+04	7.91E+03
	NMRA	2.34E−47	1.61E−46	2.54E−85	1.32E−84	3.34E−83	1.76E−82	1.48E−83	8.16E−83
	MPA	8.66E−05	1.37E−04	6.71E−05	1.37E−04	1.69E−05	3.78E−05	7.97E−06	1.88E−05
	GWO	8.55E−05	1.95E−04	3.85E−08	6.91E−08	2.20E−10	4.15E−10	1.07E−11	2.40E−11
	PIFN	3.58E−135	2.55E−134	8.40E−323	0.00E+00	6.75E−143	4.75E−142	2.32E−105	2.37E−104
F4	MFO	6.98E+01	1.00E+01	6.03E+01	9.31E+00	4.63E+01	1.12E+01	4.29E+01	9.34E+00
	MHA	3.98E−36	2.84E−35	1.00E−185	0.00E+00	1.52E−24	9.18E−24	6.34E−25	2.04E−24
	WOA	5.23E+01	2.56E+01	3.72E+01	2.87E+01	3.38E+01	2.91E+01	2.92E+01	2.73E+01
	NMRA	8.99E−26	3.85E−25	3.50E−45	1.52E−44	6.57E−43	2.73E−42	9.12E−43	3.27E−42
	MPA	3.16E−09	2.13E−09	3.15E−09	1.75E−09	2.21E−09	1.14E−09	1.82E−09	8.54E−10
	GWO	2.32E−06	2.40E−06	2.18E−08	1.74E−08	1.30E−09	1.14E−09	1.95E−10	1.74E−10
	PIFN	9.94E−45	7.10E−44	5.96E−186	0.00E+00	7.66E−25	3.19E−24	2.47E−25	1.24E−24
F5	MFO	1.58E+06	1.12E+07	1.32E+04	3.11E+04	1.49E+04	3.28E+04	1.16E+04	2.90E+04
	MHA	2.86E+01	1.27E−01	2.84E+01	3.23E−01	2.83E+01	3.43E−01	2.80E+01	4.47E−01
	WOA	2.10E+01	4.63E−01	2.74E+01	4.79E−01	2.70E+01	2.92E−01	2.68E+01	1.90E−01
	NMRA	2.89E+01	1.92E−02	2.89E+01	2.54E−02	2.89E+01	2.49E−02	2.89E+01	2.71E−02
	MPA	2.54E+01	4.36E−01	2.45E+01	4.37E−01	2.40E+01	4.54E−01	2.35E+01	3.59E−01
	GWO	2.70E+01	7.14E−01	2.67E+01	6.86E−01	2.65E+01	6.58E−01	2.63E+01	6.47E−01
	PIFN	2.87E+01	5.39E−02	2.86E+01	4.94E−02	2.86E+01	4.16E−02	2.86E+01	1.13E−01
F6	MFO	2.36E+03	5.85E+03	5.92E+02	2.38E+03	1.16E+03	3.23E+03	3.93E+02	1.96E+03
	MHA	5.64E−01	1.90E−01	3.86E−01	1.28E−01	3.04E−01	9.75E−02	2.35E−01	9.91E−02
	WOA	6.18E−01	3.03E−01	8.45E−02	1.20E−01	2.08E−02	3.18E−02	5.60E−03	2.70E−03
	NMRA	6.84E+00	4.91E−01	6.56E+00	5.90E−01	6.62E+00	6.44E−01	6.53E+00	6.44E−01
	MPA	2.60E−03	1.27E−02	1.43E−08	6.25E−09	7.23E−09	2.73E−09	4.18E−09	1.91E−09
	GWO	9.70E−01	4.02E−01	4.70E−01	2.77E−01	2.82E−01	2.40E−01	1.80E−01	1.98E−01
	PIFN	6.70E−01	2.71E−01	4.39E−01	1.29E−01	3.39E−01	1.60E−01	3.10E−01	1.00E−01
F7	MFO	6.34E+00	1.10E+01	3.02E+00	7.37E+00	9.18E−01	2.17E+00	1.86E+00	5.82E+00
	MHA	2.27E−04	2.55E−04	9.65E−05	1.19E−04	7.62E−05	8.52E−05	6.88E−05	6.90E−05
	WOA	3.90E−03	4.90E−03	2.30E−03	2.70E−03	1.20E−03	1.30E−03	1.30E−03	1.40E−03
	NMRA	2.30E−03	2.40E−03	6.94E−04	6.10E−04	5.90E−04	5.52E−04	4.03E−04	3.26E−04
	MPA	1.40E−03	8.01E−04	1.00E−03	4.32E−04	8.40E−04	4.50E−04	8.35E−04	4.01E−04
	GWO	2.30E−03	1.30E−03	1.20E−03	5.08E−04	8.61E−04	4.49E−04	6.12E−04	4.07E−04
	PIFN	1.51E−04	1.50E−04	9.20E−05	8.77E−05	8.85E−05	1.06E−04	8.59E−05	1.08E−04
F8	MFO	1.69E+02	4.03E+01	1.52E+02	3.04E+01	1.34E+02	3.36E+01	1.43E+02	4.09E+01
	MHA	0.00E+00	0.00E+00	0.00E+00	0.00E+00	0.00E+00	0.00E+00	0.00E+00	0.00E+00
	WOA	2.23E−15	1.59E−14	1.11E−15	7.95E−15	1.11E−15	7.95E−15	4.45E−15	1.91E−14
	NMRA	2.43E+00	1.74E+01	0.00E+00	0.00E+00	2.36E+00	1.68E+01	6.42E+00	3.21E+01
	MPA	0.00E+00	0.00E+00	0.00E+00	0.00E+00	0.00E+00	0.00E+00	0.00E+00	0.00E+00
	GWO	5.05E+00	5.84E+00	1.64E+00	3.06E+00	2.28E+00	3.84E+00	7.12E−01	1.93E+00
	PIFN	0.00E+00	0.00E+00	0.00E+00	0.00E+00	0.00E+00	0.00E+00	0.00E+00	0.00E+00
F9	MFO	1.58E+01	5.95E+00	1.16E+01	8.60E+00	1.02E+01	9.37E+00	9.54E+00	8.81E+00
	MHA	8.88E−16	0.00E+00	8.88E−16	0.00E+00	8.88E−16	0.00E+00	8.88E−16	0.00E+00
	WOA	4.99E−15	2.96E−15	4.44E−15	2.24E−15	4.72E−15	2.54E−15	4.09E−15	2.77E−15
	NMRA	8.88E−16	0.00E+00	8.88E−16	0.00E+00	8.88E−16	0.00E+00	8.88E−16	0.00E+00
	MPA	1.71E−12	1.13E−12	1.44E−12	8.94E−13	1.22E−12	7.14E−13	1.26E−12	6.00E−13
	GWO	2.04E−13	5.47E−13	4.26E−14	3.32E−14	3.18E−14	4.33E−15	2.82E−14	2.86E−15
	PIFN	4.44E−16	0.00E+00	4.44E−16	0.00E+00	4.44E−16	0.00E+00	4.44E−16	0.00E+00
F10	MFO	3.33E−02	7.54E−02	5.70E−03	2.85E−02	2.90E−03	2.04E−02	0.00E+00	0.00E+00
	MHA	0.00E+00	0.00E+00	0.00E+00	0.00E+00	0.00E+00	0.00E+00	0.00E+00	0.00E+00
	WOA	3.43E−02	6.24E−02	4.28E−02	6.70E−02	1.71E−02	4.74E−02	1.43E−02	4.37E−02
	NMRA	0.00E+00	0.00E+00	0.00E+00	0.00E+00	0.00E+00	0.00E+00	0.00E+00	0.00E+00
	MPA	0.00E+00	0.00E+00	0.00E+00	0.00E+00	0.00E+00	0.00E+00	0.00E+00	0.00E+00
	GWO	8.60E−03	3.46E−02	8.60E−03	3.46E−02	0.00E+00	0.00E+00	0.00E+00	0.00E+00
	PIFN	0.00E+00	0.00E+00	0.00E+00	0.00E+00	0.00E+00	0.00E+00	0.00E+00	0.00E+00
F11	MFO	7.20E+04	5.05E+05	5.32E+00	6.64E+00	2.47E+00	1.52E+00	2.51E+00	1.82E+00
	MHA	2.62E−02	1.58E−02	1.65E−02	8.20E−03	1.11E−02	4.20E−03	1.01E−02	4.20E−03
	WOA	3.36E−02	2.13E−02	7.40E−03	6.30E−03	2.60E−03	4.30E−03	1.90E−03	7.20E−03
	NMRA	1.03E+00	2.54E−01	1.09E+00	2.56E−01	1.04E+00	2.49E−01	1.10E+00	2.31E−01
	MPA	2.43E−04	8.85E−04	1.39E−09	6.50E−10	6.19E−10	3.34E−10	4.21E−10	1.76E−10
	GWO	5.84E−02	3.22E−02	2.59E−02	1.31E−02	1.79E−02	1.16E−02	1.55E−02	8.70E−03
	PIFN	2.60E−02	1.23E−02	1.82E−02	8.0E−03	1.48E−02	8.70E−03	1.17E−02	6.10E−03
F12	MFO	8.04E+06	5.74E+07	8.02E+00	7.71E+00	4.84E+00	6.76E+00	3.75E+00	3.08E+00
	MHA	3.63E−01	1.46E−01	2.69E−01	8.12E−02	2.21E−01	1.06E−01	2.07E−01	7.86E−02
	WOA	7.44E−01	2.73E−01	2.25E−01	1.60E−01	7.28E−02	7.06E−02	4.32E−02	7.21E−02
	NMRA	2.08E+00	3.65E−01	2.97E+01	1.38E−01	2.98E+00	1.10E−02	3.02E+00	1.18E−01
	MPA	3.00E−02	3.57E−02	6.53E−04	2.60E−03	8.61E−04	3.00E−03	4.59E−04	2.20E−03
	GWO	7.39E−01	2.36E−01	3.34E−01	2.02E−01	2.37E−01	1.86E−01	1.87E−01	1.40E−01
	PIFN	3.34E−01	1.41E−01	2.69E−01	1.19E−01	2.05E−01	1.01E−01	1.82E−01	9.30E−02

### Dimension analysis

In this subsection, 12 classical test functions (F1-F12) are utilized to analyze the impact of dimension size on the performance of the proposed PIFN algorithm and other MH optimization algorithms (MHA, NMRA, MPA, WOA, GWO, and MFO). Seven unimodal functions (F1-F7) and five multimodal functions (F8-F12) are used from Table  7 for the performance assessment of these techniques. This analysis uses four dimensions (10, 30, 50, and 100). The outcomes are presented as the mean along with std values across 51 runs, 500 iterations for all algorithms are listed in Table 2. The outcomes of all the algorithms with different dimension sizes are addressed under the following subsections:

#### Dimension size 10

For dimension size 10, PIFN produces the best outcome values for mean and std for functions F1, F2, F3, F4, F7, F8, F9 and F10. The outcomes of PIFN can approach towards the optimal solution for functions F5, F6, F11, and F12, but the outcomes of MHA are much more competitive to them. The value of the function F10 is exactly the same as the optimum value for PIFN, MHA, MPA, and NMRA. Hence PIFN gives the better outcomes for eight functions (F1, F2, F3, F4, F7, F8, F9, and F10), one function for MFO (F6) and NMRA (F10), MHA for four functions (F1, F4, F8, and F10), and four function for MPA (F5, F10, F11, and F12).

#### Dimension size 30

For the dimension size 30 case, for functions F1, F2, F3, F7, F8, F9 and F10, the PIFN attains the best outcomes values for mean and standard deviation. The outcomes of PIFN can approach towards the optimal solution for functions F4, F5, F6, F11, and F12, but the outcomes of MHA are much more competitive to them. Four optimization techniques, PIFN, MPA, NMRA, and MHA, produce comparable and optimal solutions for functions F8 and F10. Hence PIFN gives the better outcomes for seven functions (F1, F2, F3, F7, F8, F9 and F10), two function for NMRA (F8, and F10), MHA for four functions (F1, F4, F8, and F10), and six function for MPA (F5, F6, F8, F10, F11, and F12). Overall, it is concluded that PIFN is superior for dimension size 30.

#### Dimension size 50

For functions F1, F2, F3, F4, F7, F8, F9, and F10, attain the best outcomes value for the mean and standard deviation by using the PIFN algorithm. Five optimization techniques, PIFN, MPA, NMRA, WOA and MHA, produce comparable and optimal solutions for function F8 and four optimization techniques, PIFN, MPA, NMRA, and MHA, have identical and optimal solutions for function F10. The outcomes of PIFN can approach towards the optimal solution for functions (F5, F6, F11, and F12), but the outcomes of MHA are much more competitive to them. Hence PIFN offers the better outcomes for eight functions (F1, F2, F3, F4, F7, F8, F9, and F10), one function for WOA (F8), NMRA for two functions (F8 and F10), three functions for MHA (F1, F8, and F10), and MPA for five functions (F5, F6, F10, F11, and F12). Overall, it can be observed that PIFN is preferable for dimension size 50.

#### Dimension size 100

For functions F1, F2, F4, F5, F8, F9, F10, and F12, the performance of the PIFN is shown to be best compared with others. For function F11, PIFN provides the best results only for std values. Five optimization techniques, PIFN, MPA, NMRA, WOA and MHA, produces comparable and optimal solutions for function F8. Four optimization techniques, PIFN, MPA, NMRA, and MHA, have identical and optimal solutions for function F10, along with two optimizations, PIFN and MHA, which produce comparable and optimal solutions for function F1. The outcomes of PIFN can approach towards the optimal solution for functions (F3, F6, F7, and F11), but the outcomes of MHA are much more competitive to them. So that, PIFN offers the best outcomes for all the test functions, WOA for one function (F8), MHA for four functions (F3, F6, F7, as well as F11), two functions for MPA as well as MHA (F8 and F10). Overall, it is concluded that PIFN is superior between all the algorithms for dimension size 100.

#### Inference

The experimental outcomes demonstrate that the proposed PIFN algorithm shows superior performance with other algorithms under consideration for all dimension sizes. The dimension size of 50 is taken into account for the further performance of PIFN.

**Table 7:** Experimental outcomes for 10, 30, 50, and 100 dimension sizes.

Function	Algorithm	Dime Size 10		Dime Size 30		Dime Size 50		Dime Size 100	
		Mean	Std	Mean	Std	Mean	Std	Mean	Std
F1	MFO	5.27E−14	8.78E−14	1.17E+03	3.25E+03	5.72E+03	7.25E+03	5.39E+04	1.34E+04
	MHA	0.00E+00	0.00E+00	0.00E+00	0.00E+00	0.00E+00	0.00E+00	0.00E+00	0.00E+00
	WOA	4.34E−88	2.75E−87	1.11E−83	7.37E−83	5.86E−83	3.33E−82	2.66E−82	1.19E−81
	NMRA	3.00E−81	2.14E−80	1.12E−86	7.88E−86	4.33E−88	2.37e-87	6.66E−86	3.35E−85
	MPA	8.04E−31	1.36E−30	5.05E−23	4.93E−23	3.65E−21	3.04e-21	3.71E−19	3.62E−19
	GWO	6.91E−69	4.00E−68	3.14E−33	5.32E−32	9.66E−24	1.00E−23	5.28E−15	4.79E−15
	PIFN	0.00E+00	0.00E+00	0.00E+00	0.00E+00	0.00E+00	0.00E+00	0.00E+00	0.00E+00
F2	MFO	7.84E−01	2.71E+00	3.11E+01	2.00E+01	6.60E+01	3.00E+01	2.35E+02	4.19E+01
	MHA	1.01E−181	0.00E+00	1.63E−180	0.00E+00	9.78E−183	0.00E+00	9.69E−186	0.00E+00
	WOA	5.24E−55	2.06E−54	1.88E−54	5.69E−54	2.55E−52	1.59E−51	4.21E−54	9.56E−54
	NMRA	1.83E−44	9.36E−44	3.46E−45	1.52E−44	4.95E−44	3.37E−43	8.73E−45	2.77E−44
	MPA	7.86E−18	8.91E−18	3.08E−13	2.98E−13	3.04E−12	2.63E−12	2.44E−11	2.72E−11
	GWO	4.07E−40	6.81E−40	7.11E−20	6.53E−20	1.37E−14	7.65E−15	1.55E−09	5.47E−10
	PIFN	4.82E−187	0.00E+00	6.21E−188	0.00E+00	3.09E−188	0.00E+00	2.05E−186	0.00E+00
F3	MFO	9.80E+01	7.00E+02	1.89E+04	1.23E+04	5.82E+04	2.07E+04	2.23E+05	5.70E+04
	MHA	0.00E+00	0.00E+00	1.16E−318	0.00E+00	8.22E−310	0.00E+00	3.30E−289	0.00E+00
	WOA	4.18E+01	8.75E+01	2.97E+04	9.33E+03	1.55E+05	2.52E+04	9.00E+05	1.85E+05
	NMRA	6.40E−89	2.87E−88	2.54E−85	1.32E−84	9.70E−87	6.85E−86	1.99E−87	9.35E−87
	MPA	9.37E−15	1.55E−14	6.71E−05	1.37E−04	2.43E−02	4.17E−02	5.58E+00	6.67E+00
	GWO	1.27E−31	7.38E−31	3.85E−08	6.91E−08	3.40E−02	1.94E−01	1.46E+02	2.54E+02
	PIFN	0.00E+00	0.00E+00	0.00E+00	0.00E+00	9.88E−314	0.00E+00	1.70E−272	0.00E+00
F4	MFO	6.36E−02	3.06E−01	6.03E+01	9.31E+00	7.92E+01	5.76E+00	9.14E+01	2.41E+00
	MHA	7.83E−186	0.00E+00	1.00E−185	0.00E+00	3.53E−184	0.00E+00	2.04E−181	0.00E+00
	WOA	1.55E+00	4.46E+00	3.72E+01	2.87E+01	6.31E+01	2.95E+01	7.14E+01	2.81E+01
	NMRA	4.34E−45	2.66E−44	3.50E−45	1.52E−44	9.45E−42	6.75E−41	6.47E−45	3.16E−44
	MPA	6.70E−13	4.56E−13	3.15E−09	1.75E−09	2.58E−08	1.06E−08	2.76E−07	1.17E−07
	GWO	1.71E−22	3.09E−22	2.18E−08	1.74E−08	3.58E−05	2.88E−05	1.98E−01	2.33E−01
	PIFN	2.26E−184	0.00E+00	1.32E−185	0.00E+00	4.81E−186	0.00E+00	1.00E−183	0.00E+00
F5	MFO	5.73E+03	2.13E+04	1.32E+04	3.11E+04	3.52E+06	1.56E+07	1.23E+08	5.66E+07
	MHA	7.73E+00	4.40E−01	2.84E+01	3.23E−01	4.84E+01	1.36E−01	9.80E+01	9.84E−02
	WOA	6.29E+00	4.85E−01	2.74E+01	4.79E−01	4.77E+01	4.25E−01	9.78E+01	2.97E−01
	NMRA	8.97E+00	2.04E−02	2.89E+01	2.54E−02	4.89E+01	2.00E−02	9.89E+01	1.87E−02
	MPA	1.01E+00	3.05E−01	2.45E+01	4.37E−01	4.53E+01	3.50E−01	9.62E+01	7.83E−01
	GWO	6.35E+00	7.06E−01	2.67E+01	6.86E−01	4.72E+01	8.32E−01	9.74E+01	8.54E−01
	PIFN	8.61E+00	2.75E−01	2.86E+01	5.42E−02	4.85E+01	3.55E−02	9.80E+01	1.76E−02
F6	MFO	1.50E−13	4.25E−13	5.92E+02	2.38E+03	7.40E+03	8.69E+03	5.44E+04	1.40E+04
	MHA	3.77E−02	2.64E−02	3.86E−01	1.28E−01	7.81E−01	1.95E−01	1.74E+00	4.51E−01
	WOA	1.25E−04	9.45E−05	8.45E−02	1.20E−01	3.57E−01	1.56E−01	1.89E+00	6.07E−01
	NMRA	1.29E+00	7.47E−01	6.56E+00	5.90E−01	1.17E+01	4.84E−01	2.43E+01	4.66E−01
	MPA	7.02E−12	5.54E−12	1.43E−08	6.25E−09	2.26E−02	5.26E−02	2.16E+00	6.13E−01
	GWO	2.57E−06	9.73E−07	4.70E−01	2.77E−01	1.83E+00	6.35E−01	8.09E+00	9.96E−01
	PIFN	5.29E−02	3.29E−02	4.28E−01	1.52E−01	7.62E−01	2.26E−01	1.76E+00	4.77E−01
F7	MFO	5.50E−03	3.00E−03	3.02E+00	7.37E+00	1.92E+01	2.53E+01	2.45E+02	1.36E+02
	MHA	8.86E−05	1.05E−04	9.65E−05	1.19E−04	1.14E−04	1.19E−04	8.45E−05	9.11E−05
	WOA	1.60E−03	2.10E−03	2.30E−03	2.70E−03	2.80E−03	3.40E−03	2.80E−03	3.40E−03
	NMRA	8.18E−04	7.68E−04	6.94E−04	6.10E−04	6.44E−04	6.52E−04	6.65E−04	5.16E−04
	MPA	5.92E−04	3.66E−04	1.00E−03	4.32E−04	1.10E−03	3.72E−04	1.40E−03	6.73E−04
	GWO	4.68E−04	3.23E−04	1.20E−03	5.08E−04	2.20E−03	9.95E−04	4.70E−03	1.50E−03
	PIFN	8.56E−05	6.34E−05	7.59E−05	6.70E−05	1.12E−04	1.03E−04	1.09E−04	1.46E−04
F8	MFO	1.98E+01	1.14E+01	1.52E+02	3.04E+01	2.92E+02	5.23E+01	8.19E+02	7.31E+01
	MHA	0.00E+00	0.00E+00	0.00E+00	0.00E+00	0.00E+00	0.00E+00	0.00E+00	0.00E+00
	WOA	7.49E−01	5.35E+00	1.11E−15	7.95E−15	0.00E+00	0.00E+00	0.00E+00	0.00E+00
	NMRA	1.38E+00	5.72E+00	0.00E+00	0.00E+00	0.00E+00	0.00E+00	0.00E+00	0.00E+00
	MPA	3.16E−11	2.26E−10	0.00E+00	0.00E+00	0.00E+00	0.00E+00	0.00E+00	0.00E+00
	GWO	2.90E−01	1.07E+00	1.64E+00	3.06E+00	3.63E+00	4.62E+00	7.33E+00	5.93E+00
	PIFN	0.00E+00	0.00E+00	0.00E+00	0.00E+00	0.00E+00	0.00E+00	0.00E+00	0.00E+00
F9	MFO	6.88E−08	6.01E−08	1.16E+01	8.60E+00	1.90E+01	1.69E+00	1.98E+01	1.26E−01
	MHA	8.88E−16	0.00E+00	8.88E−16	0.00E+00	8.88E−16	0.00E+00	8.88E−16	0.00E+00
	WOA	4.02E−15	2.31E−15	4.44E−15	2.24E−15	4.09E−15	2.48E−15	5.06E−15	2.21E−15
	NMRA	8.88E−16	0.00E+00	8.88E−16	0.00E+00	8.88E−16	0.00E+00	8.88E−16	0.00E+00
	MPA	4.51E−15	4.97E−15	1.44E−12	8.94E−13	9.61E−12	4.79E−12	6.30E−11	2.97E−11
	GWO	5.83E−15	1.75E−15	4.26E−14	3.32E−15	5.32E−13	1.33E−13	7.09E−09	2.74E−09
	PIFN	4.44E−16	0.00E+00	4.44E−16	0.00E+00	4.44E−16	0.00E+00	4.44E−16	0.00E+00
F10	MFO	1.14E−02	3.95E−02	5.70E−03	2.85E−02	2.90E−03	2.04E−02	1.14E−02	3.95E−02
	MHA	0.00E+00	0.00E+00	0.00E+00	0.00E+00	0.00E+00	0.00E+00	0.00E+00	0.00E+00
	WOA	3.43E−02	6.24E−02	4.28E−02	6.70E−02	2.28E−02	5.35E−02	3.14E−02	6.05E−02
	NMRA	0.00E+00	0.00E+00	0.00E+00	0.00E+00	0.00E+00	0.00E+00	0.00E+00	0.00E+00
	MPA	0.00E+00	0.00E+00	0.00E+00	0.00E+00	0.00E+00	0.00E+00	0.00E+00	0.00E+00
	GWO	0.00E+00	0.00E+00	8.60E−03	3.46E−02	5.70E−03	2.85E−02	0.00E+00	0.00E+00
	PIFN	0.00E+00	0.00E+00	0.00E+00	0.00E+00	0.00E+00	0.00E+00	0.00E+00	0.00E+00
F11	MFO	1.70E−01	4.62E−01	5.32E+00	6.64E+00	1.53E+07	6.07E+07	2.29E+08	1.70E+08
	MHA	7.90E−03	5.00E−03	1.65E−02	8.20E−03	1.88E−02	7.90E−03	2.20E−02	1.04E−02
	WOA	2.40E−03	6.00E−03	7.40E−03	6.30E−03	1.14E−02	5.70E−03	1.81E−02	1.19E−02
	NMRA	8.24E−01	4.01E−01	1.09E+00	2.56E−01	1.16E+00	1.44E−01	1.20E+00	7.58E−02
	MPA	3.44E−12	3.00E−12	1.39E−09	6.50E−10	1.30E−03	1.40E−03	2.63E−02	7.20E−03
	GWO	1.80E−03	5.60E−03	2.59E−02	1.31E−02	8.64E−02	6.70E−02	2.10E−01	5.81E−02
	PIFN	1.03E−02	9.30E−03	1.71E−02	8.30E−03	1.86E−02	9.10E−03	1.90E−02	7.70E−03
F12	MFO	2.60E−03	4.70E−03	8.02E+00	7.71E+00	1.68E+07	8.02E+07	4.62E+08	2.80E+08
	MHA	5.58E−02	3.91E−02	2.69E−01	8.12E−02	4.26E−01	1.30E−01	9.82E−01	2.62E−01
	WOA	5.30E−03	8.80E−03	2.25E−01	1.60E−01	4.91E−01	2.33E−01	1.61E+00	6.87E−01
	NMRA	8.63E−01	1.76E−01	2.97E+00	1.38E−01	4.99E+00	2.50E−03	9.99E+00	2.00E−03
	MPA	1.75E−11	1.79E−11	6.53E−04	2.60E−03	8.58E−02	7.24E−02	5.62E+00	2.81E+00
	GWO	1.90E−03	1.36E−02	3.34E−01	2.02E−01	1.58E+00	3.38E−01	5.99E+00	4.46E−01
	PIFN	6.43E−02	3.12E−02	2.53E−01	1.19E−01	4.31E−01	1.36E−01	9.68E−01	3.10E−01

### Performance comparison on classical benchmark functions

The proposed PIFN performance is evaluated by comparison with other MH optimization techniques presented in this section. The MHA^61^, SHADE^99^, SaDE^100^, CMA-ES^66^, OEWOA^101^, LSHADE-SPACMA^65^, FA-FPO^67^, GWO- E^68^, SCCSA^69^, EO^70^, and JADE^62^. techniques are used for comparison. In the present work, all of these optimization techniques are assessed using benchmark (classical) functions with a dimension size of 30.

#### Experimental results

Table  8 illustrates the simulation outcomes for the algorithm’s 51 iterations and 30-dimension size in terms of mean and Std values. This table displays that only PIFN can achieve outcomes near the global optimal solution for functions F2, F3, F7, F9, and F10 whereas the other algorithms may be stuck in local minima. For function, F1, MHA, and PIFN offer good as well as similar outcomes, so it is problematic to comment on which one is good. Also, the function F8, PIFN, MHA, EO, OE−WOA, GWO-E, and FA-FPO offer the best and similar results. For the F11, F12, F13, and F14 functions, JADE is superior to other algorithms. For the F15 function, the FA-FPO is superior in terms of std. Hence from the 15 test functions, PIFN is found superior in 7 functions, JADE for 5 functions, MHA for 4 functions, 2 functions for EO and GWO-E, 4 functions for FA-FPO, and 1 function for CMA-ES. Overall, it is concluded that the performance of PIFN is superior than that of all other algorithms.

#### Statistical analysis

Two non-parametric tests, Wilcoxon’s and Friedman’s rank-sum test are used to check the statistical performance of the PIFN. When comparing the performance of two algorithms, the rank-sum test is utilized to assign the p-rank. In Wilcoxon’s rank-sum test, the statistical effectiveness of the proposed PIFN is demonstrated in contrast to other algorithms that have been utilized for comparison. When the proposed algorithm is compared with all competing algorithms, three scenarios will occur (loss(l)/win(w)/ tie(t)). First, the loss condition arises when the algorithm’s performance is the worst compared to the proposed algorithm and assigns a “-” sign. The win condition occurs when the algorithm’s performance is superior to the proposed algorithm and assigns a “+” sign. And the last case is the tie condition, which occurs when both the algorithm’s performance is significant to the proposed algorithm and assigns the “=”sign. So, l/w/ t values are presented in Table  8. The statistical test known as the Friedman rank (f-rank) assigns f-rank to each optimization algorithm being evaluated. By comparing the proposed PIFN performance against other optimization algorithms using f-rank, it is easy to determine whether it performs significantly better or not. For each test functions, the f-rank of every algorithm is displayed in the fourth row of the Table  8. The PIFN is ranked top among all other algorithms for the numerical (classical) test problems and is ultimately determined to be a statistically significant optimization algorithm

**Table 8:** Simulation outcomes of the FGIN algorithm in comparison to other MH algorithms for classical benchmark functions.

Function		MHA^53^	SaDE^100^	CMA-ES^66^	OEWOA^101^	LSHADE-SPACMA^65^	FA-FPO^67^	GWO-E^109^	SHADE^99^	SCCSA^111^	EO^70^	JADE^62^	PIFN
*F*1	mean	0.00E+00	4.50E−20	1.42E−18	7.75E−176	2.23E−01	1.51E−184	3.92E−67	1.42E−09	9.22E−69	3.32E−40	1.80E−60	0.00E+00
	std	0.00E+00	6.90E−20	3.13E−18	0.00E+00	1.48E−01	0.00E+00	1.11E−66	3.09E−09	3.81E−68	6.78E−40	8.40E−60	0.00E+00
	p-rank	−	−	−	−	−	−	−	−	−	−	−	
	f-rank	1	9	10	4	12	3	6	11	5	8	7	1
*F*2	mean	1.63E−180	1.90E−14	2.98E−07	1.86E−115	2.11E+01	5.04E−93	4.31E−36	8.70E−03	8.25E−41	7.12E−23	1.80E−25	6.21E−188
	std	0.00E+00	1.05E−14	1.78E+00	1.32E−114	9.57E+00	3.47E−93	6.57E−36	2.13E−02	4.19E−40	6.36E−23	8.8E−25	0.00E+00
	p-rank	−	−	−	−	−	−	−	−	−	−	−	
	f-rank	2	9	10	3	12	4	6	11	5	8	7	1
*F*3	mean	1.16E−318	9.00E−37	1.59E−05	2.87E+04	8.87E+01	1.23E−183	3.75E−37	1.54E+01	4.31E−13	8.06E−09	5.70E−61	0.00E+00
	std	0.00E+00	5.43E−36	2.21E−05	1.02E+04	4.72E+01	0.00E+00	1.36E−36	9.94E+00	2.83E−30	1.60E−08	2.70E−60	0.00E+00
	p-rank	−	−	−	−	−	−	−	−	−	−	−	
	f-rank	2	6	9	12	11	3	5	10	7	8	4	1
*F*4	mean	8.20E−24	7.40E−11	2.39E−25	1.06E+01	2.15E−17	9.97E−93	2.01E−06	9.79E−01	2.11E+00	5.39E−10	**1.00E−185**	1.32E−185
	std	0.00E+00	1.82E−10	1.25E−06	2.22E+01	4.92E−01	7.31E−93	6.80E−25	7.99E−01	1.06E−16	1.38E−09	4.00E−23	0.00E+00
	p-rank	+	−	−	−	−	−	−	−	−	−	−	
	f-rank	1	7	9	12	11	3	4	10	6	8	5	2
*F*5	mean	2.84E+01	2.10E+01	3.67E+01	2.85E+01	2.88E+01	2.89E+01	2.65E+01	2.44E+01	5.90E+00	2.53E+01	8.00E−02	2.86E+01
	std	3.23E−01	7.80E+00	3.34E+01	2.22E−01	8.24E−01	1.72E−02	5.19E−01	1.12E+01	9.13E−01	1.69E−01	5.60E−01	5.42E−02
	p-rank	−	−	−	−	−	−	−	−	+	−	+	
	f-rank	5	10	12	7	8	9	6	11	2	4	1	3
*F*6	mean	3.86E−01	9.30E+02	6.83E−19	1.62E+00	2.48E−01	5.88E+00	2.65E+01	5.31E−10	4.14E−08	8.29E−06	2.90E+00	4.28E−01
	std	1.28E−01	1.80E+02	6.71E−19	6.93E−01	1.13E−01	5.86E−01	5.19E−01	6.35E−10	5.22E−08	5.02E−06	1.20E+00	1.52E−01
	p-rank	+	−	+	−	+	−	−	+	+	+	−	
	f-rank	6	12	1	8	5	9	11	2	3	4	10	7
*F*7	mean	9.65E−05	4.80E−03	2.75E−02	1.37E−03	4.70E−03	1.13E−04	9.90E−04	2.35E−02	1.33E−03	1.17E−03	6.40E−04	7.59E−05
	std	1.19E−04	1.20E−03	7.90E−03	2.85E−03	1.90E−03	8.94E−04	8.37E−04	8.80E−03	1.72E−03	6.54E−04	2.50E−04	7.59E−05
	p-rank	−	−	−	−	−	−	−	−	−	−	−	
	f-rank	2	10	12	8	9	3	5	11	7	6	4	1
*F*8	mean	0.00E+00	1.20E−03	2.53E+01	0.00E+00	6.75E+01	0.00E+00	0.00E+00	8.53E+00	5.46E+00	0.00E+00	1.00E−04	0.00E+00
	std	0.00E+00	6.50E−04	8.55E+00	0.00E+00	1.00E+01	0.00E+00	0.00E+00	2.19E+00	5.62E+00	0.00E+00	6.00E−05	0.00E+00
	p-rank	$=$	−	−	$=$	−	$=$	$=$	−	−	$=$	−	
	f-rank	1	8	11	1	12	1	1	10	9	1	7	1
*F*9	mean	8.88E−16	2.70E−03	1.55E+01	3.02E−15	3.93E−02	8.88E−16	5.58E−15	3.95E−01	8.88E−16	8.34E−14	8.20E−10	4.44E−16
	std	2.53E−14	5.10E−04	7.92E+00	2.27E−15	1.51E−02	0.00E+00	1.67E−15	5.86E−01	9.36E−32	6.90E−10	0.00E+00	0.00E+00
	p-rank	−	−	−	−	−	−	−	−	−	−	−	
	f-rank	2	9	12	5	10	2	6	11	4	7	8	1
*F*10	mean	0.00E+00	7.80E−04	5.76E−15	1.42E−02	8.94E−01	0.00E+00	0.00E+00	4.80E−03	3.33E−02	0.00E+00	9.90E−08	0.00E+00
	std	0.00E+00	1.20E−03	6.18E−15	1.00E−01	1.07E−01	0.00E+00	0.00E+00	7.70E−03	4.56E−02	0.00E+00	6.00E−07	0.00E+00
	p-rank	$=$	−	−	−	−	$=$	$=$	−	−	$=$	−	
	f-rank	1	8	6	10	12	1	1	9	11	1	7	1
*F*11	mean	1.65E−02	1.90E−05	2.87E−16	1.06E−01	8.18E−04	8.32E−01	1.98E−02	3.46E−02	1.34E−02	7.97E−07	4.60E−17	1.71E−02
	std	8.20E−03	9.20E−06	5.64E−16	4.97E−02	1.00E−03	1.78E−01	1.01E−02	8.75E−02	1.60E−02	7.69E−07	1.90E−16	1.03E−02
	p-rank	+	+	+	−	+	−	−	−	−	+	+	
	f-rank	6	4	2	11	5	12	9	10	8	3	1	7
*F*12	mean	2.69E−01	6.10E−05	3.66E−04	1.03E+00	1.02E−02	2.94E+00	2.50E−01	7.32E−04	2.01E−02	2.92E−02	2.00E−16	2.53E−01
	std	8.12E−02	2.00E−05	2.00E−03	3.61E−01	1.03E−02	1.59E−01	1.63E−01	2.80E−03	7.23E−02	3.52E−02	6.50E−16	1.19E−01
	p-rank	−	+	+	−	+	−	−	+	+	+	+	
	f-rank	9	2	3	11	5	12	10	4	6	7	1	8
*F*13	mean	3.00E+00	3.00E+00	8.40E+00	3.00E+00	3.00E+00	3.00E+00	3.00E+00	3.00E+00	3.00E+00	3.00E+00	3.00E+00	3.00E+00
	std	5.13E−06	3.00E−15	2.05E+01	4.96E−04	1.25E−15	3.13E−09	1.25E−05	1.87E−15	8.93E−05	1.56E−15	1.10E−15	2.51E−10
	p-rank	−	+	−	−	+	−	−	+	−	+	+	
	f-rank	8	5	12	11	2	7	9	4	10	3	1	6
*F*14	mean	-3.86E+00	-3.86E+00	-3.86E+00	-3.86E+00	-3.86E+00	-3.01E−01	-3.86E+00	-3.86E+00	-3.86E+00	-3.86E+00	-3.86E+00	-3.86E+00
	std	4.32E−04	3.10E−15	2.70E−15	2.92E−04	2.70E−15	2.25E−16	4.16E−06	2.69E−15	9.29E−06	2.59E−15	0.00E+00	1.49E−05
	p-rank	−	+	+	−	+	+	+	+	−	+	+	
	f-rank	12	7	5	11	5	2	8	4	10	3	1	9
*F*15	mean	-3.21E+00	-3.31E+00	-3.29E+00	-3.24E+00	-3.28E+00	-3.29E+00	-3.26E+00	-3.27E+00	-3.26E+00	-3.26E+00	-3.31E+00	-3.23E+00
	std	4.13E−02	2.80E−02	5.35E−02	8.18E−02	5.70E−02	1.97E−02	7.50E−02	5.99E−02	6.00E−02	5.70E−02	3.60E−02	7.33E−02
	p-rank	+	+	+	−	+	+	−	+	+	+	+	
	f-rank	4	3	5	11	6	1	12	8	9	6	2	10
w/l/t		4/9/2	5/10/0	5/10/0	0/14/1	6/9/0	2/11/2	1/12/2	5/10/0	4/11/0	6/7/2	6/9/0	NA
Overall f-rank value		62	109	119	125	125	72	99	126	102	77	66	59
Overall f-rank		2	8	9	10	11	4	6	12	7	5	3	1

### Statistical outcomes for CEC 2017 benchmark numerical test problems

In this subsection, the reliability of the PIFN algorithm is further investigated utilizing the CEC 2017 test suite and compared with various competitive MH optimization techniques, including SHADE^102^, SaDE^103^, JADE^103^, MVMO^103^, CV1.0^104^, $CV_{new}$^105^, and CS algorithms are utilized. The benchmark comprises a collection of 29 mathematical test problems that exhibit varied levels of complexity, to evaluate the performance of optimization techniques. The problems can be classified into four distinct groups: unimodal problems (Fe1 and Fe3), multimodal problems ($Fe_4$-Fe10), composition problems (Fe11-F20), as well as hybrid problems (Fe21-Fe30). The complete descriptions of the problems IEEE CEC 2017 have been obtained from references^58^^60,114^. The experiments conducted are based on a dimension size of 50 with 51 run performed for the proposed PIFN algorithm. Table 9 presents the outcomes of the fitness value with regard to the error mean and std value. When the error value is less than E−08, it is considered to be zero.

- In the case of unimodal problems $Fe_1$, and $Fe_3$, the algorithms SaDE, JADE, SHADE, PIFN, and MVMO shows outstanding competitiveness and provide positive outcomes. Conversely, the algorithms CV1.0, CVnew, and CS demonstrate a lack of convergence. The PIFN algorithm has been found to be the most effective for problems $Fe_1$, and $Fe_3$ .
- Among all of the multimodal problems, it has been seen that SaDE, JADE, MVMO, PFIN and SHADE demonstrated superior performance for problems $Fe_4$ to $Fe_{10}$. Notably, PIFN shows the most effective algorithm among other MH algorithms.
- In the analysis of the third set of functions, specifically hybrid problems $Fe_{11}$ to $Fe_{20}$, it has been determined that PFIN, MVO, and $CV_new$ algorithms presented good performance while comparing with the other competitive algorithms.
- In the analysis of the final set of composite problems, denoted as $Fe_{21}$ to $Fe_{30}$, it has been seen that the PIFN algorithm consistently out performed all other MH algorithms.

The computational effectiveness of the PIFN algorithm is tested utilizing Wilcoxon’s (p-rank) as well as Friedman’s (f-rank) test. After the allocation of f-ranks, the average as well as overall f-rank are calculated in the last two rows of Table 9. There will be three scenarios that will occur while comparing PIFN namely those categorised as loss (l), win (w), or tie (t). The effectiveness of the proposed PFIN algorithm over previous MH algorithms in the most of challenges can be seen from the third last (w /l / t) row in table 9. Furthermore, the effectiveness of the PIFN algorithm algorithm is assessed by utilizing the f-rank test to see whether it exhibits a statistically significant improvement over other algorithms. In this test, each algorithm is assigned a rating depending on its performance. The last two rows of Table 9 presents the f-rank value as well as overall rank. The subsequent two rows provide evidence of the notable superiority of the proposed PIFN algorithm, which has attained the $I^{st}$ in the CEC 2017 benchmark problems. In short, it can be concluded that among all of the MH algorithms under comparison, the proposed PIFN algorithm, demonstrates outstanding competitiveness. Additionally, it is a potential candidate for becoming a state of the art algorithm.

**Table 9:** Comparison of statistical results of the PFIN algorithm with other MH algorithms for CEC-2017.

		SaDE^103^	JADE^103^	$CV_{new}$^105^	CS^114^	SHADE^102^	MVMO^103^	CV1.0^104^	PIFN
$Fe_1$	mean	1.21E+03	5.23E−14	1.00E+10	1.00E+10	0.00E+00	1.33E−05	1.00E+10	0.00E+00
	std	1.97E+03	2.51E−14	0.0000E+00	0.00E+00	0.00E+00	5.60E−06	0.0000E+00	0.00E+00
	rank	-	-	-	-	=	-	-	
	f-rank	5	3	6	6	1	4	6	1
$Fe_3$	mean	2.71E+02	1.77E+04	8.71E+03	2.52E+05	0.00E+00	5.30E−07	1.95E+04	0.00E+00
	std	8.28E+02	3.70E+04	4.08E+03	3.02E+04	0.00E+00	1.09E−07	6.27E+03	8.03E−15
	rank	-	-	-	-	=	-	-	
	f-rank	4	6	5	8	1	3	7	1
$Fe_4$	mean	8.92E+01	4.96E+01	2.67E+01	1.28E+02	5.68E+01	3.58E+01	1.16E+02	4.69E−01
	std	4.21E+01	4.71E+01	5.92E+00	2.44E+01	8.80E+00	3.66E+01	6.27E+03	1.29E+00
	rank	-	-	-	-	-	-	-	
	f-rank	6	4	2	8	5	3	7	1
$Fe_5$	mean	9.23E+01	5.42E+01	2.39E+02	4.86E+02	3.28E+01	8.07E+01	3.41E+02	2.03E+02
	std	1.86E+01	8.80E+00	3.80E+01	4.66E+01	5.03E+00	1.64E+01	8.02E+01	4.74E+01
	rank	+	+	-	-	+	+	-	
	f-rank	4	2	6	8	1	3	7	5
$Fe_6$	mean	7.43E−03	1.44E−13	4.07E+01	4.13E+01	8.38E−04	5.43E−03	4.85E+01	2.26E+01
	std	2.35E−02	9.11E−14	8.14E+00	6.32E+00	1.01E−03	3.30E−03	4.85E+01	4.42E+00
	rank	+	+	-	-	+	+	-	
	f-rank	4	1	6	7	2	3	8	5
$Fe_7$	mean	1.80E+02	1.01E+02	2.22E+02	5.51E+02	8.09E+01	1.23E+02	2.74E+02	5.11E+01
	std	1.97E+01	6.48E+00	3.49E+01	4.08E+01	3.78E+00	1.27E+01	7.29E+01	5.47E+01
	rank	-	-	-	-	+	-	-	
	f-rank	5	3	6	8	1	4	7	2
$Fe_8$	mean	9.42E+01	5.52E+01	2.59E+02	4.82E+02	3.23E+01	7.59E+01	3.29E+02	3.63E+01
	std	1.77E+01	7.76E+00	4.51E+01	4.67E+01	3.82E+00	1.61E+01	7.29E+01	6.77E+01
	rank	+	+	-	-	+	+	-	
	f-rank	5	3	6	8	1	4	7	2
$Fe_9$	mean	4.83E+01	1.17E+00	1.06E+04	3.53E+04	**1.11E+00**	7.38E+00	1.00E+04	1.61E+04
	std	6.29E+01	1.31E+00	3.10E+03	4.82E+03	9.37E−01	5.77E+00	2.90E+03	2.54E+03
	rank	+	+	-	-	+	+	-	
	f-rank	4	2	6	8	1	3	7	5
$Fe_{10}$	mean	6.60E+03	3.75E+03	6.09E+03	7.39E+03	3.34E+03	3.49E+03	7.10E+03	4.53E+03
	std	1.63E+03	2.54E+02	3.55E+02	3.26E+02	2.94E+02	4.31E+02	5.34E+02	4.02E+02
	rank	-	+	-	-	+	-	-	
	f-rank	6	2	5	8	1	4	7	3
$Fe_{11}$	mean	1.09E+02	1.36E+02	1.66E+02	1.18E+02	3.45E+02	1.20E+02	6.74E+01	1.23E+02
	std	3.54E+01	3.39E+01	1.91E+01	4.16E+01	2.93E+01	8.72E+00	3.38E+01	2.71E+01
	rank	+	-	-	-	+	+	-	
	f-rank	2	5	6	8	3	1	7	4
$Fe_{12}$	mean	1.11E+05	5.14E+03	1.00E+10	1.00E+10	5.13E+03	1.29E+03	1.00E+10	1.34E+05
	std	6.20E+04	3.32E+03	0.00E+00	0.00E+00	2.87E+03	2.79E+02	0.00E+00	1.74E+05
	rank	+	+	-	-	+	+	-	
	f-rank	4	3	6	6	2	1	6	5
$Fe_{13}$	mean	1.21E+03	3.03E+02	9.80E+09	1.00E+10	2.65E+02	4.37E+01	1.00E+10	1.24E+03
	std	1.45E+03	2.69E+02	1.40E+09	0.00E+00	1.49E+02	1.76E+01	0.00E+00	5.66E+02
	rank	-	+	-	-	+	+	-	
	f-rank	5	3	6	8	2	1	7	4
$Fe_{14}$	mean	2.88E+03	1.05E+04	3.98E+01	3.26E+05	2.15E+02	4.85E+01	2.05E+02	8.57E+02
	std	2.20E+03	3.11E+04	1.62E+01	1.60E+05	7.29E+01	1.21E+01	2.13E+01	1.57E+03
	rank	-	-	+	-	+	+	+	
	f-rank	6	7	1	8	4	2	3	5
$Fe_{15}$	mean	3.35E+03	3.49E+02	2.85E+02	7.85E+09	3.22E+02	4.46E+01	1.37E+09	3.26E+02
	std	2.79E+03	4.42E+02	3.54E+02	4.12E+09	1.42E+02	1.12E+01	3.47E+09	1.86E+02
	rank	-	-	-	-	-	+	-	
	f-rank	6	3	5	8	4	1	7	2
$Fe_{16}$	mean	8.17E+02	8.56E+02	1.44E+03	1.76E+03	7.33E+02	8.40E+02	1.53E+03	1.03E+03
	std	2.34E+02	1.75E+02	2.10E+02	2.37E+02	1.88E+02	1.93E+02	2.74E+02	1.81E+02
	rank	+	+	+	+	-	-	-	
	f-rank	3	4	6	8	2	1	7	5
$Fe_{17}$	mean	5.48E+02	6.00E+02	1.13E+02	1.18E+03	5.16E+02	5.19E+02	1.25E+03	5.12E+02
	std	1.53E+02	1.21E+02	1.92E+02	1.78E+02	1.11E+02	1.33E+02	1.85E+02	1.37E+02
	rank	+	+	+	-	+	+	-	
	f-rank	5	6	1	8	4	3	7	2
$Fe_{18}$	mean	3.24E+04	1.89E+02	1.51E+02	1.43E+06	1.89E+02	4.17E+01	5.21E+02	1.15E+02
	std	1.68E+04	1.25E+02	4.43E+01	5.89E+05	1.03E+02	1.94E+01	1.19E+02	4.28E+01
	rank	-	-	-	-	-	-	-	
	f-rank	7	6	2	8	5	4	3	1
$Fe_{19}$	mean	1.13E+04	3.24E+02	5.57E+01	1.99E+08	1.59E+02	1.73E+01	1.73E+02	3.11E+02
	std	1.68E+04	1.25E+03	1.10E+01	1.39E+09	568E+01	5.13E+00	4.17E+02	1.88E+02
	rank	-	-	+	-	-	+	-	
	f-rank	7	6	2	8	3	1	4	5
$Fe_{20}$	mean	3.52E+02	4.38E+02	2.81E+02	1.04E+03	3.33E+02	3.49E+02	1.05E+03	7.60E+02
	std	1.50E+02	1.33E+02	1.65E+02	1.67E+02	1.20E+02	1.47E+02	2.14E+02	1.45E+02
	rank	+	+	+	-	+	-	-	
	f-rank	3	4	1	8	2	6	7	5
$Fe_{21}$	mean	2.87E+02	2.59E+02	**1.18E+02**	6.55E+02	2.33E+02	2.77E+02	5.41E+02	2.23E+02
	std	1.36E+01	9.63E+00	8.77E+01	7.93E+01	5.11E+00	1.60E+01	6.27E+01	1.61E+01
	rank	-	-	+	-	-	-	-	
	f-rank	3	6	1	8	4	5	7	2
$Fe_{22}$	mean	2.92E+03	3.33E+03	5.77E+03	8.19E+03	3.17E+03	3.26E+03	7.33E+03	3.11E+03
	std	3.24E+03	1.80E+03	3.64E+02	4.08E+02	1.55E+03	1.71E+03	1.99E+03	1.01E+02
	rank	+	-	-	-	-	-	-	
	f-rank	1	3	7	8	4	5	6	2
$Fe_{23}$	mean	5.22E+02	4.79E+02	1.87E+02	9.14E+02	4.59E+02	5.04E+02	7.74E+02	7.97E+02
	std	2.05E+01	1.17E+01	5.11E+01	4.59E+01	8.75E+00	1.71E+03	8.06E+01	5.53E+01
	rank	+	+	+	-	+	+	-	
	f-rank	5	4	1	8	3	2	7	6
$Fe_{24}$	mean	5.89E+02	5.31E+02	3.25E+02	1.01E+03	5.31E+02	5.83E+02	8.32E+02	5.16E+02
	std	1.86E+01	7.62E+00	8.95E+01	6.38E+01	7.45E+00	1.69E+01	1.21E+01	2.88E+01
	rank	-	-	+	-	-	-	-	
	f-rank	6	4	1	8	3	5	7	2
$Fe_{25}$	mean	5.71E+02	5.19E+02	4.70E+02	5.33E+02	5.06E+02	5.09E+02	5.43E+02	4.31E+02
	std	3.05E+01	3.48E+01	2.26E+01	1.66E+01	3.64E+01	3.12E+01	1.51E+01	6.66E−03
	rank	-	-	-	-	-	-	-	
	f-rank	3	5	2	8	6	4	7	1
$Fe_{26}$	mean	2.52E+03	1.61E+03	1.16E+03	4.57E+03	1.41E+03	1.93E+03	2.48E+03	2.98E+02
	std	3.37E+02	1.21E+02	1.88E+03	1.56E+03	9.78E+01	2.86E+02	1.82E+03	1.40E+01
	rank	-	-	-	-	-	-	-	
	f-rank	7	4	2	8	5	6	3	1
$Fe_{27}$	mean	7.10E+02	5.50E+02	4.53E+02	8.17E+02	5.49E+02	5.43E+02	7.38E+02	5.00E+02
	std	6.65E+01	2.34E+01	7.17E+01	5.68E+01	2.78E+01	1.75E+01	8.21E+01	2.51E−04
	rank	-	-	-	-	-	-	-	
	f-rank	3	5	2	8	6	7	4	1
$Fe_{28}$	mean	4.99E+02	4.91E+02	4.58E+02	5.12E+02	4.79E+02	4.64E+02	4.94E+02	4.75E+02
	std	1.53E+01	2.08E+01	2.33E−01	1.88E+01	2.41E+01	1.50E+01	1.93E+01	2.49E+01
	rank	-	-	-	-	-	-	-	
	f-rank	5	2	6	8	3	4	7	1
$Fe_{29}$	mean	5.11E+02	4.77E+02	1.45E+03	1.57E+03	4.87E+02	4.89E+02	1.69E+03	8.15E+02
	std	1.37E+02	8.06E+01	1.68E+02	1.79E+02	1.05E+02	1.40E+01	2.29E+02	1.40E+02
	rank	+	+	-	-	+	+	-	
	f-rank	3	1	6	8	2	4	7	5
$Fe_{30}$	mean	8.07E+05	6.68E+05	6.02E+05	2.95E+09	6.82E+05	5.81E+05	4.64E+06	9.38E+02
	std	8.33E+04	9.25E+04	2.99E+04	4.59E+09	8.51E+04	1.02E+04	8.59E+06	8.40E+02
	rank	-	-	-	-	-	-	-	
	f-rank	7	4	3	8	5	6	2	1
w/l/t		12/17/0	12/17/1	7/22/0	0/29/0	14/14/1	14/15/0	1/28/0	
Average f-rank		4.62	4.34	4.03	7.82	2.97	3.44	6.24	2.93
Overall f-rank		6	5	4	8	2	3	7	1

Significant values are in bold.

### Statistical outcomes for CEC 2019 benchmark numerical test problems

In this subsection, the ten most challenging CEC 2019^98^ numerical problems ($np_{1}$ to $np_{10}$), compared with various competitive optimization techniques, including jDE100^63^ (winner of CEC 2019), DE^106^, YDSE^64^, FPA^107^, QLJAYA^82^ and NMRA^52^. Here, each optimization method runs for 51 cycles with 500 iterations for a population size of 50. The PIFN outperforms other competing algorithms for the numerical problems np6, np7, np8, and np10 in terms of the mean tabulated in Table 10. The outcomes of YDSE for numerical problem np4 are superior to those of all other techniques under consideration. The standard FPA is recognized as the optimal algorithm for numerical problems np5 and np9, respectively. The PIFN is recognized as the optimal optimization technique for numerical problems np1, np2, and np3. Overall performance of the PIFN performs better than other optimization methods for seven out of ten numerical problems, whereas QLJAYA, YDSE, PSO, and NMRA perform better in few cases.

Wilcoxon’s (p-rank) as well as Friedman’s (f-rank) test are used to verify the operational effectiveness of the PIFN algorithm. Once f-ranks have been allocated, the average and overall f-rank are computed in the final two rows of Table 10. Three situations will arise when the proposed algorithm (PIFN) is in contrast to existing algorithms (loss(l)/win(w)/ tie(t)). First, the loss condition arises when the algorithm’s performance is the worst compared to the proposed algorithm and assigns a “-” sign. The win condition occurs when the algorithm’s performance is superior to the proposed algorithm and assigns a “+” sign. And the last case is the tie condition, which occurs when both the algorithm’s performance is significant to the proposed algorithm and assigns the “=” sign. So, l/w/ t values are presented in Table 10. For these numerical problems, the PIFN algorithm proved to be a far superior optimization algorithm and ranked first.

**Figure 3:**
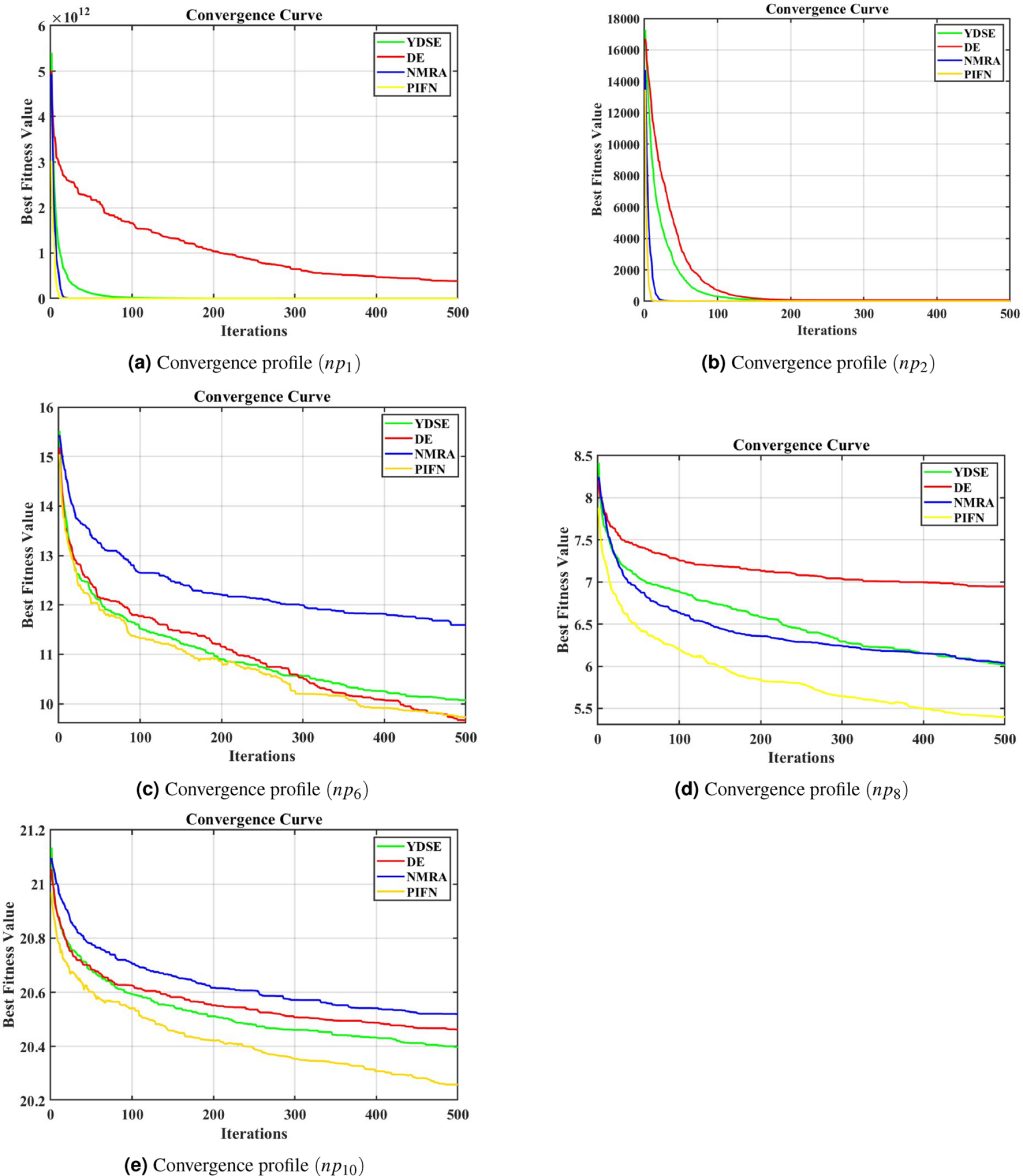
Convergence profiles of YDSE, DE, NMRA and PIFN for CEC 2019 numerical problems.

The convergence profiles of the DE, YDSE, NMRA, as well as PIFN algorithm are provided for five numerical problems in Fig. 3, together with statistical data. According to the Fig. 3, the PIFN algorithm converges faster than DE, YDSE, as well as NMRA for all CEC 2019 numerical problems ($np_1$, $np_2$, $np_6$, $np_8$ and $np_{10}$) considered. The PIFN algorithm has a consistently higher convergence rate compared to the YDSE, DE, as well as NMRA algorithms.

**Table 10:** Statistical outcomes for 100-digit challenge (CEC 2019) numerical problems.

Problem		jDE100^63^	DE^106^	FPA^107^	YDSE^64^	QLJAYA^82^	NMRA^52^	PIFN
$np_{1}$	Mean	1.590E+05	5.864E+11	5.41E+08	1.836E+06	5.239E+03	4.167E+05	9.2202E+04
	Std	1.597E+05	8.359E+11	2.939E+08	9.262E+05	1.655E+03	2.857E+05	2.2164E+04
	p-rank	−	−	−	−	+	−	
	f-rank	3	7	6	5	1	4	2
$np_{2}$	Mean	2.385E+06	6.844E+01	2.163E+01	1.735E+01	4.203E+03	1.891E+01	1.749E+01
	Std	2.719E+04	1.418E+02	2.6141E+00	4.300E−03	1.202E+03	5.381E−01	1.436E−01
	p-rank	−	−	−	+	−	−	
	f-rank	7	5	4	1	6	3	2
$np_{3}$	Mean	1.310E+06	1.270E+01	1.270E+01	1.270E+01	1.270E+01	1.270E+01	1.270E+01
	Std	8.519E+05	1.900E−03	9.0311E−08	3.494E−05	7.5409E−04	4.552E−04	1.393E−05
	p-rank	−	−	−	−	−	−	
	f-rank	7	6	1	4	3	5	2
$np_{4}$	Mean	3.475E+05	1.539E+03	1.343E+02	5.389E+01	5.140E+03	5.525E+02	2.792E+03
	Std	1.149E+05	2.433E+03	2.805E+01	1.037E+01	2.13E+03	5.488E+02	2.320E+03
	p-rank	−	−	+	+	−	+	
	f-rank	7	5	2	1	6	3	4
$np_{5}$	Mean	1.673E+05	2.116E+00	1.6368E+00	2.170E+00	3.160E+00	2.257E+00	2.670E+00
	Std	8.426E+04	2.850E−01	8.526E−02	6.468E−01	2.897E−01	5.225E−01	6.433E−01
	p-rank	−	+	+	+	−	+	
	f-rank	7	2	1	3	6	4	5
$np_{6}$	Mean	3.841E+04	9.901E+00	1.049E+01	1.007E+01	1.322E+01	1.159E+01	9.722E+00
	Std	2.063E+03	1.474E+00	6.747E−01	6.818E−01	9.101E−01	7.990E−01	9.411E−01
	p-rank	−	−	−	−	−	−	
	f-rank	7	2	4	3	6	5	1
$np_{7}$	Mean	9.105E+06	1.282E+03	2.662E+02	2.658E+02	1.423E+03	1.984E+02	4.833E+01
	Std	4.38E+08	4.224E+02	1.025E+02	1.193E+02	2.429E+02	1.612E+02	1.166E+02
	p-rank	−	−	−	−	−	−	
	f-rank	7	5	4	3	6	2	1
$np_{8}$	Mean	1.219E+09	6.982E+00	5.836E+00	6.007E+00	7.261E+00	6.039E+00	5.3964E+00
	Std	4.388E+08	2.255E−01	2.7187E−01	3.456E−01	3.869E−01	4.872E−01	4.309E−01
	p-rank	−	−	−	−	−	−	
	f-rank	7	5	2	3	6	4	1
$np_{9}$	Mean	9.207E+08	2.788E+02	4.947E+00	6.172E+01	1.482E+03	8.657E+00	3.952E+02
	Std	1.131E+08	3.122E+02	4.190E−01	1.523E+02	5.191E+02	2.002E+01	3.039E+02
	p-rank	−	+	+	+	−	+	
	f-rank	7	4	1	3	6	2	5
$np_{10}$	Mean	1.541E+06	2.042E+01	2.039E+01	2.039E+01	2.078E+01	2.051E+01	2.0274E+01
	Std	7.460E+05	1.295E−01	7.951E−02	1.034E−01	1.718E−01	1.367E−01	6.940E−02
	p-rank	−	−	−	−	−	−	
	f-rank	7	4	2	3	6	5	1
w/l/t		0/10/0	2/8/0	3/7/0	4/6/0	1/9/0	3/7/0	NA
Average f-rank		6.60	4.50	2.70	2.90	5.20	3.70	2.40
Overall f-rank		7	5	2	6	3	4	1

## Parametric estimation of PV systems

### Problem formulation

A reliable mathematical model is necessary to develop an effective design for solar cells. In literature, there are two common models: one takes into account an SDM, while the other employs a DDM. This section comprehensively illustrates SDM and DDM.

#### Single diode model

**Figure 4:**
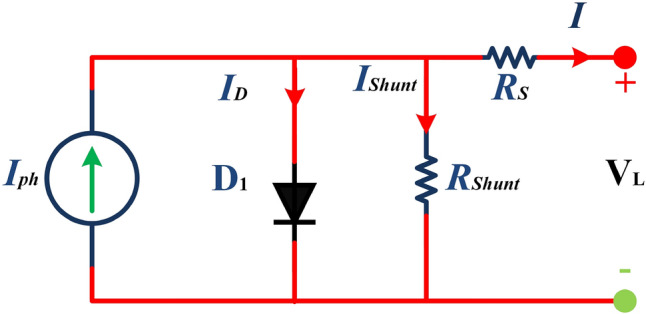
Equivalent model for PV cells’ SDM.

Figure 4 displays the equivalent model for PV cells’ SDM, which includes a current source $(I_{Ph})$ connected in parallel with a diode $(D_{1})$, a parallel resistor for leakage current $(I_{shunt})$, and a series resistor for losses resulting from load current (I)^81,115^. According to Kirchhoff’s Current Law (KCL), the output current of SDM is given in the Eq. (42).

42 $$\begin{aligned} I=I_{Ph}-I_{diode}-I_{shunt} \end{aligned}$$

Equations 43 and 44 provide the mathematical expressions for the $I_{diode}$ and $I_{shunt}$, respectively.

43 $$\begin{aligned} I_{diode}=I_{sc}\left[ exp\left( \frac{q(V_{L}+I*R_{s})}{nkT} \right) -1 \right] \end{aligned}$$

44 $$\begin{aligned} I_{shunt}=\frac{V_{L}+I*R_{s}}{R_{shunt}} \end{aligned}$$

The *I* is shown in the given Eq. (45).

45 $$\begin{aligned} I=I_{Ph}-I_{sc}\left[ exp\left( \frac{q(V_{L}+I*R_{shunt})}{nkT} \right) -1 \right] -\frac{V_{L}+I*R_{s}}{R_{shunt}} \end{aligned}$$

where reverse saturate current $(I_{sc})$, shunt resistance $(R_{shunt})$, series resistance $(R_{s})$, diode ideality factor (*n*), charge of the electron $(q=1.60217646 \times 10-19C)$, Boltzmann constant $(k=1.3806503 \times 10-23J/K)$, and Kelvin temperature of the solar cell (*T*). Since current hasn’t been explicitly represented as a function of voltage in 45, Newton-Raphson is used to solve the problem. A precise photovoltaic (PV) model can be developed by extracting the five unknown parameters ($I_{Ph}$, $I_{sc}$, $R_{shunt}$, $R_{s}$, and *n*).

#### Double diode model

**Figure 5:**
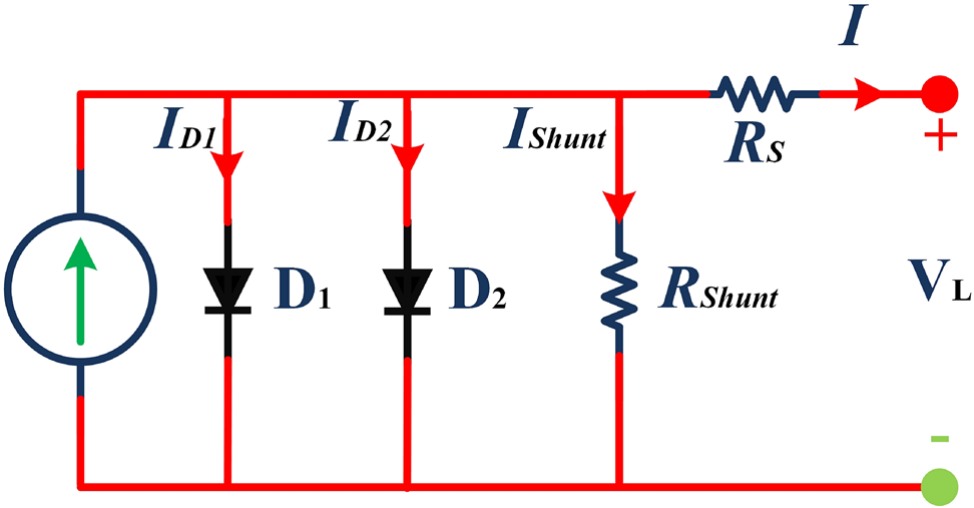
Equivalent model for PV cells’ DDM.

Figure 5 displays the equivalent model for PV cells’ DDM, which includes a current source $(I_{Ph})$ connected with two diodes in parallel $(D_{1}$, and $(D_{2})$, a parallel resistor for leakage current $(I_{shunt})$, and a series resistor for losses resulting from load current (I). According to Kirchhoff’s Current Law (KCL), the output current of DDM is given in the Eq. (46)^77,116^.

46 $$\begin{aligned} I=I_{Ph}-I_{diode1} - I_{diode2}-I_{shunt} \end{aligned}$$

Equations 47 and 48 provide the mathematical expressions for the $I_{diode1}$ and $I_{diode2}$, respectively.

47 $$\begin{aligned} I_{diode1}=I_{sc1}\left[ exp\left( \frac{q(V_{L}+I*R_{s})}{n_{1}kT} \right) -1 \right] \end{aligned}$$

48 $$\begin{aligned} I_{diode2}=I_{sc2}\left[ exp\left( \frac{q(V_{L}+I*R_{s})}{n_{2}kT} \right) -1 \right] \end{aligned}$$

The *I* is shown in the given Eq. (49).

49 $$\begin{aligned} I=I_{Ph}-I_{sc1}\left[ exp\left( \frac{q(V_{L}+I*R_{shunt})}{n_{1}kT} \right) -1 \right] -I_{sc2}\left[ exp\left( \frac{q(V_{L}+I*R_{shunt})}{n_{2}kT} \right) -1 \right] -\frac{V_{L}+I*R_{shunt}}{R_{shunt}} \end{aligned}$$

where, reverse saturate current $(I_{sc1})$ and and $I_{sc2}$, and diode ideality factor $(n_{1}))$ and and $(n_{2}))$. A precise photovoltaic (PV) model can be developed by extracting the seven unknown parameters($I_{Ph}$, $I_{sc1}$, $I_{sc2}$ $R_{shunt}$, $R_{s}$, $n_{1}$ and $n_{2}$).

### Objective function

The SDM and DDM parameter extraction is to identify a set of parameter values that will reduce the errors between estimated and measured current, which can be represented as an objective function. The primary objective of parameter extraction for SDM and DDM is to determine a set of parameter values that will minimize the errors between the estimated current as well as measured current, which an objective function can represent. As previously mentioned subsection, the SDM and DDM solar PV models comprise five ($I_{Ph}$, $I_{sc}$, $R_{shunt}$, $R_{s}$, and *N*) and seven ($I_{Ph}$, $I_{sc1}$, $I_{sc2}$ $R_{shunt}$, $R_{s}$, *N*2 and *N*2) parameters, respectively. The root means square error (RMSE) is employed as the objective function in this study to facilitate comparison with the reported outcomes of existing literature. The RMSE function is presented in the given Eq. (50)^83,117^.

50 $$\begin{aligned} F=\sqrt{\frac{1}{N}\sum _{i=1}^{N}\left[ I_{mess}-I_{cal} \right] ^{2}} \end{aligned}$$

The individual absolute error $IAE_{current}$ and $IAE_{Power}$ in the given Eqs. (51) and  (52).

51 $$\begin{aligned} IAE_{Current}=\sum _{i=1}^{N}\left| I_{mess}-I_{cal} \right| \end{aligned}$$

52 $$\begin{aligned} IAE_{Power}=\sum _{i=1}^{N}\left| P_{mess}-P_{cal} \right| \end{aligned}$$

Te relative error (RE), mean bias error (MBE), mean absolute percentage error (MAPE), and mean absolute error (MAE), is presented in the following Eqs.  (53),  (54),  (55) and  (56), respectively^91^.

53 $$\begin{aligned} RE = \frac{I_{mess}-I_{cal}}{I_{mess}} \end{aligned}$$

54 $$\begin{aligned} MBE=\frac{1}{N}\sum _{i=1}^{N} \left( I_{mess}-I_{cal} \right) \end{aligned}$$

55 $$\begin{aligned} MAPE=\frac{100 \%}{N}\sum _{i=1}^{N}\left| \frac{I_{mess}-I_{cal} }{I_{mess}} \right| \end{aligned}$$

56 $$\begin{aligned} MAE=\frac{1}{N}\left[ I_{mess}-I_{cal} \right] \end{aligned}$$

where, measured current $(I_{mess})$ , calculated current $(I_{cal})$, measured power $(P_{mess})$, and calculated power $(P_{cal})$, and number of samples (*N*).

### Results on solar PV parameter estimation challenges

This section evaluates the PIFN algorithm’s effectiveness in estimating parameters for various photovoltaic (PV) models (RTC France Solar Cell (SDM, and DDM), Photowatt-PWP201, STM6- 40/36, and STP6-120/36 module). The paper’s objective is to evaluate the SDM and DDM of a PV cell’s five unknown variables ($I_{Ph}$, $I_{sc}$, $R_{shunt}$, $R_{s}$, and *n*) as well as the seven unknown variables ($I_{Ph}$, $I_{sc1}$, $I_{sc2}$ $R_{shunt}$, $R_{s}$, $n_{1}$ and $n_{2}$). Table  11 shows , the lower limit (LL) and upper limit (UL) for the RTC France Solar Cell (SDM, and DDM)^73^, Photowatt-PWP201^73^, STM6- 40/36^118^, and STP6-120/36 module^71^.

**Table 11:** Parameters limits of PV cell/models.

Parameters	SDM/DDM^73^		STP6-120/36^71^		STM6-40/36^118^		Photowatt-PWP201^73^	
	LL	UL	LL	UL	LL	UL	LL	UL
$I_{Ph}\; \; (A)$	0	1	0	8	0	2	0	2
$I_{sc1},\; I_{sc2} \;or\; I_{o1},I_{o2} (\mu A)$	0,0	1,1	0	50	0	50	0	50
$Rs\; (\Omega )$	0	0.5	0	0.36	0	0.36	0	2
$Rshunt\; or\; Rsh \; (\Omega )$	0	100	0	1500	0	1000	0	2000
$n_{1},n_{2}$	1,1	2,2	1	50	1	60	1	50

#### Comparative analysis for the R.T.C.France

**Figure 6:**
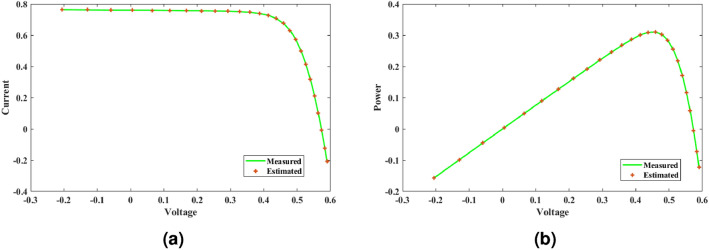
Comparisons of the (**a**) I-V (**b**) P-V characteristics for the SDM between actual and simulated data obtained by PIFN.

**Figure 7:**
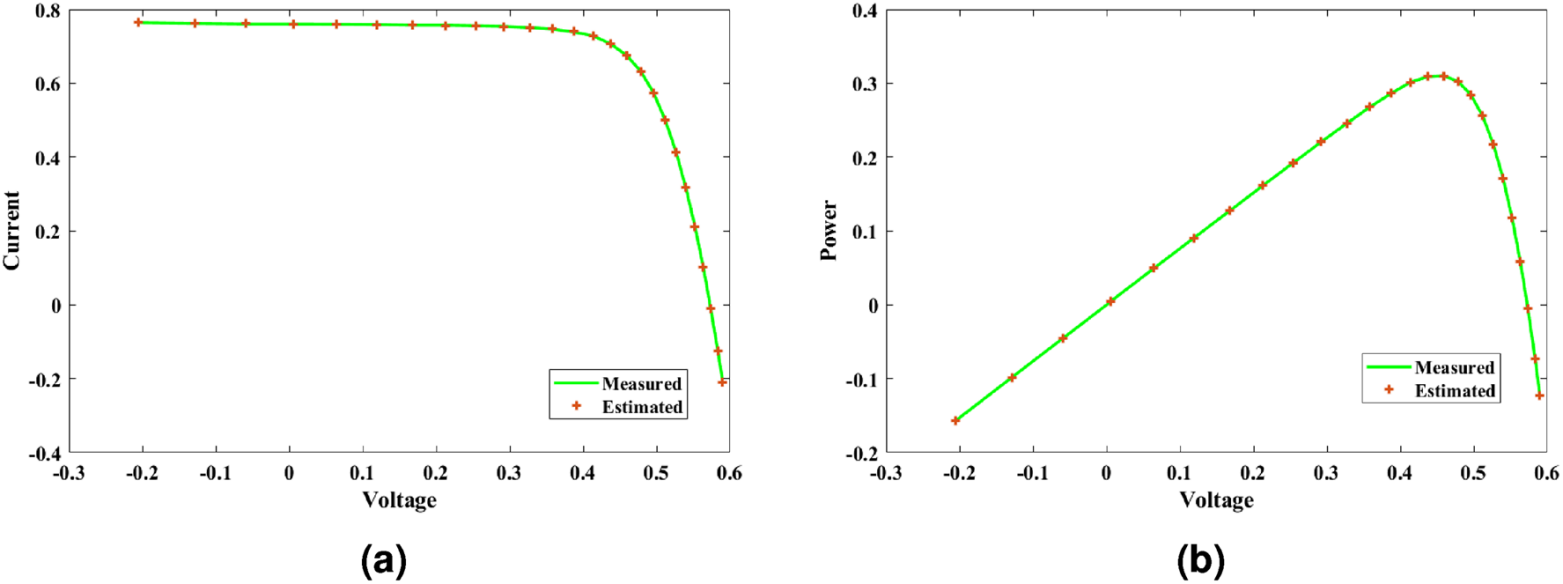
Comparisons of the (**a**) I-V (**b**) P-V characteristics for the DDM between actual and simulated data obtained by PIFN.

Five and seven parameters of a 57 mm diameter commercial (R.T.C.France) silicon solar cell are extracted for SDM and DDM using PIFN optimization techniques when operating at 33 $^{\circ}$C and 1000 w/m^2^ irradiance^71,80^. The measured as well as computed values (Im, Ie, V, Pm, Pe, IAE (Current), IAE (Power), MAE, RE, MBE, as well as MAPE) for SDM and DDM of RTC France are presented in Tables  12, 13 and  14, the best IAE values of the SDM for current and power are 1.76E−02, and 6.84E−03, respectively. In addition to this, the best IAE values of the DDM for current and power are 1.75E−02 and 6.6236E−03, respectively. Tables  13 and  15 compares the minimum (Min), Mean, and standard deviation (Std) values (the best outcomes are highlighted in bold) of the proposed algorithm with the other MH optimization techniques. The best RMSE values for SDM, and DDM are 7.72E−04, and 7.59E−04, respectively. All of the IAE values are less than 2.508E−03, demonstrating the high accuracy of the PIFN-estimated parameters. The I-V as well as P-V curves for SDM have been replicated employing the most optimal estimated PIFN parameters in order to provide additional evidence about the precision of the outcomes, as seen in Fig. 6a,b. Also for the DDM, The I-V as well as P-V curves have been replicated employing the most optimal estimated PIFN parameters in order to provide additional evidence about the precision of the outcomes, as seen in Fig. 7a,b.

**Table 12:** The PIFN outcomes of Ie, IAE, MAE, MBE, MAPE and RE for the SDM.

S. no.	Ie	Im	IAE	V	Pe	Pm	IAE	RE
1	0.764147027	0.76400	0.000147	− 0.2057	− 0.15719	− 0.15715	3.02E−05	− 0.00019
2	0.762699679	0.76200	0.0007	− 0.1291	− 0.09846	− 0.09837	9.03E−05	− 0.00092
3	0.761371269	0.76050	0.000871	− 0.0588	− 0.04477	− 0.04472	5.12E−05	− 0.00115
4	0.760151973	0.7605	0.000348	0.0057	0.004333	0.004335	1.98E−06	0.000458
5	0.759036491	0.7600	0.000964	0.0646	0.049034	0.049096	6.22E−05	0.001268
6	0.758008162	0.7590	0.000992	0.1185	0.089824	0.089942	0.000118	0.001307
7	0.757043061	0.7570	4.31E−05	0.1678	0.127032	0.127025	7.23E−06	-5.7E−05
8	0.756082116	0.7570	0.000918	0.2132	0.161197	0.161392	0.000196	0.001213
9	0.755019496	0.75550	0.000481	0.2545	0.192152	0.192275	0.000122	0.000636
10	0.753594239	0.75400	0.000406	0.2924	0.220351	0.22047	0.000119	0.000538
11	0.751323692	0.750500	0.000824	0.32690	0.245608	0.245338	0.000269	− 0.0011
12	0.747301088	0.74650	0.000801	0.3585	0.267907	0.26762	0.000287	− 0.00107
13	0.740079504	0.73850	0.001580	0.38730	0.286633	0.286021	0.000612	− 0.00214
14	0.727420142	0.72800	0.00058	0.4137	0.300934	0.301174	0.00024	0.000797
15	0.70701934	0.70650	0.000519	0.43730	0.30918	0.308952	0.000227	− 0.00074
16	0.67539401	0.67550	0.000106	0.459	0.310006	0.310055	4.86E−05	0.000157
17	0.630993296	0.63200	0.001007	0.4784	0.301867	0.302349	0.000482	0.001593
18	0.572172348	0.57300	0.000828	0.496	0.283797	0.284208	0.000411	0.001444
19	0.499539644	0.49900	0.00054	0.5119	0.255714	0.255438	0.000276	− 0.00108
20	0.413488357	0.41300	0.000488	0.5265	0.217702	0.217445	0.000257	− 0.00118
21	0.317166854	0.316500	0.000667	0.5398	0.171207	0.170847	0.00036	− 0.00211
22	0.212022177	0.21200	2.22E−05	0.5521	0.117057	0.117045	1.22E−05	− 0.0001
23	0.102640638	0.10350	0.000859	0.56330	0.057817	0.058302	0.000484	0.008303
24	− 0.009298004	− 0.01	0.000702	0.57360	− 0.00533	− 0.00574	0.000403	0.0702
25	− 0.124366124	− 0.123	0.001366	0.5833	− 0.07254	− 0.07175	0.000797	− 0.01111
26	− 0.209111694	− 0.21	0.000888	0.59	− 0.12338	− 0.1239	0.000524	0.00423
IAE (Current)			1.76E−02	IAE (Power)			0.006487	
MAE		0.000678662	MBE		2.35E−06	MAPE		0.442624

**Table 13:** PIFN versus other algorithms on the SDM module under extracted parameter values.

S. no.	Optimization techniques	RMSE			IAE (Current)	Iph	Io	RSh	Rs	n	p-rank	f-rank
		Min	Mean	Std.								
1	PIFN	**7.72E−04**	**8.99E−04**	**1.42E−04**	**1.76E−02**	0.7608	0.548	63.987	0.0338	1.536		1
2	I-CPA^76^	9.9862E−04	4.44E−03	4.2832E−03	-	0.760577	0.32563	56.80359	0.036402	1.481923	−	16
3	TSA^77^	9.9339E−04	-	-	-	0.7606	0.3298	56.5694	0.0363	1.4832	−	15
4	IWOA^78^	9.86E−04	9.95E−04	1.12E−05	1.77E−02	0.760	0.323	53.7317	0.0364	1.481	−	2
5	DE^71^	9.86E−04	1.01E−03	6.72E−05	1.81E−02	0.7608	0.323	53.7185	0.0364	1.481	−	2
6	TLABC^71,79^	9.86E−04	9.98E−04	1.86E−05	2.03E−02	0.7608	0.323	53.716	0.036	1.4812	−	2
7	FIPSO-SQP^80^	9.86E−04	-	-	-	0.7608	0.3226	53.68	0.0363	1.4810	−	2
8	QLJAYA^82^	9.86E−04	9.86E−04	2.39E−17	-	0.7607	0.3230	53.7185	0.03637	1.48119	−	2
9	BES^84^	9:86E−04	9:86E−04	2.63E−13	2.39E−02	0.7607	0.323	53.718	0.0364	1.481	−	2
10	GSK^71^	9.86E−04	9.86E−04	2.18E−17	2.54E−02	0.7608	0.3231	53.722	0.0364	1.481	−	2
11	HBA^85^	9.86E−04	9.86E−04	7.81E−10	2.56E−02	0.7608	0.323	53.718	0.0364	1.481	−	2
12	HBA-OBL^85^	9.86E−04	9.86E−04	2.23E−10	2.65E−02	0.7608	0.323	53.718	0.0364	1.4812	−	2
13	MSA^86^	9.86E−04	-	-	-	0.7607755	0.3	53.7185260	0.0363771	1.4811836	−	2
14	MDMO^87^	9.8602E−04	-	-	-	0.76077	0.3230	53.719	0.03637	1.4811	−	12
15	GOTLBO^71,119^	9.87E−04	1.09E−03	1.36E−04	2.33E−02	0.7608	0.342	53.859	0.0362	1.487	−	13
16	HBO^90^	9.88E−04	1.50E−04	4.09E−04	2.19E−02	0.760	0.319	52.156	0.0364	1.480	−	14
17	WOA^78^	1.03E−03	3.08E−03	2.21E−03	1.84E−02	0.7606	0.388	60.562	0.0357	1.499	−	17

**Table 14:** The PIFN outcomes of Ie, IAE and RE for the DDM.

S. no.	Ie	Im	IAE	V	Pe	Pm	IAE	RE
1	0.763718	0.76400	0.000282	− 0.2057	− 0.1571	− 0.15715	5.8E−05	0.000369
2	0.762393	0.76200	0.000393	− 0.1291	− 0.09842	− 0.09837	5.08E−05	− 0.00052
3	0.761177	0.760500	0.000677	− 0.0588	− 0.04476	− 0.04472	3.98E−05	− 0.00089
4	0.760059	0.76050	0.000441	0.0057	0.004332	0.004335	2.51E−06	0.000579
5	0.759034	0.7600	0.000966	0.0646	0.049034	0.049096	6.24E−05	0.001271
6	0.758083	0.75900	0.000917	0.1185	0.089833	0.089942	0.000109	0.001208
7	0.757178	0.75700	0.000178	0.1678	0.127054	0.127025	2.99E−05	− 0.00024
8	0.756255	0.75700	0.000745	0.2132	0.161234	0.161392	0.000159	0.000984
9	0.755201	0.755500	0.000299	0.2545	0.192199	0.192275	7.62E−05	0.000396
10	0.753749	0.75400	0.000251	0.2924	0.220396	0.22047	7.34E−05	0.000333
11	0.751416	0.75050	0.000916	0.3269	0.245638	0.245338	0.000299	− 0.00122
12	0.747303	0.74650	0.000803	0.3585	0.267908	0.26762	0.000288	− 0.00108
13	0.739981	0.7385	0.001481	0.3873	0.286595	0.286021	0.000574	− 0.00201
14	0.727238	0.728	0.000762	0.4137	0.300858	0.301174	0.000315	0.001047
15	0.706798	0.7065	0.000298	0.4373	0.309083	0.308952	0.00013	− 0.00042
16	0.675189	0.6755	0.000311	0.459	0.309912	0.310055	0.000143	0.00046
17	0.630854	0.632	0.001146	0.4784	0.301801	0.302349	0.000548	0.001813
18	0.572121	0.573	0.000879	0.496	0.283772	0.284208	0.000436	0.001534
19	0.499569	0.499	0.000569	0.5119	0.255729	0.255438	0.000291	− 0.00114
20	0.41357	0.413	0.00057	0.5265	0.217745	0.217445	0.0003	− 0.00138
21	0.317267	0.3165	0.000767	0.5398	0.17126	0.170847	0.000414	− 0.00242
22	0.212109	0.212	0.000109	0.5521	0.117105	0.117045	6E−05	− 0.00051
23	0.102693	0.1035	0.000807	0.5633	0.057847	0.058302	0.000454	0.007793
24	− 0.00929	− 0.01	0.000711	0.5736	− 0.00533	− 0.00574	0.000408	0.071126
25	− 0.1244	− 0.123	0.001402	0.5833	− 0.07256	− 0.07175	0.000818	− 0.0114
26	− 0.20918	− 0.21	0.000822	0.59	− 0.12342	− 0.1239	0.000485	0.003912
IAE (Current)			0.0175	IAE (Power)			0.006624	
MAE		0.000673	MBE		3.52E−05	MAPE		0.446332

**Table 15:** PIFN versus other algorithms on the DDM module under extracted parameter values.

S. no.	Optimization techniques	RMSE			IAE (Current)	Iph	Io1	Io2	RSh	Rs	$n_{1}$	$n_{2}$	p-rank	f-rank
		Min	Mean	Std.										
1	PIFN	**7.59E−04**	**1.03E−03**	**2.14E−04**	**1.75E−02**	0.7604	0.2750	0.3745	63.7688	0.0352	1.942754	1.4989		1
2	I-CPA^76^	1.02E−03	4.35E−03	3.04E−03	-	0.760190	0.040418	0.28693	63.0435	0.036475	1.522657	1.477916	−	14
3	TSA^77^	9.88E−04	-	-	-	0.7609	0.21658	0.26741	52.5696	0.0367	1.4503	1.7552	−	13
4	ITLBO^120^	9.80E−04	9.80E−04	1.33E−05	1.91E−02	0.760	0.320	0.845	53.721	0.0361	1.474	1.789	−	2
5	TLABC^71,79^	9.80E−04	9.80E−04	2.06E−05	1.97E−02	0.760	0.423	0.240	54.66	0.0367	1.456	1.907	−	2
6	DE^71^	9.80E−04	9.90E−04	3.87E−05	2.28E−02	0.7608	0.3564	0.576	54.399	0.0366	1.457	1.997	−	2
7	RTLBO^121^	9.80E−04	9.90E−04	1.95E−05	2.50E−02	0.7608	0.229	0.85	49.085	0.0363	1.455	1.961	−	2
8	GOTLBO^119^	9.80E−04	1.00E−04	9.28E−05	2.87E−02	0.7608	0.341	0.263	54.414	0.034	1.463	1.991	−	2
9	GSK^71^	9.80E−04	9.80E−04	8.72E−07	2.97E−02	0.7608	0.2595	0.479	54.933	0.0366	1.462	1.998	−	2
10	IWOA^78^	9.83E−04	9.97E−04	1.92E−05	1.74E−02	0.7608	0.677	0.235	55.408	0.036	2	1.454	−	8
11	HBA^85^	9.83E−04	9.82E−04	1.50E−07	2.30E−02	0.760	0.231	0	55.264	0.0367	1.453	2	−	8
12	MSA^86^	9.827E−04	-	-	-	0.76078221	0.24301	0.604558	55.12	0.03665767	1.4571	1.9969	−	8
13	MDMO^87^	9.832E−04	-	-	-	0.76077	0.4278	0.2633	54.704	0.03658	1.9919	1.4638	−	8
14	HBA-OBL^85^	9.83E−04	9.83E−04	1.87E−07	2.54E−02	0.7608	0.671	0	55.3172	0.0367	2	1.454	−	8
15	HBO^90^	1.05E−03	1.56E−03	3.86E−04	2.33E−02	0.7607	0.67	0.197	51.676	0.0368	1.90873	1.4407	−	15
16	WOA^78^	1.13E−03	3.35E−03	1.66E−03	1.94E−02	0.761	0.365	0.127	55.564	0.0354	1.497	1.796	−	16

#### Comparative analysis for the STP6-120/36 module

**Figure 8:**
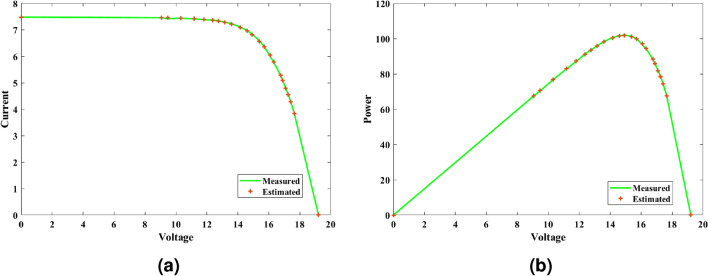
Comparisons of the (**a**) I-V (**b**) P-V characteristics for the STP6-120/36 module between actual and simulated data obtained by PIFN.

Five parameters of this module are extracted for SDM using PIFN optimization techniques when operating at 55 $^{\circ}$C and 1000 w/m^2^ irradiance^71,85^. Five unknown SDM parameters has been estimated for this module using PIFN utilizing experimental data. The measured, as well as computed values (Ie, Im, V, Pe, Pm, IAE (current), IAE (Power), RE, MAE, MBE, and MAPE) of STP6-120/36 module are presented in Table  16. The best IAE (current) and IAE (Power) values are 2.71E−01 and 3.87E+00. The table  17 presents the optimal model parameters, the RMSE, and the IAE obtained by the proposed PIFN and others algorithms reported in the literature for SDM. The best RMSE value is 1.44E−02. The I-V as well as P-V curves for SDM have been replicated employing the most optimal estimated PIFN parameters in order to provide additional evidence about the precision of the outcomes, as demonstrated in the Fig. 8a,b

**Table 16:** The PIFN outcomes of Ie, IAE and RE for the STP6-120/36 module.

S. no.	Ie	Im	IAE	V	Pe	Pm	IAE	RE
1	7.47356	7.48	0.00644	0	0	0	0	0.000861
2	7.450737	7.45	0.000737	9.06	67.50368	67.497	0.006677	-9.9E−05
3	7.447456	7.42	0.027456	9.47	70.52741	70.2674	0.260012	− 0.0037
4	7.437143	7.44	0.002857	10.32	76.75132	76.7808	0.029485	0.000384
5	7.418493	7.41	0.008493	11.17	82.86457	82.7697	0.094867	− 0.00115
6	7.394451	7.38	0.014451	11.81	87.32847	87.1578	0.170666	− 0.00196
7	7.362324	7.37	0.007676	12.36	90.99833	91.0932	0.094873	0.001041
8	7.330979	7.34	0.009021	12.74	93.39668	93.5116	0.114922	0.001229
9	7.284202	7.29	0.005798	13.16	95.8601	95.9364	0.076302	0.000795
10	7.2185	7.23	0.0115	13.59	98.09941	98.2557	0.156289	0.001591
11	7.089806	7.1	0.010194	14.17	100.4626	100.607	0.144445	0.001436
12	6.960689	6.97	0.009311	14.58	101.4868	101.6226	0.135756	0.001336
13	6.817439	6.83	0.012561	14.93	101.7844	101.9719	0.187538	0.001839
14	6.570606	6.58	0.009394	15.39	101.1216	101.2662	0.144566	0.001428
15	6.351162	6.36	0.008838	15.71	99.77676	99.9156	0.138838	0.00139
16	6.039295	6	0.039295	16.08	97.11186	96.48	0.631858	− 0.00655
17	5.777921	5.75	0.027921	16.34	94.41122	93.955	0.456221	− 0.00486
18	5.273427	5.27	0.003427	16.76	88.38264	88.3252	0.057439	− 0.00065
19	5.081065	5.07	0.011065	16.9	85.87	85.683	0.187003	− 0.00218
20	4.784195	4.79	0.005805	17.1	81.80973	81.909	0.099266	0.001212
21	4.544098	4.56	0.015902	17.25	78.38569	78.66	0.274314	0.003487
22	4.271188	4.29	0.018812	17.41	74.36138	74.6889	0.327515	0.004385
23	3.82885	3.83	0.00115	17.65	67.5792	67.5995	0.020299	0.0003
24	0.003503	0	0.003503	19.21	0.067287	0	0.067287	0
IAE (Current)			2.71E−01	IAE (Power)			3.87E+00	
MAE		0.011316924	MBE		-4.53786E−05	MAPE		0.182729659

**Table 17:** PIFN versus other algorithms on the STP6-120/36 module under extracted parameter values.

S. no.	Optimization techniques	RMSE			IAE (Current)	Iph	Io	RSh	Rs	n	p-rank	f-rank
		Min	Mean	Std.								
1	PIFN	1.44E−02	1.64E−02	2.26E−03	2.71E−01	7.47580	1.9675	561.4019	0.1685	45.4091		1
2	HBA-OBL^85^	1.60E−02	1.66E−02	1.31E−05	2.98E−01	7.4725	2.335	799.8886	0.1654	46.7896	−	2
3	HBA^85^	1.60E−02	1.66E−02	1.32E−05	3.62E−01	7.4725	2.335	799.929	0.1654	46.7896	−	2
4	RTLBO^121^	1.66E−02	2.08E−02	8.16E−04	2.87E−01	7.4728	2.3167	21.6438	0.0046	1.2594	−	4
5	DE^71^	1.66E−02	2.23E−02	5.13E−03	3.28E−01	7.4708	2.5614	28.8094	0.0046	1.2657	−	4
6	GSK^71^	1.66E−02	1.66E−02	1.44E−16	3.64E−01	7.4725	2.335	22.2199	0.0046	1.26	−	4
7	TLABC^79^	1.67E−02	1.73E−02	5.70E−04	2.88E−01	7.5611	3.4715	23.6694	0.0049	1.2698	−	7
8	GAMNU^92^	1.673E−02	-	-	2.92E−01	7.4690	2.7390	1468.618	0.16269	45.84837	−	7
9	GOTLBO^119^	1.67E−02	2.16E−02	3.45E−03	3.65E−01	7.4563	2.2559	22.4749	0.0045	1.2566	−	7
10	BES^84^	1.68E−02	1.69E−02	1.12E−04	3.30E−01	7.4716	2.3218	23.0265	0.0046	1.2596	−	10

#### Comparative analysis for the STM6-40/36 module

**Figure 9:**
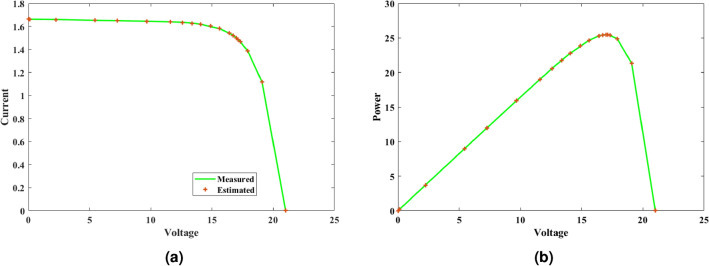
Comparisons of the (**a**) I-V (**b**) P-V characteristics for the STM6-40/36 module between actual and simulated data obtained by PIFN.

Five unknown SDM parameters has been estimated for the STM-40/36 module using PIFN utilizing experimental data^71,85^. The measured, as well as computed values (Ie, Im, V, Pe, Pm, IAE current, IAE power, RE, MAE, MBE, and MAPE) of STM-40/36 module, are presented in Table  18. The best IAE current and IAE Power values are 2.17E−02 and 2.83E−01. The Fig. 9a,b show the measured and calculated I-V and P-V curves based on the unknown parameters extracted for this module, demonstrating the consistency of PIFN. In addition, the Table  19 presents the optimal model parameters, the RMSE, and the IAE obtained by the proposed PIFN and others algorithms reported in the literature for SDM. The best RMSE value is 1.723E−03.

**Table 18:** The PIFN outcomes of Ie, IAE, MAE, MBE, MAPE and RE for the STM6-40/36 module.

S. no.	Ie	Im	IAE	V	Pe	Pm	IAE	RE
1	1.663382857	1.663	0.000383	0	0	0	0	− 0.00023
2	1.663179233	1.663	0.000179	0.118	0.196255	0.196234	2.11E−05	− 0.00011
3	1.659518847	1.661	0.001481	2.237	3.712344	3.715657	0.003313	0.000892
4	1.653942375	1.653	0.000942	5.434	8.987523	8.982402	0.005121	− 0.00057
5	1.650625998	1.65	0.000626	7.26	11.98354	11.979	0.004545	− 0.00038
6	1.645524962	1.645	0.000525	9.68	15.92868	15.9236	0.005082	− 0.00032
7	1.639336711	1.64	0.000663	11.59	18.99991	19.0076	0.007688	0.000404
8	1.633808017	1.636	0.002192	12.6	20.58598	20.6136	0.027619	0.00134
9	1.627364102	1.629	0.001636	13.37	21.75786	21.77973	0.021872	0.001004
10	1.618365325	1.619	0.000635	14.09	22.80277	22.81171	0.008943	0.000392
11	1.603079118	1.597	0.006079	14.88	23.85382	23.76336	0.090457	− 0.00381
12	1.581555684	1.581	0.000556	15.59	24.65645	24.64779	0.008663	− 0.00035
13	1.542254326	1.542	0.000254	16.4	25.29297	25.2888	0.004171	− 0.00016
14	1.521140471	1.524	0.00286	16.71	25.41826	25.46604	0.047783	0.001876
15	1.499116063	1.5	0.000884	16.98	25.45499	25.47	0.015009	0.000589
16	1.485181168	1.485	0.000181	17.13	25.44115	25.43805	0.003103	− 0.00012
17	1.465556035	1.465	0.000556	17.32	25.38343	25.3738	0.009631	− 0.00038
18	1.387550677	1.388	0.000449	17.91	24.85103	24.85908	0.008047	0.000324
19	1.11858328	1.118	0.000583	19.08	21.34257	21.33144	0.011129	− 0.00052
20	-5.2306E−05	0	5.23E−05	21.02	− 0.0011	0	0.001099	0
IAE (Current)			0.021717	IAE (Power)			0.283296	
MAE		0.001086	MBE		-6.5E−07	MAPE		0.068872382

**Table 19:** PIFN versus other algorithms on the STM6-40/36 module under extracted parameter values.

S. no.	Optimization techniques	RMSE			IAE (Current)	Iph	Io	RSh	Rs	n	p-rank	f-rank
		Min	Mean	Std.								
1	PIFN	1.723E−03	2.878E−03	1.379E−03	2.171E−02	1.6638	1.80195	579.8330	0.14964	54.8728		1
2	I-CPA^76^	2.1566E−03	1.5146E−02	1.0656E−04	-	1.661132	2.6044	20.60004	0.003057	1.565799	−	15
3	RTLBO^71,121^	1.729E−03	1.901E−03	2.29E−04	2.25E−02	1.663	1.702	15.828	0.0043	1.5180	−	2
4	BES^84^	1.729E−03	1.729E−03	5.6525E−18	2.486E−02	1.663	1.738	15.928	0.0043	1.5203	−	2
5	TLABC^71,79^	1.729E−03	2.002E−03	2.04E−04	2.49E−02	1.700	1.633	15.400	0.0050	1.5002	−	2
6	HBA-OBL^85^	1.729E−03	1.7298E−03	1.0079E−08	3.26E−02	1.663	1.7380	573.418	0.153	55.763	−	2
7	HBA^85^	1.729E−03	1.729E−03	4.671E−09	3.648E−02	1.663	1.738	573.418	0.153	55.763	−	2
8	GSK^71^	1.729E−03	1.729E−03	6.25E−18	3.949E−02	1.663	1.924	16.554	0.004	1.531	−	2
9	ESNSA^93^	1.72981E−03	1.72981E−03	1.401E−17	-	1.6639	1.73866	15.9282	0.004273	1.52030	−	2
10	ITLBO^71,120^	1.73E−03	1.74E−03	3.47E−05	2.23E−02	1.663	1.761	15.942	0.0042	1.521	−	10
11	SDO^94^	1.73E−03	1.73E−03	4.37E−18	-	1.6639	1.74	15.928	0.004274	1.5203	−	10
12	AHT^95^	1.7298E−03	1.7298E−03	5.3923E−08	-	1.6639	1.738	15.92	0.0042	1.5203	−	2
13	DE^71^	1.7738E−03	2.15E−03	2.89E−04	3.12E−02	1.663	2.074	16.804	0.0037	1.539	−	12
14	GOTLBO^71,119^	1.846E−03	2.77E−03	3.41E−04	3.66E−02	1.663	2.347	17.432	0.0031	1.553	−	13
15	TSA^118^	1.876204E−03	-	-	-	1.6626	2.31	18.0516	0.00337	1.5523	−	14

#### Comparative analysis for the Photowatt-PWP201 module

**Figure 10:**
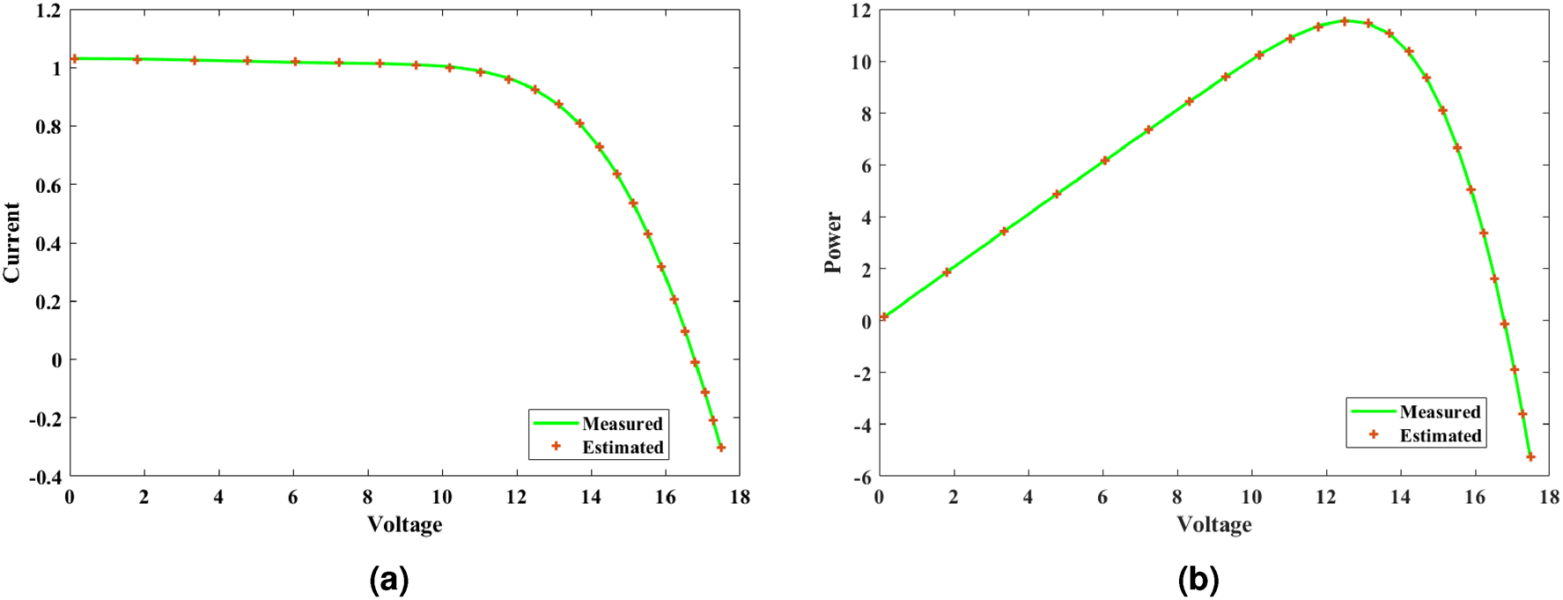
Comparisons of the (**a**) I-V (**b**) P-V characteristics for the Photowatt-PWP201 module between actual and simulated data obtained by PIFN.

The SDM of the Photowatt-PWP201 module’s five parameters^71,122^ has been extracted using PIFN, and the outcomes of this algorithm, which have been characterized by the lowest possible error, and also compared with various algorithms reported in the literature. The measured, as well as computed values (Ie, Im, V, Pe, Pm, IAE current, IAE Power, RE, MAE, MBE, and MAPE) of Photowatt-PWP201 module, are displayed in Tables  20. The best IAE current and IAE Power values are 4.35E−02 and 4.55E−01. The simulated behaviour of the I-V and the P-V utilizing the SDM result compared to the data utilized for parameters estimate is illustrated in Fig.  10a,b. In addition, the Table  21 illustrate the optimal model parameters, the RMSE, and the IAE obtained by the proposed PIFN and others algorithms reported in the literature for SDM. The best RMSE value is 2.06E−03.

**Table 20:** The PIFN outcomes of Ie, IAE, MAE, MBE, MAPE and RE for the Photowatt-PWP201 module.

S. no.	Ie	Im	IAE	V	Pe	Pm	IAE	RE
1	1.029066	1.0315	0.002434	0.1248	0.128427	0.128731	0.000304	0.00236
2	1.027109	1.03	0.002891	1.8093	1.858349	1.863579	0.00523	0.002807
3	1.025274	1.026	0.000726	3.3511	3.435797	3.438229	0.002432	0.000707
4	1.023477	1.022	0.001477	4.7622	4.874001	4.866968	0.007033	− 0.00145
5	1.021544	1.018	0.003544	6.0538	6.184222	6.162768	0.021453	− 0.00348
6	1.019134	1.0155	0.003634	7.2364	7.37486	7.348564	0.026296	− 0.00358
7	1.015619	1.014	0.001619	8.3189	8.448836	8.435365	0.013472	− 0.0016
8	1.009934	1.01	6.57E−05	9.3097	9.402186	9.402797	0.000611	6.5E−05
9	1.000427	1.0035	0.003073	10.2163	10.22066	10.25206	0.031397	0.003062
10	0.984809	0.988	0.003191	11.0449	10.87711	10.91236	0.035249	0.00323
11	0.960283	0.963	0.002717	11.8018	11.33307	11.36513	0.032068	0.002822
12	0.923969	0.9255	0.001531	12.4929	11.54305	11.56218	0.019128	0.001654
13	0.873633	0.8725	0.001133	13.1231	11.46478	11.4499	0.014872	− 0.0013
14	0.808217	0.8075	0.000717	13.6983	11.07119	11.06138	0.009817	− 0.00089
15	0.728499	0.7265	0.001999	14.2221	10.36079	10.33236	0.028431	− 0.00275
16	0.636521	0.6345	0.002021	14.6995	9.35654	9.326833	0.029707	− 0.00319
17	0.53527	0.5345	0.00077	15.1346	8.101095	8.089444	0.011652	− 0.00144
18	0.42802	0.4275	0.00052	15.5311	6.647622	6.639545	0.008077	− 0.00122
19	0.317676	0.3185	0.000824	15.8929	5.048799	5.061889	0.013089	0.002586
20	0.206866	0.2085	0.001634	16.2229	3.355971	3.382475	0.026504	0.007836
21	0.097558	0.101	0.003442	16.5241	1.612059	1.668934	0.056875	0.034078
22	− 0.00861	− 0.008	0.000607	16.7987	− 0.14459	− 0.13439	0.010196	− 0.07587
23	− 0.11091	− 0.111	9.43E−05	17.0499	-1.89093	-1.89254	0.001609	0.00085
24	− 0.20845	− 0.209	0.000553	17.2793	-3.60182	-3.61137	0.009555	0.002646
25	− 0.30067	− 0.303	0.002334	17.4885	-5.2582	-5.29902	0.040811	0.007702
IAE (Current)			0.04355	IAE (Power)			0.455868	
MAE		0.00174202	MBE		0.000109	MAPE		0.676628

**Table 21:** PIFN versus other algorithms on the Photowatt-PWP201 module under extracted parameter values.

S. no.	Optimization techniques	RMSE			IAE (Current)	Iph	Io1	RSh	Rs	n	p-rank	f-rank
		Min	Mean	Std.								
1	PIFN	2.06E−03	3.56E−03	1.27E−03	0.0435	1.0306	2.6043	868.6890	1.2391	46.6691		1
2	TSA^77^	2.4326E−03	-	-	-	1.0311	1.2004	912.4803	1.2191	48.6286	−	8
3	GAMNU^92^	2.382E−03	-	-	0.04132	1.0307	3.0162	906.2754	1.2191	48.0975	−	2
4	GSK^71^	2.42E−03	2.42E−03	1.04E−19	0.049932	1.0305	3.4823	981.982	1.2013	48.6428	−	3
5	HBA-OBL^85^	2.42E−03	2.42E−03	3.49E−08	0.0525	1.0305	3.4823	981.983	1.2013	48.6428	−	3
6	HBA^85^	2.42E−03	2.42E−03	1.69E−09	0.05325	1.0305	3.4823	981.987	1.2013	48.6428	−	3
7	BES^84^	2.42E−03	2.42E−03	2.45E−17	0.0547	1.0305	3.4823	981.982	1.2013	48.6428	−	3
8	DE^71^	2.42E−03	2.43E−03	1.25E−05	0.058921	1.0305	3.4823	981.982	1.2012	48.6848	−	3
9	JAYA^123^	2.43E−03	2.44E−03	1.25E−05	0.0462	1.0304	3.5622	970.174	1.1967	48.7315	−	8
10	TLABC^71,79^	2.43E−03	2.49E−03	1.12E−04	0.0507	1.0306	3.4715	972.935	1.2017	48.6313	−	8
11	BSA^122^	2.43E−03	2.49E−03	3.98E−05	0.0522	1.0306	3.2292	994.306	1.2118	48.3503	−	8
12	RTLBO^71,121^	2.43E−03	2.54E−03	4.98E−05	0.056832	1.303	3.5033	988.56	1.2006	48.66	−	8
13	GOTLBO^71,119^	2.43E−03	2.73E−03	7.25E−05	0.062379	1.0305	3.5214	984.656	1.1978	48.686	−	8

#### Statistical analysis

**Figure 11:**
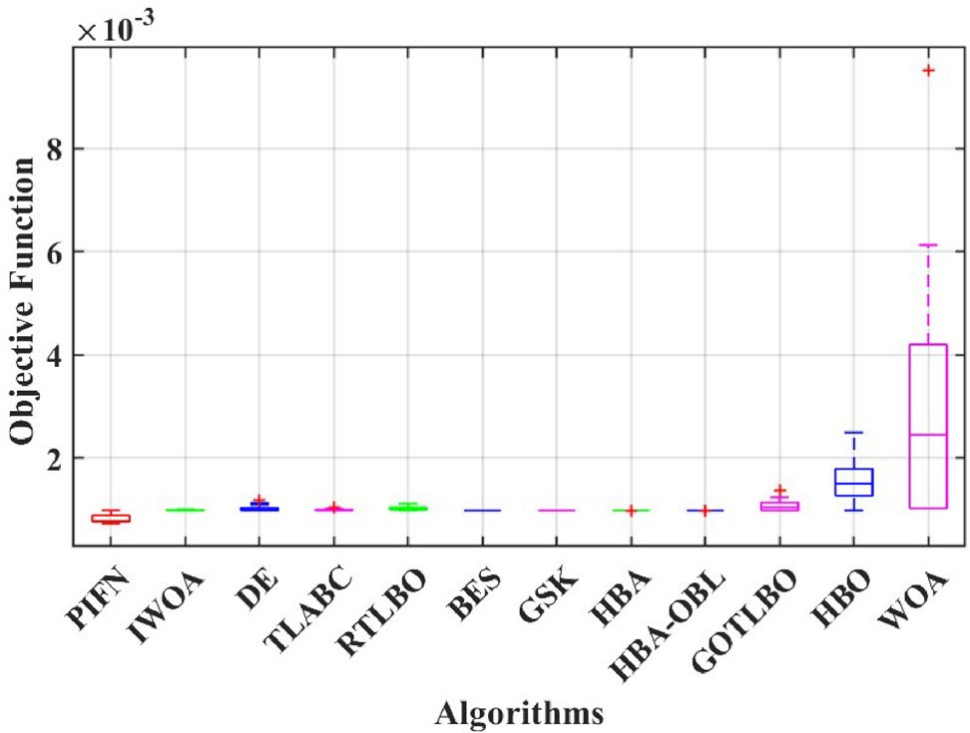
Boxplot for PV cells’ RTC France (SDM).

**Figure 12:**
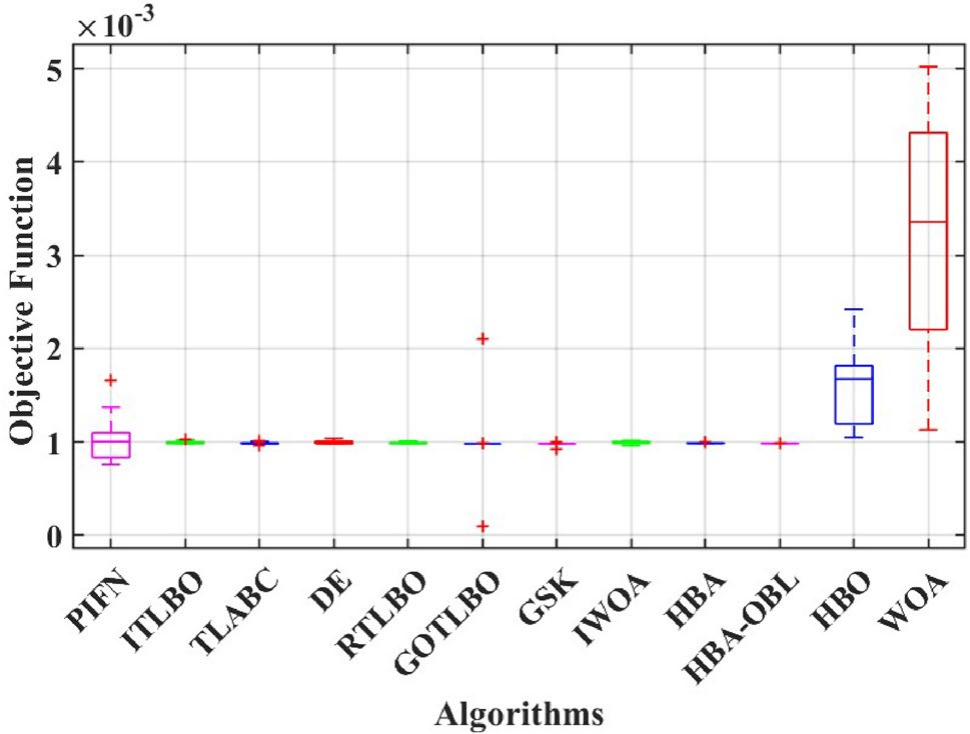
Boxplot for PV cells’ RTC France (DDM).

**Figure 13:**
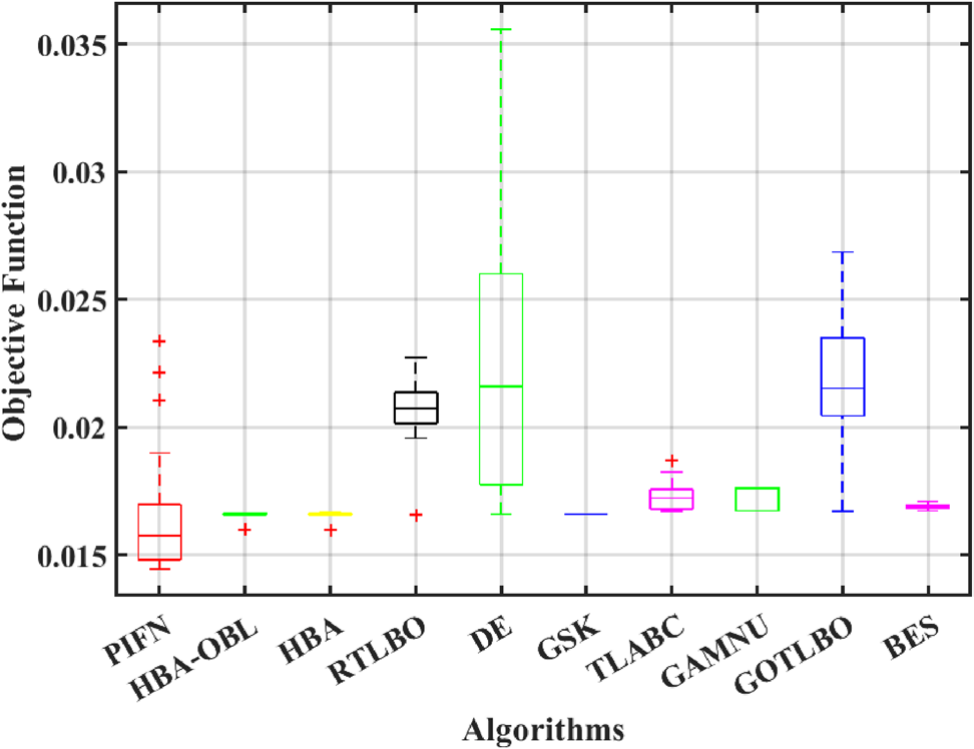
Boxplot for PV module STP6-120/36.

**Figure 14:**
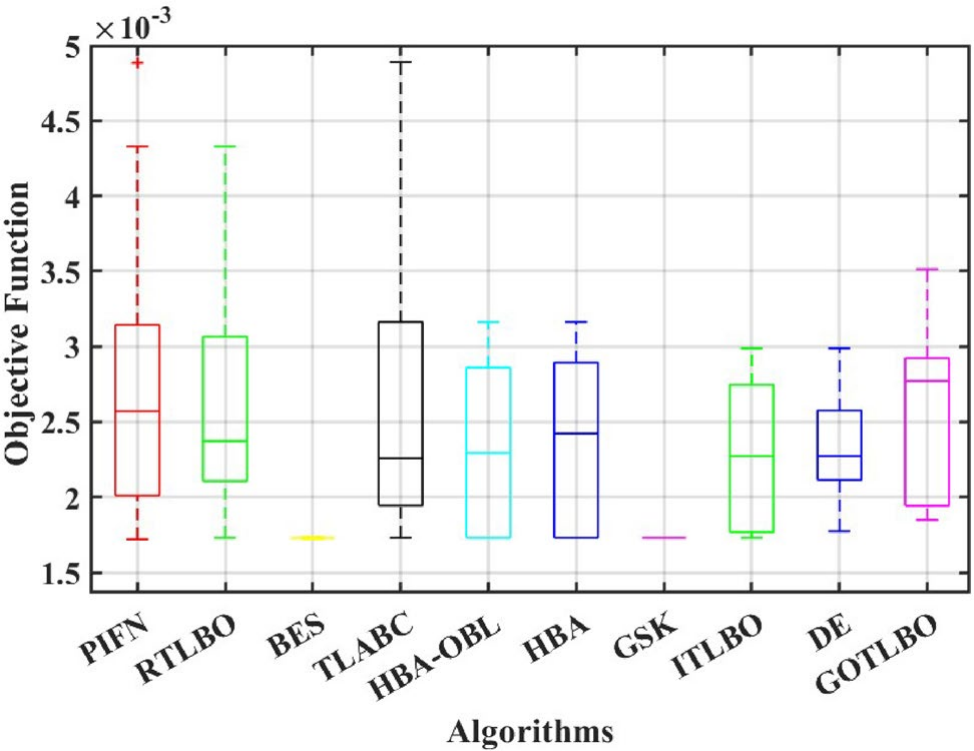
Boxplot for PV module STM6-40/36.

**Figure 15:**
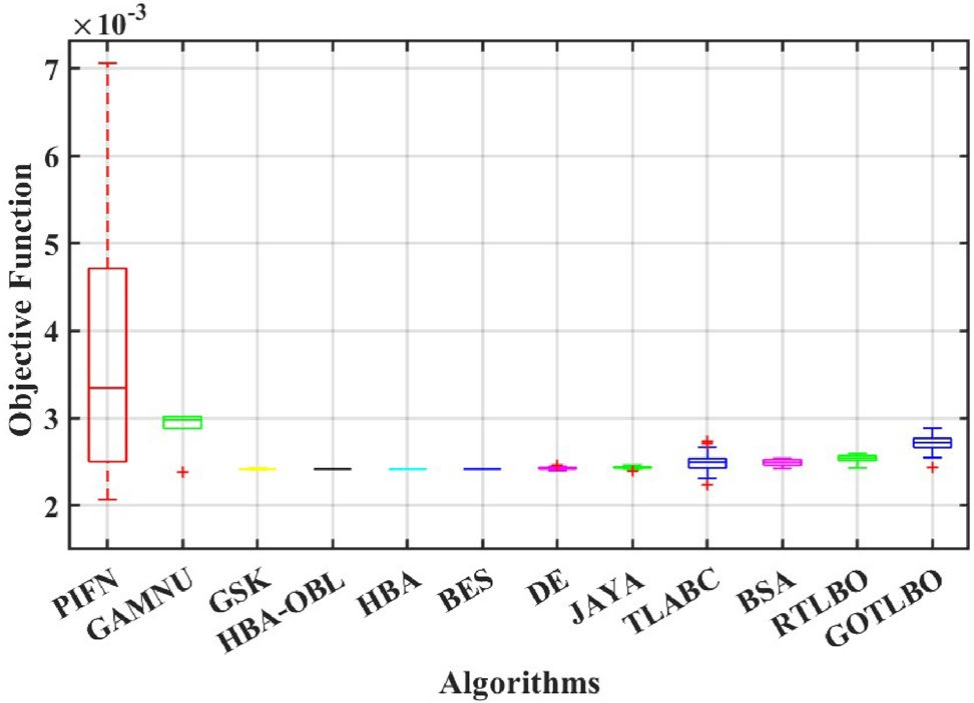
Boxplot for PV module Photowatt-PWP201.

The statistical performance of the PIFN is evaluated using two non parametric tests (Wilcoxon’s as well as Friedman’s rank-sum tests). The rank-sum test is used to determine the p-rank when comparing two algorithms’ performance. In contrast to the other MH algorithms that have been used for comparison, Wilcoxon’s rank-sum result demonstrates that the proposed PIFN is statistically more effective. Three situations will arise when the proposed algorithm in contrast to existing algorithms (loss(l)/win(w)/ tie(t)). First, the loss condition arises when the algorithm’s performance is the worst compared to the proposed algorithm and assigns a “-” sign. The win condition occurs when the algorithm’s performance is superior to the proposed algorithm and assigns a “+” sign. And the last case is the tie condition, which occurs when both the algorithm’s performance is significant to the proposed algorithm and assigns the “=” sign. So, l/w/ t values are displayed in Tables  13,  15,  17,  19, and  21. The statistical test known as the Friedman rank (f-rank) assigns f-rank to each optimization algorithm being evaluated. It is simple to evaluate whether or not the proposed PIFN performs significantly better by comparing its performance to that of other MH optimization techniques using f-rank. The Tables  13,  15,  17,  19, and  21 illustrates the f-rank of each algorithm for each test function. The PIFN is ranked top among all other algorithms for the the RTC France Solar Cell (SDM and DDM), Photowatt-PWP201, STM6- 40/36, and STP6-120/36 module problems and is ultimately determined to be a statistically significant optimization algorithm. Also, Figs. 11, 12, 13, 14, and 15 illustrations boxplot graph for the RTC France Solar Cell (SDM, and DDM), Photowatt-PWP201, STM6- 40/36, and STP6-120/36 module. It can be demonstrates from Figs. 11, 12, 13, 14, and 15 that the proposed PIFN algorithm achives the best outcomes in terms of reliability as well as accuracy compared to the other MH algorithms

## Discussion of results

In this section, we present details on the proposed algorithm and the outcome of results. This section consists of three parts including summary of results,the drawback of PIFN as well as some insightful implications of PIFN algorithm. we provide a comprehensive discussion as

### Summary of results

In this section, we present details on the results obtained and is given by

- With the added advantages of different algorithms, and adaptive parametric details, it has been found that the best set of mutation operators include simulated annealing, exponentially decreasing, and linearly decreasing inertia weights provide the best combination of parameters.
- Population size and dimension size comparison show that a population size of 50 gives the best results, as increasing the population size further above increases the computational complexity of the algorithm, but the results do not vary too much. But when we reduce the population size, the diversity in search agents reduces significantly. For dimension size analysis, as the problem complexity increases, the results do degrade, but in comparison to other algorithms, PIFN provides much better results.
- The PIFN algorithm was superior to the MHA, EO, GWO-E, LSHADE-SPACMA, JADE, SaDE, CMA-ES, OEWOA, LSHADE-SPACMA, FA-FPO, as well as SCCSA algorithms on the classical benchmark problems, including unimodal and multimodal functions.
- For CEC-2017 benchmark challenges including uni-modal, multi-modal, and composition challenges, the PIFN algorithm outperformed the SaDE, JADE, SHADE, MVMO, CS, $CV_{new}$, and CV1.0 algorithms.
- Apart from that, when comparing with respect to CEC 2019 benchmarks, our proposed PIFN algorithm achieves better performance than jDE100, YDSE, DE, FPA, and NMRA algorithms.
- The better performance of the PIFN algorithm over these multi-modal, hybrid and composite functions shows that the exploration properties of the proposed algorithm are excellent. A better performance of uni-modal functions shows that the algorithm can achieve better exploitation. Finally, the performance on fixed dimensional functions shows that the algorithm can achieve better-balanced operation and good convergence patterns.
- In addition to the benchmark problems, parameter estimation of solar PV (RTC France Solar Cell (SDM, and DDM), Photowatt-PWP201, STM6- 40/36, and STP6-120/36 module) using PIFN algorithm demonstrates that the proposed algorithm provides significantly better results compared to other MH algorithms.
- Overall, the better performance of PIFN is due to the presence of multi-hybridization, self-adaptation and local optima avoidance characteristics.

### Drawbacks

- Although, the proposed PIFN algorithm is found to provide highly reliable results, but a poor exploration and exploitation balance is still to be exploited. Also, due to the added advantages, worker phase is exploited too much for performance enhancements, but breeder phase is still to be explored, and more enhanced exploitation operation must be added.
- The proposed PIFN algorithm also has an improved stagnation phase, but it is very critical to decide when should this phase trigger. A deeper analysis regarding the activation of stagnation phase must be done. Because in its current stages, there are chances that the algorithm might get stuck in this phase and the basic structure might have little or no internal capabilities.
- Also, PIFN lacks a proper balanced exploration and exploitation phase and a lot of work is required to be done to incorporate the same. This is better understood from the fact that the convergence patterns of the algorithm are not that great and more rigorous studies need to be done.
- The PIFN algorithm performed worse than the MHA, CMA-ES, EO, LSHADE-SPACMA, JADE, SaDE, FA-FPO, LSHADE-SPACMA, and SCCSA algorithms on the fixed dimension challenges of the classical benchmarks.
- Apart from that, the comparative analysis reveals that the PIFN algorithm shows inferior performance when compared to the SaDE, JADE, SHADE, MVMO, and $CV_{new}$ algorithms in tackling the hybrid challenges encountered in the CEC 2017.

### Insightful Implications

- Although the PIFN algorithm has certain disadvantages, but the results have shown that it can serve as a basic algorithm for other domain research problems and this is because of its simple structure, easier implementation, and self-adaptive nature.
- A proper balance between exploration as well as exploitation needs to be added to make the algorithm the best fit for most of real-world optimization problems.
- New domains of research can be explored by making the algorithm binary and multi-objective in nature. The algorithm can then be applied to linear antenna arrays, electroencephalogram, and other problems.
- A deeper analysis of stability, convergence properties, and other empirical studies can also be conducted to see how well the algorithm works. Apart from that, theoretical studies can also be done for better understanding of the proposed PIFN algorithm.

## Conclusion

This paper presents a multi-hybrid algorithm based on the added properties of PDO, INFO, FuFiO, as well as NMRA. A new stagnation phase is added by taking inspiration from SACS, and it is meant to ensure that the algorithm does not get stuck in local optima as well as keep moving toward the global solution. Three benchmark sets classical problems, CEC 2017, and CEC 2019 are added to check for the better performance of the algorithm and its significance with respect to other MH algorithms. A detailed study about the effect of population size as well as dimension size is also added to see the significance of the proposed study statistically. Five different mutation/inertia weight operators were exploited to make the algorithm self-adaptive in nature. The proposed algorithm (PIFN) performs significantly better than GWO, NMRA, JADE, jDE100, LSHADE-SPACMA, CMA-ES, and other improved algorithms. Freidmann as well as Wilcoxon’s ranksum tests further very the importance of the proposed PIFN algorithm statistically. The algorithm has been also applied for parameter estimation of solar PV (RTC France Solar Cell (SDM, and DDM), Photowatt-PWP201, STM6- 40/36, and STP6-120/36 module). Here, also the proposed PIFN algorithm performs significantly better with respect to other recently introduced algorithms under consideration. For future works, we can apply the algorithm to various engineering design challenges including economic load dispatch problem, antenna arrays, clustering and others. Local and global search properties of the algorithm can be enhanced. Also, parametric analysis must be done to see what fits the best for the problem under consideration.

## Data Availability

The datasets used and/or analysed during the current study available from the corresponding author on reasonable request.
